# CRISPR spacers reveal diverse and abundant Thermococcales viruses in hydrothermal vents

**DOI:** 10.21203/rs.3.rs-8799458/v1

**Published:** 2026-02-19

**Authors:** Ryan Catchpole, Justin McLean, Emily St. John, Anna-Louise Reysenbach, Mart Krupovic, Michael P. Terns

**Affiliations:** 1 University of Georgia, Athens, GA, USA; 2 Portland State University, Portland, OR, UGA; 3 Institut Pasteur, Université Paris Cité, Cell Biology and Virology of Archaea Unit, 75015, Paris, France

## Abstract

Viruses are the most pervasive biological entities on Earth and they profoundly shape host ecology and evolution. However, for many microbial lineages, knowledge of their viromes remains limited, especially for those inhabiting remote environments, including deep-sea ecosystems. Here, we leverage one of the most extensively cultivated and genomically characterized archaeal lineages, the Thermococcales, to identify novel viral genomes. By utilizing CRISPR spacers from isolates and spacer arrays reconstructed from metagenomes, we mined mobile genetic elements (MGEs) in 1,172 publicly available and newly sequenced hydrothermal vent metagenomic datasets. Comparative genomics and identification of viral hallmark proteins revealed 620 viral genomes across 19 taxonomic families, most of which were previously undescribed. Structural modeling of major capsid proteins (MCPs) revealed diverse virion morphotypes, including viruses with spindle-shaped, head-tailed, icosahedral, filamentous, ovoid and bacilliform virions, greatly expanding the previously limited Thermococcales virome. Family-level comparisons uncovered extensive flux of virus-encoded replication proteins that are evolutionarily and structurally distinct from host homologs, as well as dramatic variation in glycan-binding lectins suggestive of diverse infection strategies. Together, our results substantially broaden the Thermococcales virosphere and demonstrate the power of combining cultivated isolates with culture-independent, CRISPR-guided metagenomics to interrogate archaeal virus diversity and evolution.

## Introduction

The order Thermococcales comprises hyperthermophilic Archaea spanning three genera: *Pyrococcus*, *Thermococcus*, and *Palaeococcus*^1^. These obligately anaerobic sulfur reducers are common in hydrothermal vents, exhibiting optimal growth temperatures of 75–100°C, and are recognized as reservoirs of thermostable enzymes for industrial and biotechnological applications^2^. Furthermore, the genetic tractability and cultivation of several representative strains have made them powerful model organisms for understanding key aspects of both hyperthermophile and archaeal biology, including DNA replication^3^, gene expression and stress response^4^, and metabolism^5^. Consequently, Thermococcales have been isolated extensively over several decades, leading to large strain collections and an unusually rich body of isolate-derived genome sequences.

Thermococcales have also emerged as model systems for studying archaeal immunity, which protects against diverse mobile genetic elements (MGEs), including viruses and plasmids. For example, argonaute proteins in *Pyrococcus furiosus* inhibit plasmid invasion^6^ and have since been harnessed for the detection and programmable cleavage of nucleic acids for a wide variety of applications^7^. Studies of Thermococcales have also been instrumental in elucidating various stages of CRISPR-Cas immunity, including the generation of immune ‘memories’ from MGE DNA in the form of spacers^8,9^, processing of CRISPR transcripts to generate guide RNAs^10^, and cleavage of foreign nucleic acids by effector nucleases^11^.

The near-ubiquitous presence of CRISPR-Cas systems among Thermococcales, together with extensive CRISPR arrays, suggests sustained pressure from a diverse and dynamic mobilome. Indeed, MGEs are pervasive in these organisms, and a survey of ~200 isolates revealed ~40% harbor at least one extrachromosomal element^12^. However, the set of characterized Thermococcales MGEs is overwhelmingly dominated by plasmids^13^, ranging from small cryptic plasmids <5 kbp harnessed for laboratory vectors^14–16^, to large conjugative plasmids >100 kbp capable of self-transmission^17^.

In contrast to this abundance of plasmids, the known virome of Thermococcales is sparse, especially relative to the many phages targeting bacteria and even viruses of other archaea, such as Sulfolobales^18^ and Halobacteriales^19^. Only two viruses capable of producing virions have been reported within *Thermococcales*: PAV1, isolated from *Pyrococcus abyssi* GE23^20,21^, and TPV1, isolated from *Thermococcus prieurii*^22^. These two viruses produce spindle-shaped virions and appear to exist in a stable carrier relationship with their hosts. Consistent with related viruses of Sulfolobales^23^, PAV1 and TPV1 virions are released without causing host-cell lysis. A further virus-like element, pTN3, has been described in *Thermococcus nautili*^24^, with several related elements integrated in genomes such as TKV4 in *Thermococcus kodakarensis*^25–27^; however, despite encoding a predicted major capsid protein, these elements have not been shown to produce virus-like particles. Thus, despite extensive study of Thermococcales, only two bona fide viruses have been previously characterized, raising the question as to whether this apparent scarcity reflects biological reality or limitations of current isolation approaches.

Importantly, to date, the discovery and characterization of Thermococcales MGEs, both viral and non-viral, has historically been carried out by examining cultivated isolates. While useful, these solely culture-based methods necessarily select against virulent MGEs (e.g. lytic viruses or those conferring a growth deficit), as well as those infecting hosts that do not grow well under laboratory conditions. Recent advances in metagenomic sequencing have generated an unprecedented volume of environmental sequence data, enabling microbial and viral diversity to be explored beyond the constraints of cultivation-dependent approaches^28^. Within this expanded metagenomic landscape, CRISPR spacers offer a host-linked strategy for identifying infectious MGEs hidden in large-scale sequencing datasets, as they retain a sequence-identical record of prior infections. This approach has been successfully applied across diverse archaeal lineages^29–31^, yet its reach is ultimately shaped by the availability and diversity of CRISPR spacer datasets.

Here, we assembled a comprehensive dataset of nearly 40,000 CRISPR spacers derived from cultivated Thermococcales, augmented with spacers recovered from metagenomic assemblies, and used these to mine both publicly available and newly generated hydrothermal vent metagenomes worldwide. This approach identified more than 3,000 Thermococcales-associated MGEs, including 620 predicted viral genomes spanning 19 candidate viral families, greatly expanding the known diversity and evolutionary breadth of the Thermococcales virome.

## Methods

### Dataset collation

We downloaded all metagenome datasets containing the keyword “hydrothermal” from the JGI IMG database^32^ on June 30, 2023. Metagenome accession numbers are provided in Supplementary Table 1. We generated an additional 70 deep-sea hydrothermal vent metagenomes (DSV70)^33^ from which we assembled ~5,000,000 assembled contigs (Supplementary Table 2). All published Thermococcales plasmid and viral genomes were downloaded from the NCBI RefSeq database on June 20, 2023. In addition, we extracted integrated MGEs from Thermococcales genomes as described^34^.

### Metagenome community profiling

We predicted 16S rRNA gene sequences in metagenomic assemblies using Barrnap (v0.9) (https://github.com/tseemann/barrnap). We used the predicted sequences as queries in BLAST+ (v2.14.1)^35^ against the 16S rRNA database, and assigned taxonomy based on the top-scoring hit.

### Thermococcales CRISPR spacer datasets

We predicted spacers in complete Thermococcales genomes using the CRT plugin (v1.1) for Geneious Prime v2023^36^. To identify metagenomic Thermococcales CRISPR arrays, we predicted repeats in complete Thermococcales genomes using MinCED (v0.4.2)^36^, and collated a set of unique repeat sequences (available in Supplementary Table 3). We used MinCED to predict all CRISPR arrays in the metagenomic datasets, restricting valid repeats to lengths of 29–31 nt (all Thermococcales repeats are 29–31 nt). The set of metagenomic repeats was restricted to those with at most 1 mismatch to a Thermococcales genomic repeat, or its reverse complement, using AGREP (v3.41.5)^37^. Finally, we extracted spacers from the MinCED-identified arrays.

### Spacer-protospacer matching and candidate MGE selection

We used published and predicted CRISPR spacers as BLASTn search queries against the metagenome datasets with the blastn-short option. We identified candidate Thermococcales MGEs from contigs of length ≥2 kbp that exhibited ≥2 protospacer matches at ≥90% identity, or ≥1 protospacer match when MGE completeness could be predicted (see below). We extended spacer-targeted contigs using ContigExtender^38^ where raw JGI sequencing reads were available. Manual curation was performed to remove artifactual extensions, such as concatamers and homopolymers.

### Prediction of full-length MGE genomes

We analyzed all contigs using the CheckV^39^ end-to-end workflow to identify terminal repeats indicative of full-length genomes. We manually verified these using the “Find repeats” tool in Geneious Prime 2023 (allowing ≤10% mismatches for repeats ≥20 bp). Finally, we deduplicated contigs using the CheckV aniclust.py script^39^ by clustering at ≥98% ANI and ≥95% coverage.

### Gene-sharing analysis and community detection

We predicted open reading frames (ORFs) using Prodigal^40^ with the -p meta flag. We clustered the resultant proteins using MMseqs2^41^ at a 25% amino acid identity threshold (Supplementary Table 5). We constructed a gene sharing network in Cytoscape v3.10.0^42^ where nodes represent contigs and edges connect those sharing ≥5 MMseqs2 clusters. We inferred community structure using the OSLOM algorithm^43^ with the Community Detection plugin, utilizing total shared genes as a weight parameter and a P-value = 0.2.

### Genome annotation and major capsid protein identification

We generated a multiple sequence alignment (MSA) from each MMseqs2 cluster using MUSCLE v3.8.31^44^. We then created a profile hidden Markov model (HMM) from each MSA using HHsuite v3.3.0^45^. We queried HMMs using HHblits against the PDB70, pfamA_35, uniprot_sprot_vir70, and phrogs_v4; hits with probability ≥90% were considered significant. Additionally, annotations were generated using DRAM-bio v1.4.0^46^, DIAMOND 2.1.9^47^, and BLASTp against the NCBI nonredundant (nr) database (e ≤1E-5), and batch CD search against the NCBI conserved domain database (CDD). Transmembrane domains were predicted using TMHMM v2.0^48^ (Supplementary Table 6).

We predicted structures of cluster representatives (from MMseqs2 clustering) using AlphaFold3^49^ and functions were predicted with FoldSeek v9-427df8a^50^ using PDB, UniProt, UniProt50, Proteome, and Swiss-Prot databases. Representatives returning prokaryotic virus capsid/coat proteins among the top 10 FoldSeek hits were further evaluated by DALI searches against the PDB. Proteins showing unambiguous sequence and/or structural similarity to known prokaryotic virus MCPs were considered significant.

### Viral taxonomy and comparative genomics

Contigs that reached ≥75% of the length of the shortest complete genome within an OSLOM community were treated as representative sequences. We analyzed relatedness using ViPTree^51^, alongside all ICTV-recognized exemplars (MSL40v1) for the major viral morphotypes including spindle-shaped, head-tail, icosahedral, filamentous, ovoid, and bacilliform viral families. When morphotypes contain large numbers of ICTV exemplars e.g. head-tail or icosahedral viruses, VipTree was run on the entire sequence set, the resulting tree visualized, and trees re-generated using only examplars closely related to the Thermococcales contigs.

We generated synteny diagrams for family representatives using Clinker^52^. All putative viruses are described in Supplementary Table S4 are sequences are available in Supplementary Data 1.

### Phylogenetic analyses

We extracted a previously described set of viral and cellular PolB sequences^53^, limiting each clade to 10 random representative sequences (except for clade B3, where all sequences were retained to detect potential host-virus transfer). We added all Thermococcales viral PolB sequences to this dataset. Following alignment with MAFFT (v7.526)^54^, we removed non-informative positions using BMGE (v2.0)^55^. We generated a phylogenetic tree using IQ-TREE (v2.3.6)^56^ with the LG+F+R8 model. Trees were visualized in iTol (v7.2.1)^57^ and annotated in Adobe Illustrator (v25.4.1). We predicted PolB protein structures using Alphafold3 and visualized them with PyMol (v3.1.5.1)^58^.

We downloaded the set of MCM sequences used in Krupovic et al. 2010^59^ and Thermococcales viral MCM sequences were added to this dataset. Phylogenies and structures were resolved as for PolB, above.

### Lectin protein analysis

We collated and aligned lectin-domain containing proteins using MAFFT (v7.526). Structures were predicted with Alphafold3 and visualized in PyMol (v3.1.5.1).

### Biogeography

We inferred sample locations of viral contigs from metagenomic metadata (Supplementary Tables 1+2). Viral contig abundance by location was visualized in QGIS and plotted according to the predicted viral family.

## Results

### Targeted metagenome sequencing enriches for Thermococcales

**Figure 1.**
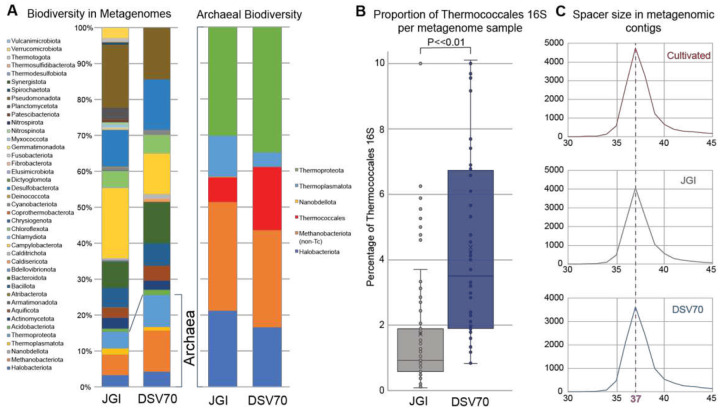
Comparison of metagenomic sequences in published databases (JGI) and newly sequenced datasets (DSV70). A) Analysis of 16S rRNA gene biodiversity in all contigs shows similarity in the phyla present in both datasets, with an enrichment for Archaea in the DSV70 samples. Measuring only archaeal 16S rRNA genes shows enrichment for Thermococcales in the DSV70 metagenomes. B) When measured on an individual metagenome scale, DSV70 metagenomes are significantly enriched in Thermococcales compared to JGI datasets (Mann–Whitney U, P=5×10–14). Samples devoid of Thermococcales 16S rRNA genes are excluded. C) Size distribution of spacers extracted from Thermococcales CRISPR arrays is identical between genomes from cultivated organisms and metagenomic sources

To characterize the mobilome associated with Thermococcales, we curated hydrothermal vent metagenomes from the Joint Genomes Institute Integrated Microbial Genomes and Microbiomes database (JGI IMG/MER) by downloading all datasets containing the keyword “hydrothermal” (n=1102). To evaluate Thermococcales representation, we predicted 16S rRNA gene sequences in assembled contigs and quantified the fraction assigned to Thermococcales. The resulting JGI metagenomes were highly diverse, containing 16S rRNA gene sequences spanning 41 taxonomic classes, with Thermococcales comprising an average of only ~1% of the 16S rRNA gene sequences (Figure 1A). Many datasets contained no identifiable Thermococcales 16S rRNA gene sequences, while a small subset showed substantially higher representative abundance (Figure 1B).

The JGI hydrothermal vent metagenomes were heterogenous both in geography and sample type (e.g. marine sediments and diffuse vent fluids). Although Thermococcales have been isolated from vent sediments^60^ and vent fluids^61^, most Thermococcales isolates originate from hydrothermal chimney fragments^62^ where cells form biofilms within porous chimney precipitates^63^. Their detection in diffuse fluids or surrounding sediments may therefore reflect dispersal from primarily chimney-associated communities.

To prioritize environmental samples representative of the true growth habitat of these organisms, we sequenced 70 additional metagenomes from homogenized chimney fragments and hydrothermal rocks collected from multiple deep-sea vent sites^33^ (Supplementary Table 2). These datasets are referred to as DSV70 samples throughout. The DSV70 metagenomes showed comparable overall biodiversity to those of the JGI set (16S rRNA gene sequences spanning 37 taxonomic classes) but were enriched in Thermococcales, reaching up to 4% of the community in some samples (Figure 1B). Both JGI and DSV70 sets contained representatives of all recognized Thermococcales genera (*Pyrococcus*, *Palaeococcus*, and *Thermococcus*,) including all three *Thermococcus* clades defined by GTDB-Tk (Supplementary Fig. 1).

### Thermococcales encode a large CRISPR spacer repertoire

To identify Thermococcales-associated MGEs, we leveraged the natural ability of CRISPR arrays to catalog sequences from infecting elements. We extracted 15,342 unique CRISPR spacers from the genomes of cultivated Thermococcales strains. In parallel, we identified CRISPR arrays in the JGI and DSV70 metagenomes and extracted all spacers from arrays with repeats matching cultivated Thermococcales repeats with ≤1 mismatch (a stringent filter that retrieves only Themococcales CRISPR arrays from the entire GenBank nt database). Metagenomic spacers displayed a size distribution similar to those of isolated strains (Figure 1C), with a median length of 37 bp, consistent with functional crRNAs observed *in vivo*^64^. Reinforcing the enrichment of Thermococcales in the DSV70 datasets, we recovered nearly the same number of unique spacers (n=11,800) from the 27.5 Gbp of DSV70 metagenomes as from the 191 Gbp of the JGI dataset (n = 11,977). For comparison, Thermococcales genomes yielded >15,000 spacers from 0.2 Gbp of sequence. Combining all sources produced a total of 39,177 unique Thermococcales CRISPR spacers.

### CRISPR-guided discovery of the Thermococcales-associated mobilome

We searched the combined JGI and DSV70 metagenomes for contigs >2 kbp containing protospacer matches to the total spacer set. Contigs targeted by ≥2 spacers at ≥90% identity, or by ≥1 spacer where MGE completeness could be inferred by terminal repeats, were added to our dataset of Thermococcales MGEs. After manual curation to remove likely host genome fragments and CRISPR arrays, and after adding of extrachromosomal and integrated elements from cultivated genomes, we obtained 3,074 contigs that we classify as putative Thermococcales MGEs, or fragments thereof. This set includes 195 contigs inferred to be complete MGE genomes, based on direct or inverted terminal repeats.

Consistent with Thermococcales enrichment in the DSV70 samples, 77% of spacer-targeted contigs originated from DSV70 metagenomes. Overall, protospacer matches were identified for 33% of spacers, and each contig was matched by an average of 14 spacers (range 1–293). Predicted proteins encoded by the Thermococcales MGEs clustered into 5,625 protein families, highlighting extensive Thermococcales mobilome diversity.

**Figure 2.**
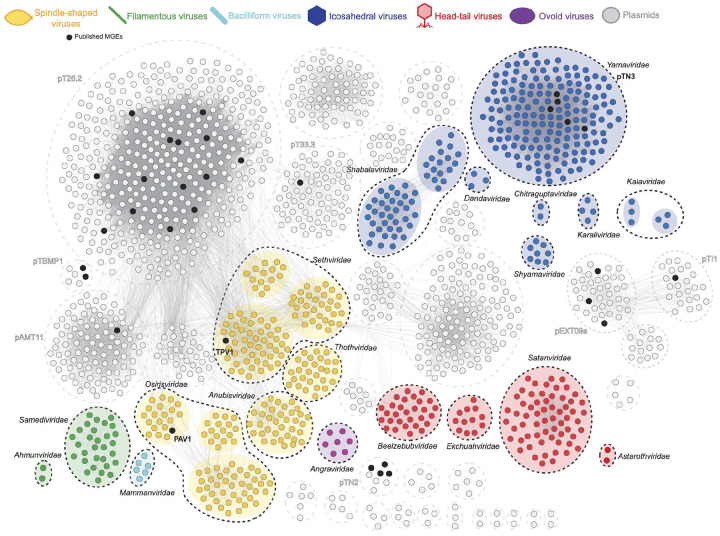
Gene-sharing network of the Thermococcales mobilome. Circular nodes indicate individual MGEs/contigs, with those contigs sharing at least five protein clusters connected by a grey line. Black nodes represent published MGEs and names are indicated (if published). Predicted viral contigs are colored by morphological class, as indicated by the key at top. Statistically significant viral node clusters are contained within a colored circle, with black dashed lines indicate proposed taxonomic families. Predicted plasmid contigs are represented by grey nodes, and statistically significant plasmid node clusters are indicated by grey dashed lines.

To classify MGEs and assess relationships between them, we constructed a gene-sharing network in which contigs (nodes) were connected by edges (lines) if they shared ≥5 protein clusters (Fig. 2). Community analysis revealed cohesive groups of densely connected contigs defined by conserved core genes, which were similarly targeted by CRISPR spacers from published Thermococcales genomes and JGI and DSV70 metagenome sources (Supplementary Fig. 2). We then screened core genes of each MGE community for homologs of the major capsid proteins (MCPs), a hallmark of viruses, using both sequence-based annotation and structure-based similarity. Communities encoding core proteins with unambiguous sequence or structural homology to known MCPs were classified as putative viruses, whereas communities lacking predicted MCPs were classified as probable plasmids. Below, we focus on the predicted Thermococcales viruses.

### Major capsid proteins define 19 candidate families of Thermococcales viruses

Analysis of the core proteins across the Thermococcales mobilome identified 19 communities (n=620 contigs) encoding conserved MCP homologs, indicative of viruses infecting Thermococcales. These virus communities can be assigned to one of five major archaeal virus morphotypes, each characterized by a distinct MCP topology/fold^65^. Spindle-shaped and bacilliform viruses of Thermococcales encoded highly hydrophobic, α-helical hairpin MCPs typical of archaeal spindle-shaped viruses^66^. The predicted filamentous viruses encoded unique α-helical MCPs diagnostic of the virus realm *Adnaviria*^67^. Predicted head-tailed viruses of the class *Caudoviricetes* (realm *Duplodnaviria*) are defined by an HK97 fold MCP together with the terminase large subunit (TerL) and portal protein^65^. Tailless icosahedral viruses encode either single jelly-roll (SJR; realm *Singelaviria*) or double jelly-roll (DJR; realm *Varidnaviria*) MCPs^68^. We also identified a group of putative Thermococcales viruses related to the ovoid virus, *Sulfolobus* ellipsoid virus 1 (SEV1), the sole representative of the family *Ovaliviridae*^69^.

Notably, all but one viral community contained at least one representative with a complete genome, enabling estimation of fragmentation and completeness across related contigs. To evaluate whether these viruses represent novel taxa or expansions of known groups, we clustered sequences against the prokaryotic viral RefSeq dataset using vConTACT2^70^. With a few exceptions, Thermococcales viral contigs did not cluster with known viral genomes, consistent with extensive novelty (Supplementary Fig. 3). Overall, these data greatly expand three previously described Thermococcales virus groups (TPV-like, PAV1-like, and pTN3-like elements) and add 16 previously undescribed families of Thermococcales viruses. We named these families after gods and demons associated with hells or underworlds of various religious traditions, reflecting the abyssal nature of hydrothermal vents.

To formally delineate viral family-level groupings, we generated proteomic trees for the representative Thermococcales viral contigs together with ICTV reference sequences representing each of the morphotypes. For head-tailed archaeal viruses, a ViPTree distance cut-off of ~0.05 has been used to approximate family-level groupings^71,72^. Using this criterion, we defined three Thermococcales head-tailed virus families: “*Satanviridae*,” “*Astarothviridae*,” and “*Beelzebubviridae”* (Supplementary Fig. 4). In addition, two near-complete Thermococcales head-tailed genomes grouped within *Ekchuahviridae*, a family of head-tailed viruses previously associated with Methanophagales hosts in hydrothermal ecosystems^30^.

Although no single distance cut-off is established for tailless icosahedral viruses, proteomic trees indicate that most family-level clades fall between a distance of 0.01 and 0.05. Based on shared gene content and a ViPTree distance of ~0.03, we defined five candidate families of tailless icosahedral viruses: “*Yamaviridae”* (including pTN3 and related integrated elements^24,26^), “*Shabalaviridae*,” “*Shyamaviridae*,” “*Karaliviridae*,” and “*Chitraguptaviridae”* (Supplementary Fig. 5). Spindle-shaped viruses formed four candidate families: “*Sethviridae*” (including TPV1^22^), “*Osirisviridae”* (including PAV1^20^), “*Anubisviridae”*, and “*Thothviridae”* (Supplementary Fig. 6). Filamentous viruses within *Adnaviria* comprised one additional candidate viral family, *Samediviridae*, and expanded the existing family *Ahmunviridae* (Supplementary Fig. 7), which also includes viruses associated with Methanophagales hosts from the same hydrothermal vent metagenomes as members of *Ekchuahviridae* (mentioned above)^30^. ViPTree further supported the novelty of a bacilliform virus family “*Mammanviridae*,” and the ovoid virus family “*Angraviridae*” (Supplementary Fig. 8). A detailed description of each virus family alongside their genome maps is provided in Supplementary Text.

Collectively, these results greatly expand the Thermococcales virome and provide evidence that Thermococcales are targeted by diverse virus morphotypes. Notably, head-tailed viruses, tailless icosahedral viruses, bacilliform viruses, and ovoid viruses have not been previously linked to Thermococcales.

### Thermococcales viruses span diverse genomic architectures and inferred lifestyles

The three Thermococcales viruses described to date (TPV1, PAV1, and the virus-like element pTN3) persist in stable association with host cells, either through chromosomal integration (TPV1 and pTN3), or as episomal elements (PAV1). In contrast, the newly identified viruses likely encompass a broader range of infection strategies. For example, members of the non-enveloped head-tailed viruses of the “*Satanviridae”* family lack recognizable integrases or recombinases to facilitate chromosomal integration and are likely to be obligately lytic and lethal to infected host cells. Consistent with strong selection imposed by lethal infections, “*Satanviridae”* contigs show the highest CRISPR targeting density, averaging ~3.5 spacers per kbp (Supplementary Fig. 9), including one viral genome targeted by >200 distinct spacers (Supplementary Text S2A).

Tailless icosahedral viruses are also frequently associated with host lysis during virion release^73–75^. However, the largest family, “*Yamaviridae*,” uniformly encodes an integrase, suggesting that chromosomal integration and lysogeny are common within this lineage. Indeed, “*Yamaviridae”* include the integrative pTN3 in *T. nautili*^26^, and the integrated element TKV4 in *T. kodakarensis*^27^. Spindle-shaped viruses are generally released without host lysis^23^. Although the conserved core gene set of “*Osirisviridae”* (including PAV1) does not universally include an integrase, some representatives encode integrase genes, indicating that osirisviruses may establish persistent infections through both integrative and non-integrative strategies (as in the case of PAV1^20^).

Across many viral families, genomes exhibited a conserved modular architecture, with core structural genes clustered together and a large variable region elsewhere in the genome, often bounded by DNA replication modules e.g. “*Sethviridae”* (Supplementary Text S1B), “*Thothviridae”* (Supplementary Text S1C), “*Satanviridae”* (Supplementary Text S2A), “*Shabalaviridae”* (Supplementary Text S6D). Similar “core + variable” architectures are common in Thermococcales plasmids^76,77^ and in bacteriophages^78^, consistent with strong constraints on core functions alongside an accessory gene repertoire. In our data set, variable regions frequently encode numerous small ORFs with no confident functional annotation, suggestive of a pool of uncharacterized accessory genes. Similar variable regions in bacteriophage genomes encode small anti-defense proteins^79^, and it is likely that the variable regions of Thermococcales viruses encode similar proteins that act to disarm host defenses, among other functions that promote viral infection.

Many Thermococcales viral families encode multiple predicted transmembrane proteins within their conserved cores, particularly spindle-shaped viruses (Supplementary Text S1A–D). These viruses may need to express membrane-bound proteins for host interaction, superinfection exclusion, or assembly/egress pathways that require membrane-associated components^80^.

### DNA replication modules are highly exchangeable in spindle-shaped virus families

Comparative genomics revealed substantial diversity in DNA replication modules, most prominently among spindle-shaped viruses. Replication loci encode either a minichromosome maintenance (MCM) helicase, an archaeo-eukaryotic primase (AEP) with separately encoded helicase (often UvrD-like superfamily 1 helicases), a primasehelicase fusion protein, or a rolling circle replication initiation endonuclease resembling those found in small cryptic plasmids (e.g. pRT1^81^). Even closely related members within the same viral family often encode distinct replication strategies (see Supplementary Text S1B and S1D for examples), suggesting frequent horizontal exchange of replication modules. Notably, these modules imply mechanistically distinct DNA replication modes (theta-like vs rolling circle), suggesting that major shifts in replication strategy are tolerated in these families of viruses.

**Figure 3.**
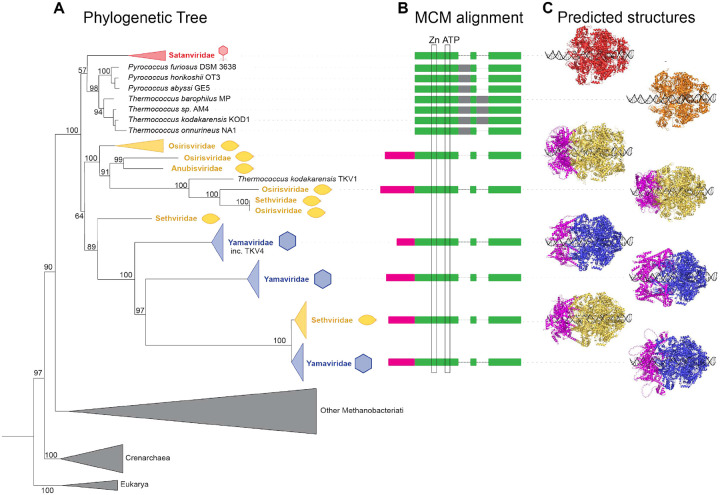
Relationship of Thermococcales MCM proteins. A) Maximum likelihood phylogenetic tree of MCM protein sequences, rooted with Eukarya outgroup, and branch support indicated. Thermococcales genomic MCMs are named in black text, with viral families in colored text according to their morphotypes (see Figure 2). Other archaeal clades are collapsed (grey triangles) for simplicity. B) Schematic of protein sequence alignment with shared MCM regions indicated in green, inteins in grey, and viral N-terminal extensions in magenta. Zinc finger and Walker A+B motifs are indicated by black boxes labelled Zn and ATP, respectively. C) AlphaFold3 structural predictions of DNA-bound homohexamers are shown with representatives for each clade indicated in viral morphotype colors (cellular MCM in orange) and magenta regions indicating viral N-terminal extensions.

MCM helicase-like genes were present in six Thermococcales virus families, spanning spindle-shaped, icosahedral, and (more rarely) head-tailed viruses. MCM is the replicative helicase in archaea and eukaryotes^82,83^, but is also widespread among archaeal MGEs. MGE-encoded MCMs have been acquired repeatedly via recent host-to-MGE transfers in Methanococcales^59^. In contrast, phylogenetic reconstruction suggests that Thermococcales viral MCMs were acquired early, consistent with an origin predating the last common ancestor of all Thermococcales (Figure 3A). Frequent exchange of MCM genes was apparent among spindle-shaped virus families (“*Osirisviridae”*, “*Anubisviridae”*, and “*Sethviridae”*) consistant with shared hosts and opportunities for co-infection and MCM gene recombination. Within “*Sethviridae”* and “*Yamaviridae”*, MCM evolution appears to have been especially complex. Members of “*Sethviridae”* appear to encode a basal MCM variant that is ancestral within Thermococcales viral clade and was transferred to a “*Yamaviridae”* ancestor. More recently, a subset of “*Sethviridae”* appears to have replaced the ancestral MCM variant with a “*Yamaviridae”*-derived MCM protein, indicating potential for co-infection of hosts by viruses from different viral families (Figure 3A).

In contrast to cellular MCMs, many Thermococcales viral MCMs also carry an additional N-terminal extension of ~200 aa (Figure 3B,C), predicted to form a DNA-binding helixturn-helix domain. Similar extensions were noted previously in two of three MCMs encoded by *T. kodakarensis*^84^, both of which derive from integrated MGEs^59,85^, including TKV4 (assigned here to “*Yamaviridae”*). In cellular DNA replication, Orc1/Cdc6 proteins recruit MCM to origins of replication; however, we did not identify Orc1/Cdc6-like proteins encoded by Thermococcales viral genomes. We therefore hypothesize that viral MCMs combine origin recognition and helicase activity via acquisition of an N-terminal DNA binding domain. Consistent with this model, the N-terminal winged helix-turn-helix domain present in viral MCMs is structurally similar to the DNA recognition domain of archaeal Orc1/Cdc6^86^ (Supplementary Fig. 9).

### Diverse Viral DNA polymerases

**Figure 4.**
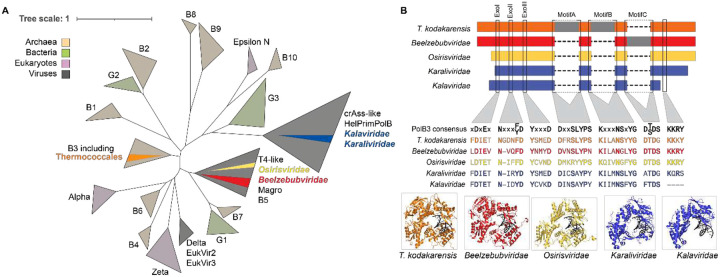
Diversity of viral DNA polymerases. A) Unrooted phylogenetic tree of PolB proteins including Thermococcales viral MCMs. Domain source of PolB clades are indicated by the key (top left). Thermococcales genomic PolBs are indicated in saturated orange, with viral families in text and segments colored according to their morphotypes (see Figure 2). Thermococcales viral PolBs group with other viral sequences (grey clades) rather than cellular PolBs (orange clades). B) Schematic of protein sequence alignment with common MCM regions indicated in morphotype colors (cellular PolB in orange), with inteins in grey. Conserved motifs are indicated by labelled black boxes, with residues indicated below relative to PolB3 consensus. AlphaFold3 predictions of DNA-bound PolB structures are shown below.

We also detected family B DNA polymerases (PolB) in several viruses, particularly in head-tailed “*Beelzebubviridae”* (Supplementary Text S2C). Although these polymerases are structurally similarity to Thermococcales PolB proteins, they are only distantly related at the sequence level (Fig. 4A). Cellular Thermococcales PolBs belong to the PolB3 clade of archaeal B-family polymerases involved in DNA repair^53,87^. In contrast, Thermococcales viral PolBs clustered with diverse viral polymerase lineages (Fig. 4A). PolBs from “*Beelzebubviridae”* and “*Osirisviridae”* grouped with PolB from Magroviruses predicted to infect Marine Group II Euryarchaeota^88^, whereas PolBs from “*Karaliviridae”* and “*Kalaviridae”* grouped with polymerases from crAssphage-like viruses infecting Bacteroidetes bacteria^89^.

Thermococcales viral PolBs are shorter than cellular counterparts (after intein removal), and sequence alignments and structure predictions indicate reductions in the N-terminal domain and (in some cases) a shortened or absent C-terminal helical bundle (Fig. 4B). Despite these differences, all viral PolBs retained conserved catalytic motifs required for polymerase function^90^ and proofreading exonuclease activity^91^ (Fig. 4B). Several viral PolBs lacked the N-terminal uracil-sensing domain implicated in aborting replication on uracil-containing templates^92^. “*Kalaviridae”* PolB lacked a C-terminal motif implicated in stabilizing incoming DNA in the entry channel^93^, although Alphafold3 prediction with an extended DNA substrate suggests alternate structural elements may support substrate DNA stabilization (Supplementary Fig. 10). Given their unique structural features and predicted thermostability, these polymerases represent attractive candidates for biochemical characterization and potential biotechnological applications.

In addition to canonical DNA replication genes, we observed cases in which the replication-module locus was also replaced by genes not obviously linked to replication. For example, “*Yamaviridae”* typically encode an MCM helicase, but in some genomes this locus was replaced by a small helical protein with a ribbon-helix-helix (RHH)-like domain (Supplementary Text S6A). Similarly, in “*Osirisviridae”* the locus encodes an MCM helicase in OsirisV10, or a pRT1-like rolling circle replication endonuclease in OsirisV6, whereas in PAV1 the same region encodes a DUF7845-containing protein with a C-terminal helix-turn-helix domain (Supplementary Text S1A). Although the roles of these proteins are unclear, their positional conservation suggests that they may functionally substitute for DNA replication control or origin-associated processes.

### Thermococcales viruses encode diverse predicted glycan-binding proteins

The archaeal cell envelope, typically a cytoplasmic membrane plus a para-crystalline proteinaceous surface (S-) layer^94^, presents a formidable barrier for viral entry and egress. Although surface glycosylation in Thermococcales remains poorly characterized, staining and histology indicate the presence of cell-surface glycans^95^. Some archaeal viruses encode enzymes implicated in glycan binding, modification or cleavage^96,97^, supporting a role for surface glycans in virus-host interactions^98^. Genome sequencing of the only two isolated Thermococcales viruses, TPV1 and PAV1, revealed two concanavalin A-like proteins containing predicted lectin domains^20–22^.

With our massively increased dataset of Thermococcales viral genomes, we identified a diverse set of proteins containing predicted lectin (glycan binding) domains across the majority of Thermococcales viral families. In spindle-shaped viruses, two lectin-domain containing proteins with predicted transmembrane helices formed part of the conserved core in all four families (“*Sethviridae”*, “*Osirisviridae”*, “*Thothviridae”*, and “*Anubisviridae”*) (Supplementary Text S1A–D). Outside of the spindle-shaped virus families, lectin domain proteins were also core genes in the icosahedral “*Shabalaviridae”* (Supplementary Text S6D) and occurred widely as accessory genes in other viral families.

**Figure 5.**
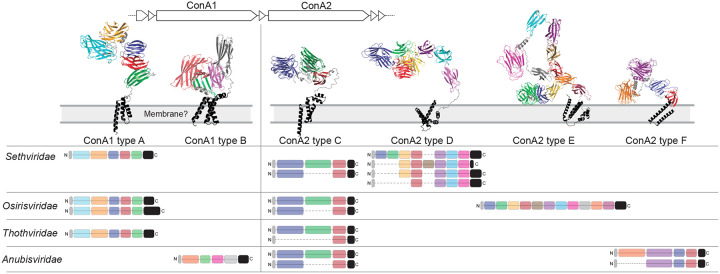
Modular lectin-domain proteins of spindle-shaped viruses. The two conserved concanavalin-like proteins of all Thermococcales spindle-shaped viruses are indicated schematically at top. Left: ConA1 proteins are conserved across *“Sethviridae”*, *“Osirisviridae”*, and *“Thothviridae”*, with each encoding 5 lectin domains and a predicted transmembrane domain, indicated by colored blocks in protein sequence alignment. AlphaFold3 predictions indicate lectin domains with colors corresponding to schematic alignment. ConA1 proteins of *“Anubisviridae”* differ (ConA1 type B), comprised of 4 lectin domains and a transmembrane domain. Right: ConA2 proteins are more diverse and modular. 4 broad protein types are identified and arbitrarily labelled ConA2 types C-F. Each ConA2 type shows modularity across viral genomes, with gain/loss of lectin domains indicated by presence/absence of colored blocks in schematic protein alignments, corresponding with colored domains in predicted structures.

To better resolve lectin diversity in the spindle-shaped viral families, we predicted protein structures using Alphafold3 and clustered lectin domains by sequence and structural similarity. These analyses indicated that the lectin protein ConA1 is conserved among “*Sethviridae”, “Osirisviridae”*, and “*Thothviridae”*, whereas ConA2 is highly variable, exhibiting repeated insertion/deletion of lectin domains and occasional replacement by non-homologous lectin proteins (Fig. 5). “*Anubisviridae”* encode a distinct lectin protein at the ConA1 locus but share similarities at the ConA2 locus (Fig. 5). The modularity of these proteins at both the protein and domain level suggests rapid diversification that may track variation in host surface glycans. We speculate that lectin domain repertoires contribute to host specificity by recognizing species- or strain-specific glycan structures.

### Biogeography

**Figure 6.**
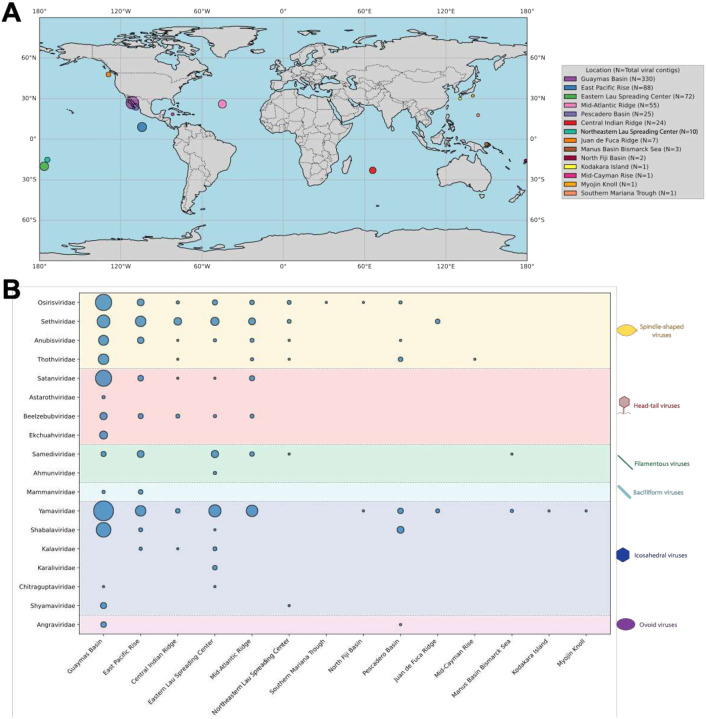
Biogeography of Thermococcales viruses. A) Sampling locations in which Thermococcales viral contigs were identified are plotted with colored dot size correlated to number of viral genomes recovered. Viruses were identified across hydrothermal sites worldwide. B) Plotting viral families against sampling sites reveals little family-level endemism, with all well-represented families present across distant sampling sites. Colored regions of plot correspond to viral morphotypes; circle size correlates to relative frequency of identified viral contigs.

To assess the geographic distribution of the Thermococcales virome across globally distributed hydrothermal vents, we quantified viral contig abundance by site, morphotype, and family (Fig. 6). Metagenomes from Guaymas Basin samples contained viral contigs spanning all predicted morphotypes and were the dominant source for spindle-shaped, head-tail, and ovoid virus families. Guaymas Basin also contributed many icosahedral virus contigs, whereas the highest family-level diversity of icosahedral viruses was observed in metagenomes from the Eastern Lau Spreading Center (ELSC) deep-sea vents. Both these sites have reported an abundance of Thermococcales^99,100^, and hence the viromes track the biodiversity observations.

Most viral families were detected across multiple and often geographically distant locations (Fig. 6). “*Yamaviridae”*, the most widely distributed family, was common across Atlantic, Pacific, and Indian Ocean sites, as were “*Osirisviridae”*, “*Sethviridae”*, “*Satanviridae”*, and “*Beelzebubviridae”*. “*Samediviridae”*, despite being a substantially smaller family than those mentioned above, was detected in nearly half of sampled locations. A subset of families appeared restricted to single sites (e.g. “*Astarothviridae”* and *Ekchuahviridae* in Guaymas Basin; “*Karaliviridae”* and *Ahmunviridae* in Valu Fa Ridge), although limited sampling could contribute to apparent endemism. “*Angraviridae”* was detected only in Guaymas Basin and nearby Pescadero Basin, and “*Mammanviridae”* was restricted to Guaymas Basin and East Pacific Rise. Collectively, these results highlight the lack of biogeographical structuring of the Thermococcales virome, with many virus families being distributed across geographically remote sites.

## Discussion

Our CRISPR-guided metagenomic survey substantially transforms our understanding of the Thermococcales virosphere, expanding it from a few isolated examples to a diverse landscape of at least 19 candidate families. Despite decades of intensive study and the near-ubiquitous presence of CRISPR–Cas systems with extensive spacer repertoires, only two bona fide viruses and one virus-like element had previously been described in these organisms^20,22^. By searching global hydrothermal vent metagenomes with isolate- and metagenome-derived Thermococcales CRISPR spacers, we show that Thermococcales are routinely targeted by a large and diverse assemblage of viruses (Fig. 2). These viruses span all major archaeal virion morphotypes and fall into at least 19 divergent taxonomic families, 16 of which we define here for the first time. These findings resolve a long-standing paradox between the extensive CRISPR immune investment of Thermococcales and the apparent scarcity of their viruses. Our work demonstrates that this discrepancy reflected limitations of discovery rather than biology and reveals a rich, ancient virosphere associated with one of the most intensively studied archaeal lineages.

Recent studies have revealed hundreds of thousands of viral contigs across a variety of ecosystems by identifying viral hallmark genes and structural protein signatures in metagenomic sequence data^101–103^. These approaches have transformed our view of global viral diversity, but they necessarily bias discovery toward viruses with hallmarks resembling previously characterized lineages. This limitation is particularly acute for archaeal viruses, whose capsid architectures and genome organizations are often highly divergent^104^ and, in some cases, only recognizable after virion structures have been resolved^66^. CRISPR-guided discovery provides a complementary approach by anchoring metagenomic sequences to documented biological interactions, revealing MGEs that have infected specific hosts regardless of whether they encode recognizable viral hallmarks. Our work builds upon a growing body of CRISPR-guided viromics that has recently uncovered viral diversity linked to archaeal hosts^29–31,105,106^, applying this strategy here to a rich collection of cultivated Thermococcales genomes to directly connect viral discovery to a specific host lineage.

The stark disparity between the few recorded Thermococcales viruses and the many isolated viruses of Sulfolobales^107^ and Halobacteriales^19^, other cultivated and well-studied archaeal lineages, underscores how strongly accessibility and sampling have shaped our view of archaeal virology. Sulfolobales and Halobacteriales viruses were isolated from easily accessible hot spring fluids and high-salinity pools, respectively, that can be filtered to concentrate virions^108^, whereas Thermococcales inhabit chimney interiors that are poorly sampled by traditional virological approaches. Our data indicate that Thermococcales are not virus-poor; rather, their virome has been largely missed by decades of cultivation-dependent discovery. Moreover, Thermococcales-associated viruses were particularly enriched in metagenomes derived from porous interiors of hydrothermal vent chimneys (DSV70), the primary growth habitat of Thermococcales^62,63^. This pattern highlights how sampling strategies that capture organism-specific microhabitats can substantially expand access to biologically relevant MGEs that are underrepresented in more diffuse environmental surveys.

The breadth of virion architectures uncovered here further underscores this point. Prior to this study, Thermococcales were known only to host the spindle-shaped viruses PAV1 and TPV1 (as well as pTN3-like virus-derived elements). We now show that they are targeted by head–tailed viruses, tailless icosahedral viruses of both SJR and DJR lineages, filamentous viruses of the realm *Adnaviria*, bacilliform viruses, and ovoid viruses related to *Ovaliviridae* (Fig. 2). Importantly, this expanded repertoire more closely mirrors the diversity of archaeal virus morphotypes observed in other lineages^18^ and demonstrates that Thermococcales are fully embedded in the broader evolutionary landscape of archaeal virology. As in other metaviromics studies, our classification of Thermococcales-associated MGEs depends on recognizable viral hallmarks, and it is therefore likely that some contigs labeled as plasmids (Fig. 2) represent viruses whose capsids remain unidentified, and that the diversity of Thermococcales viruses is even greater than currently recognized.

Beyond structural diversity, Thermococcales viruses exhibit extensive modularity at the level of genome replication. Even within closely related viral families, replication modules diverge extensively (Supplementary Text S1A–D). This is consistent with frequent horizontal exchange of replication genes during coinfection and long-term persistence within dense microbial communities. In Thermococcales viruses, capsid architecture and lifestyle appear to define stable evolutionary lineages, whereas replication strategies are readily exchanged, allowing viruses to adapt to changing intracellular environments while preserving virion identity. The evolutionary history of viral MCM helicases provides a striking example of this process (Fig. 3), mirrored by similarly ancient and virus-specific PolB lineages within these families (Fig. 4). Unlike the MCMs of Methanococcales MGEs, which appear to have been repeatedly acquired from hosts by recent horizontal transfer^59^, Thermococcales viral MCMs form a deeply rooted clade that predates the diversification of modern Thermococcales. This observation is consistent with long-term divergence from host MCMs, rather than recurrent host-to-virus transfer. The acquisition of N-terminal DNA-binding domains in most Thermococcales viral MCMs further suggests functional specialization for viral genome replication. The lack of canonical origin-recognition factors such as Orc1/Cdc6 in our viral dataset, and the lack of classical ORB sequences suggests that these MCM DNA-binding domains may function in origin recognition (Supplementary Fig. 10). Although archaeal chromosomal origins are classically recognized by monomeric or dimeric Orc1/Cdc6 prior to hexameric MCM loading^86^, eukaryotic ORC–Cdc6 forms a hexameric DNA-encircling complex^109^, providing a structural precedent for direct origin recognition by ring-shaped origin-binding assemblies such as those predicted for these viral MCMs (Fig. 3).

A second major axis of diversification lies in host cell surface recognition. We observed widespread conservation of lectin-like proteins in the cores of multiple viral families (Fig. 5; Supplementary Text S1A–D). Although these predicted sugar-binding proteins are invariably present in spindle-shaped viruses, they are highly variable between genomes, even those of otherwise closely related viruses. In bacteriophages, receptor-binding proteins are among the most rapidly evolving components of the virion^110^. Our data suggest that Thermococcales viruses follow a similar strategy, with lectin repertoires adapting to the diverse glycan landscapes of archaeal S-layers. The frequent gain and loss of complete lectin domains from viral-encoded proteins may indicate Thermococcales surface glycans evolve through similar modular gains and losses of sugar moieties. Elucidation of Thermococcales cell surface glycans will be important in understanding these virus-host interactions.

Despite the geographic separation of hydrothermal vent systems, most viral families were detected across multiple oceans (Fig. 6). This lack of strong biogeographic structuring contrasts with patterns observed for other hydrothermal vent viruses, where endemism dominates over dispersal^111^. Closely related Thermococcales have been recovered from geographically distant vent sites^112^, likely owing to their ability to survive cold oxygenated water by assuming a state of dormancy^113^. It is thus likely that Thermococcales-associated MGEs disperse and colonize distant vent sites alongside their hosts.

With our findings, Thermococcales now occupy a position in archaeal virology analogous to that long held by Sulfolobales and Halobacteriales: a genetically tractable lineage with a rich virome that can be interrogated mechanistically.

## Supplementary Material

1

Supplementary Files

This is a list of supplementary files associated with this preprint. Click to download.

- TableS1S62126.xlsx
- SupplementaryData1Viruscontigsall.txt

## Data Availability

All metagenome data is available from JGI or GenBank with accession numbers listed in Supplementary Tables 1 and 2. All viral sequences are available in the Supplementary Data 1.
