# Supplementary material for: CRISPR spacers reveal diverse and abundant Thermococcales viruses in hydrothermal vents
Source: Res Sq. 2026 Feb 19:rs.3.rs-8799458. Preprint. [Version 1] doi: 10.21203/rs.3.rs-8799458/v1 (PMC12934983; doi:10.21203/rs.3.rs-8799458/v1)
Supplement: 1 [file NIHPPrs8799458V1-supplement-1.pdf]

SUPPLEMENTARY FIGURES

“CRISPR spacers reveal diverse and abundant Thermococcales viruses in hydrothermal vents”

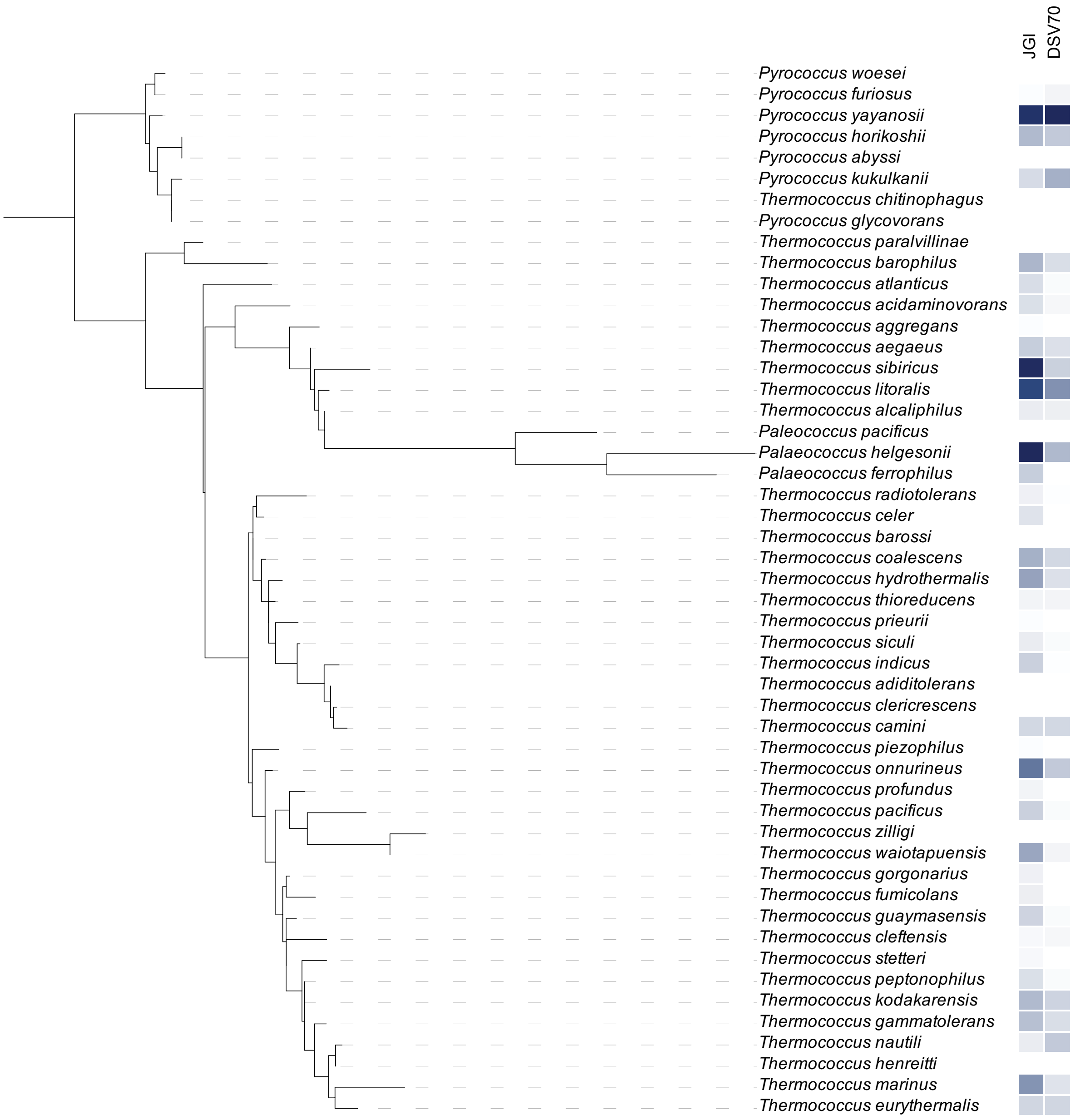

JGI
DSV70
Pyrococcus woesei
Pyrococcus furiosus
Pyrococcus yayanosii
Pyrococcus horikoshii
Pyrococcus abyssi
Pyrococcus kukulkanii
Thermococcus chitinophagus
Pyrococcus glycovorans
Thermococcus paralvillinae
Thermococcus barophilus
Thermococcus atlanticus
Thermococcus acidaminovorans
Thermococcus aggregans
Thermococcus aegaeus
Thermococcus sibiricus
Thermococcus litoralis
Thermococcus alcaliphilus
Paleococcus pacificus
Palaeococcus helgesonii
Palaeococcus ferrophilus
Thermococcus radiotolerans
Thermococcus celer
Thermococcus barossi
Thermococcus coalescens
Thermococcus hydrothermalis
Thermococcus thioreducens
Thermococcus prieurii
Thermococcus siculi
Thermococcus indicus
Thermococcus adiditolerans
Thermococcus clericrescens
Thermococcus camini
Thermococcus piezophilus
Thermococcus onnurineus
Thermococcus profundus
Thermococcus pacificus
Thermococcus zilligi
Thermococcus waiotapuensis
Thermococcus gorgonarius
Thermococcus fumicolans
Thermococcus guaymasensis
Thermococcus cleftensis
Thermococcus stetteri
Thermococcus peptonophilus
Thermococcus kodakarensis
Thermococcus gammatolerans
Thermococcus nautili
Thermococcus henreitti
Thermococcus marinus
Thermococcus eurythermalis

Supplementary Fig. 1. Comparison of Thermococcales 16S species prevalence in metagenomic sequences in published databases (JGI) and newly sequenced datasets (DSV70). Phylogenetic tree based on RpoB protein of all complete Thermococcales. Presence of species indicated by blue colored squares with pale blue indicating rare presence among Thermococcales 16S sequences, to dark blue indicating common occurrence. Both JGI and DSV70 metagenome datasets contain species from all clades of Thermococales, with similar species presence between the datasets.

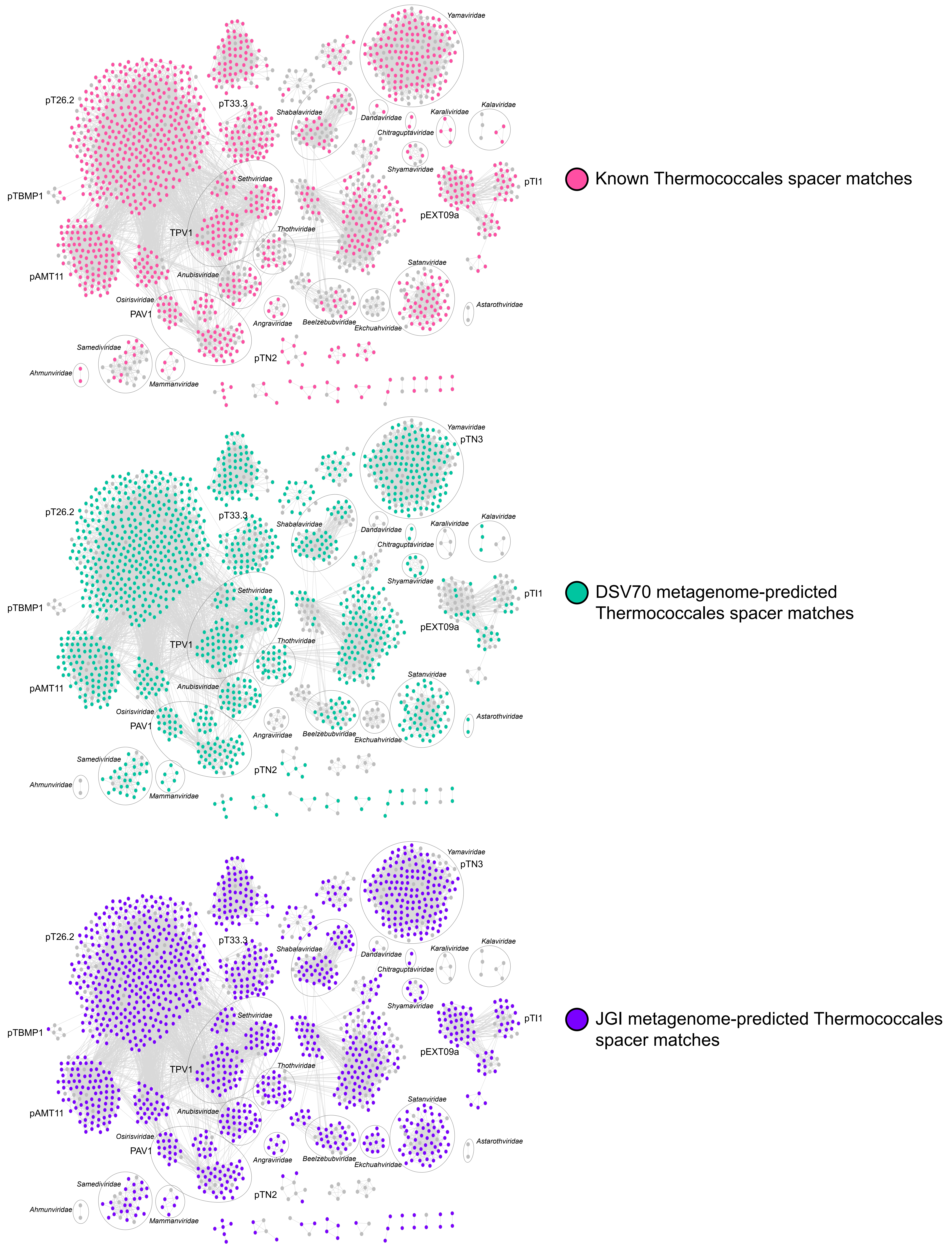
pT26.2
pT33.3
pTN3
Yamaviridae
Shabalaviridae
Dandaviridae
Karaliviridae
Kalaviridae
Chitraguptaviridae
Shyamaviridae
pTBMP1
Sethviridae
pTI1
pEXT09a
TPV1
Thothviridae
pAMT11
Anubisviridae
Satanviridae
Osirisviridae
PAV1
Angraviridae
Beelzebubviridae
Ekchuahviridae
Astarothviridae
Samediviridae
pTN2
Ahmunviridae
Mammanviridae
Known Thermococcales spacer matches
DSV70 metagenome-predicted Thermococcales spacer matches
JGI metagenome-predicted Thermococcales spacer matches

Supplementary Fig. 2. Comparison of MGE contigs detected vs spacer source. Gene sharing network displayed as in Fig. 2, with contigs colored if they are targeted by spacers from A) Sequenced Thermococcales isolate genomes (pink), B) DSV70 metagenome-derived Thermococcales arrays (green), or C) JGI metagenome-derived Thermococcales arrays (purple). All 3 spacer sources target each MGE family similarly.

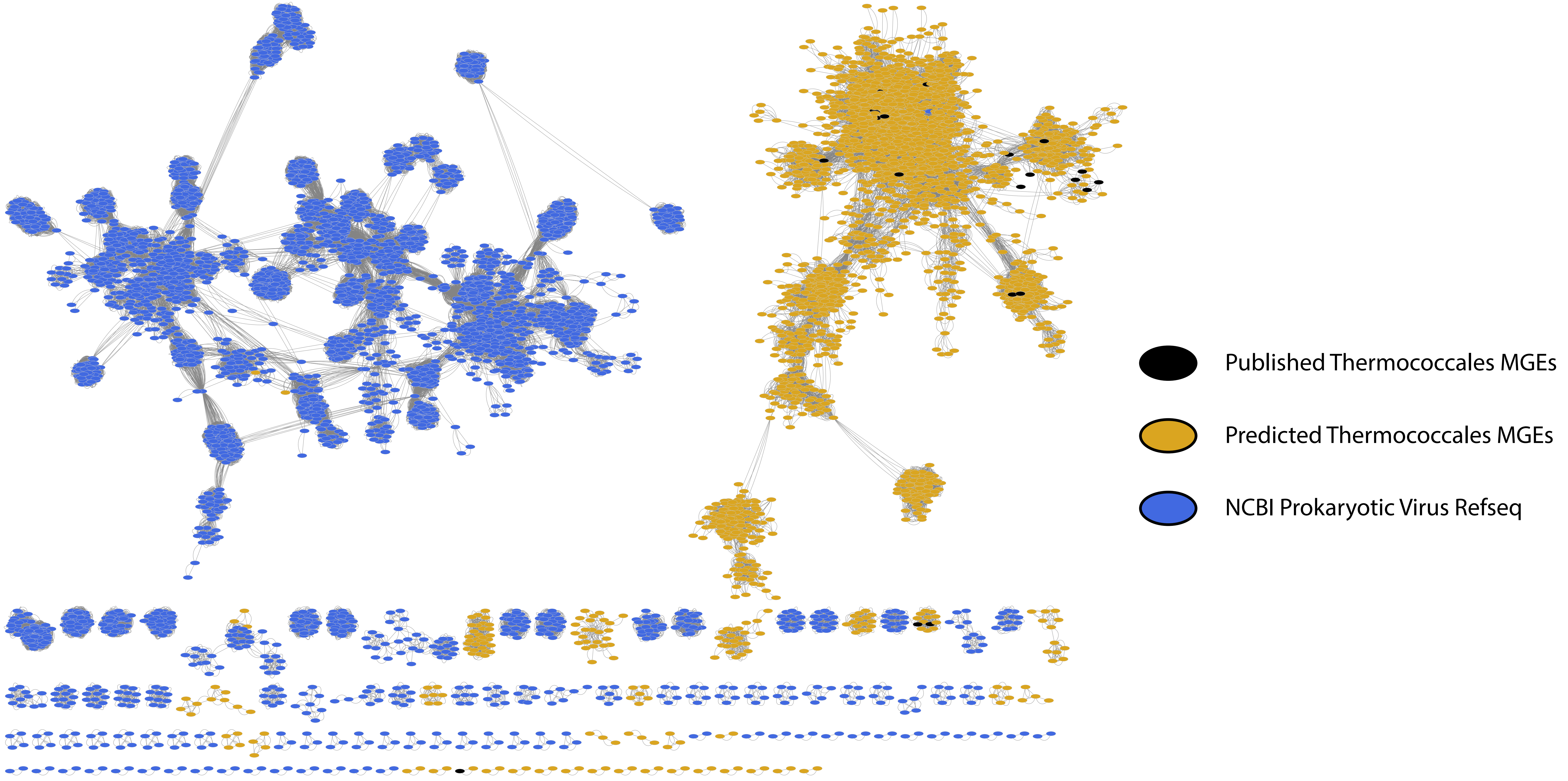

Published Thermococcales MGEs
Predicted Thermococcales MGEs
NCBI Prokaryotic Virus Refseq

Supplementary Fig. 3. A gene-sharing network generated by vConTACT2, with significantly similar genomes (nodes) connected by lines (edges). Published Thermococcales MGEs are denoted by black nodes. Predicted Thermococcales MGEs or fragments identified in this study are denoted by gold nodes. Sequences contained in the ProkaryoticViralRefseq211 database are denoted by blue nodes. With few exceptions, the Thermococcales MGEs recovered in our work are significantly divergent from all known prokaryotic MGEs.

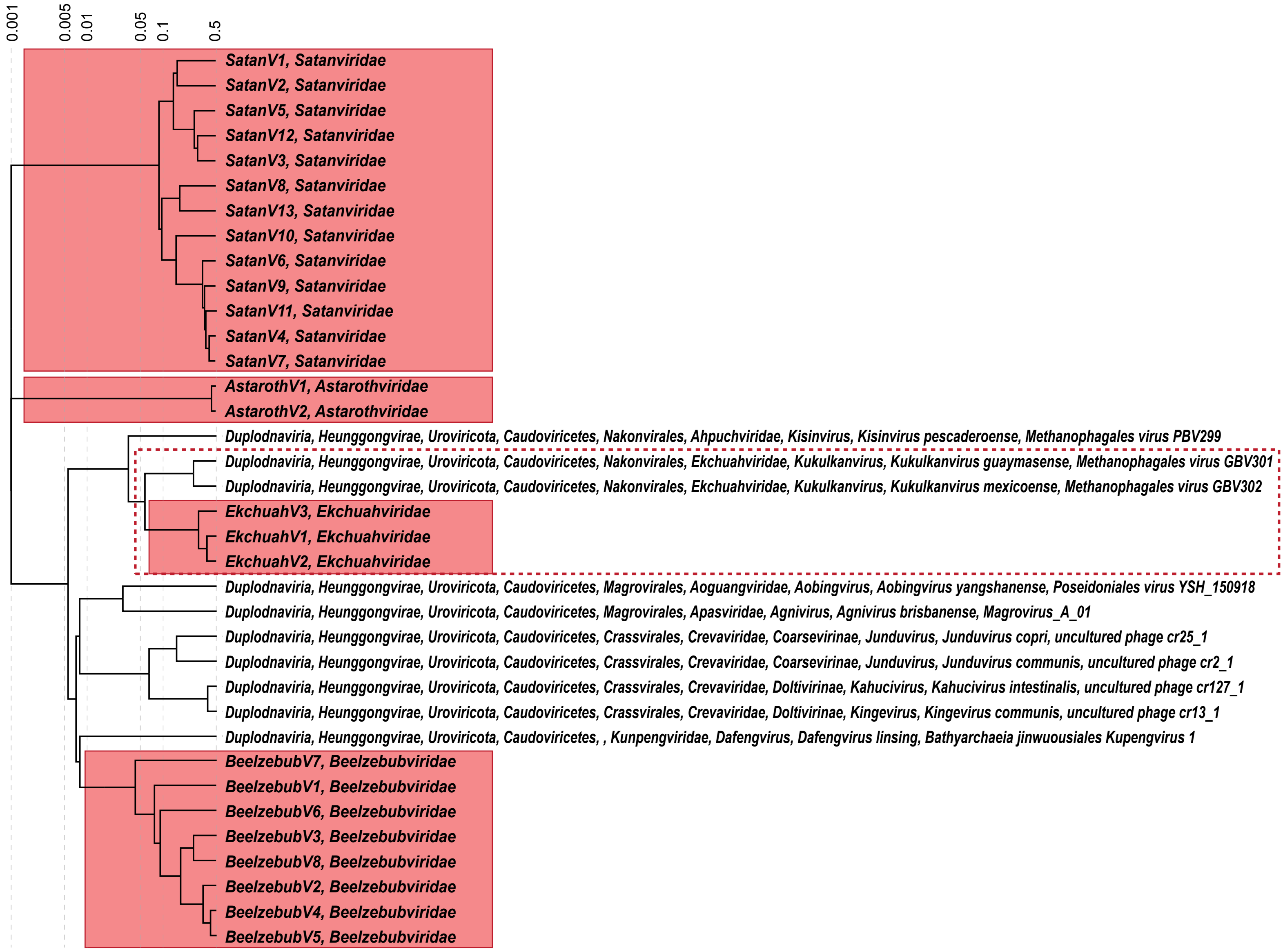

0.001
0.005
0.01
0.05
0.1
0.5
SatanV1, Satanviridae
SatanV2, Satanviridae
SatanV5, Satanviridae
SatanV12, Satanviridae
SatanV3, Satanviridae
SatanV8, Satanviridae
SatanV13, Satanviridae
SatanV10, Satanviridae
SatanV6, Satanviridae
SatanV9, Satanviridae
SatanV11, Satanviridae
SatanV4, Satanviridae
SatanV7, Satanviridae
AstarothV1, Astarothviridae
AstarothV2, Astarothviridae
Duplodnaviria, Heunggongvirae, Uroviricota, Caudoviricetes, Nakonvirales, Ahpuchviridae, Kisinvirus, Kisinvirus pescaderoense, Methanophagales virus PBV299
Duplodnaviria, Heunggongvirae, Uroviricota, Caudoviricetes, Nakonvirales, Ekchuahviridae, Kukulkanvirus, Kukulkanvirus guaymasense, Methanophagales virus GBV301
Duplodnaviria, Heunggongvirae, Uroviricota, Caudoviricetes, Nakonvirales, Ekchuahviridae, Kukulkanvirus, Kukulkanvirus mexicoense, Methanophagales virus GBV302
EkchuahV3, Ekchuahviridae
EkchuahV1, Ekchuahviridae
EkchuahV2, Ekchuahviridae
Duplodnaviria, Heunggongvirae, Uroviricota, Caudoviricetes, Magrovirales, Aoguangviridae, Aobingvirus, Aobingvirus yangshanense, Poseidoniales virus YSH_150918
Duplodnaviria, Heunggongvirae, Uroviricota, Caudoviricetes, Magrovirales, Apasviridae, Agnivirus, Agnivirus brisbanense, Magrovirus_A_01
Duplodnaviria, Heunggongvirae, Uroviricota, Caudoviricetes, Crassvirales, Crevaviridae, Coarsevirinae, Junduvirus, Junduvirus copri, uncultured phage cr25_1
Duplodnaviria, Heunggongvirae, Uroviricota, Caudoviricetes, Crassvirales, Crevaviridae, Coarsevirinae, Junduvirus, Junduvirus communis, uncultured phage cr2_1
Duplodnaviria, Heunggongvirae, Uroviricota, Caudoviricetes, Crassvirales, Crevaviridae, Doltivirinae, Kahucivirus, Kahucivirus intestinalis, uncultured phage cr127_1
Duplodnaviria, Heunggongvirae, Uroviricota, Caudoviricetes, Crassvirales, Crevaviridae, Doltivirinae, Kingevirus, Kingevirus communis, uncultured phage cr13_1
Duplodnaviria, Heunggongvirae, Uroviricota, Caudoviricetes, , Kunpengviridae, Dafengvirus, Dafengvirus linsing, Bathyarchaeia jinwuousiales Kupengvirus 1
BeelzebubV7, Beelzebubviridae
BeelzebubV1, Beelzebubviridae
BeelzebubV6, Beelzebubviridae
BeelzebubV3, Beelzebubviridae
BeelzebubV8, Beelzebubviridae
BeelzebubV2, Beelzebubviridae
BeelzebubV4, Beelzebubviridae
BeelzebubV5, Beelzebubviridae

Supplementary Fig. 4. A subset of the VipTree for head-tail viruses generated from all ICTV head-tail virus genomes, and all representative genomes from head-tail morphotypes identified in our study (highlighted in red). Newly identified sequences group into 4 clades, 3 of which diverge from known sequences beyond the accepted distance cutoff of 0.05, and so represent novel viral families. Three sequences branch close to previously published *Ekchuahviridae,* and are thus considered members of this family.

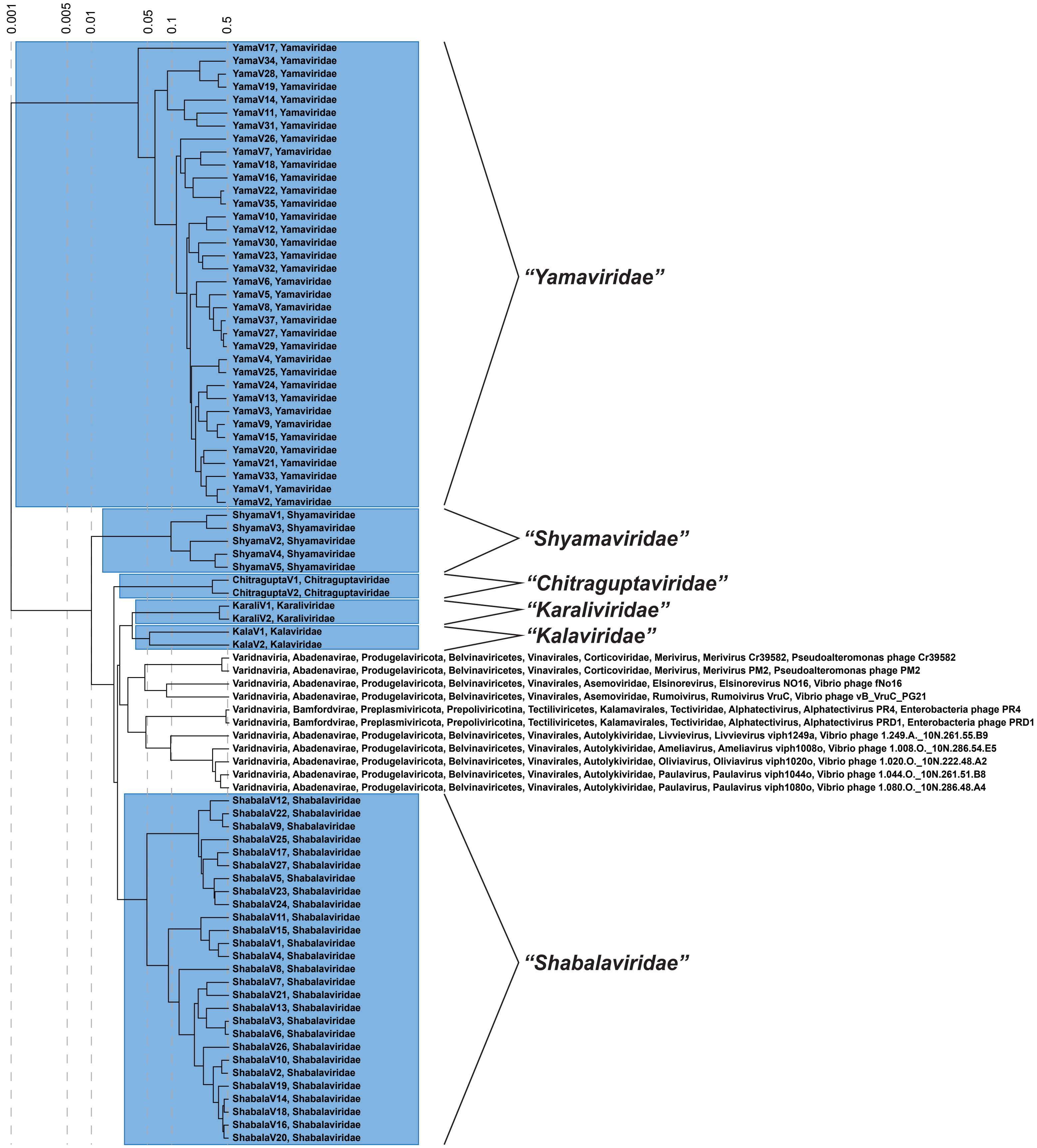

0.001
0.005
0.01
0.05
0.1
0.5
YamaV17, Yamaviridae
YamaV34, Yamaviridae
YamaV28, Yamaviridae
YamaV19, Yamaviridae
YamaV14, Yamaviridae
YamaV11, Yamaviridae
YamaV31, Yamaviridae
YamaV26, Yamaviridae
YamaV7, Yamaviridae
YamaV18, Yamaviridae
YamaV16, Yamaviridae
YamaV22, Yamaviridae
YamaV35, Yamaviridae
YamaV10, Yamaviridae
YamaV12, Yamaviridae
YamaV30, Yamaviridae
YamaV23, Yamaviridae
YamaV32, Yamaviridae
YamaV6, Yamaviridae
YamaV5, Yamaviridae
YamaV8, Yamaviridae
YamaV37, Yamaviridae
YamaV27, Yamaviridae
YamaV29, Yamaviridae
YamaV4, Yamaviridae
YamaV25, Yamaviridae
YamaV24, Yamaviridae
YamaV13, Yamaviridae
YamaV3, Yamaviridae
YamaV9, Yamaviridae
YamaV15, Yamaviridae
YamaV20, Yamaviridae
YamaV21, Yamaviridae
YamaV33, Yamaviridae
YamaV1, Yamaviridae
YamaV2, Yamaviridae
"Yamaviridae"
ShyamaV1, Shyamaviridae
ShyamaV3, Shyamaviridae
ShyamaV2, Shyamaviridae
ShyamaV4, Shyamaviridae
ShyamaV5, Shyamaviridae
"Shyamaviridae"
ChitraguptaV1, Chitraguptaviridae
ChitraguptaV2, Chitraguptaviridae
"Chitraguptaviridae"
KaraliV1, Karaliviridae
KaraliV2, Karaliviridae
"Karaliviridae"
KalaV1, Kalaviridae
KalaV2, Kalaviridae
"Kalaviridae"
Varidnaviria, Abadenavirae, Produgelaviricota, Belvinaviricetes, Vinavirales, Corticoviridae, Merivirus, Merivirus Cr39582, Pseudoalteromonas phage Cr39582
Varidnaviria, Abadenavirae, Produgelaviricota, Belvinaviricetes, Vinavirales, Corticoviridae, Merivirus, Merivirus PM2, Pseudoalteromonas phage PM2
Varidnaviria, Abadenavirae, Produgelaviricota, Belvinaviricetes, Vinavirales, Asemoviridae, Elsinorevirus, Elsinorevirus NO16, Vibrio phage fNo16
Varidnaviria, Abadenavirae, Produgelaviricota, Belvinaviricetes, Vinavirales, Asemoviridae, Rumoivirus, Rumoivirus VruC, Vibrio phage vB_VruC_PG21
Varidnaviria, Bamfordvirae, Preplasmiviricota, Prepoliviricotina, Tectiliviricetes, Kalamavirales, Tectiviridae, Alphatectivirus, Alphatectivirus PR4, Enterobacteria phage PR4
Varidnaviria, Bamfordvirae, Preplasmiviricota, Prepoliviricotina, Tectiliviricetes, Kalamavirales, Tectiviridae, Alphatectivirus, Alphatectivirus PRD1, Enterobacteria phage PRD1
Varidnaviria, Abadenavirae, Produgelaviricota, Belvinaviricetes, Vinavirales, Autolykiviridae, Livvievirus, Livvievirus viph1249a, Vibrio phage 1.249.A._10N.261.55.B9
Varidnaviria, Abadenavirae, Produgelaviricota, Belvinaviricetes, Vinavirales, Autolykiviridae, Ameliavirus, Ameliavirus viph1008o, Vibrio phage 1.008.O._10N.286.54.E5
Varidnaviria, Abadenavirae, Produgelaviricota, Belvinaviricetes, Vinavirales, Autolykiviridae, Oliviavirus, Oliviavirus viph1020o, Vibrio phage 1.020.O._10N.222.48.A2
Varidnaviria, Abadenavirae, Produgelaviricota, Belvinaviricetes, Vinavirales, Autolykiviridae, Paulavirus, Paulavirus viph1044o, Vibrio phage 1.044.O._10N.261.51.B8
Varidnaviria, Abadenavirae, Produgelaviricota, Belvinaviricetes, Vinavirales, Autolykiviridae, Paulavirus, Paulavirus viph1080o, Vibrio phage 1.080.O._10N.286.48.A4
ShabalaV12, Shabalaviridae
ShabalaV22, Shabalaviridae
ShabalaV9, Shabalaviridae
ShabalaV25, Shabalaviridae
ShabalaV17, Shabalaviridae
ShabalaV27, Shabalaviridae
ShabalaV5, Shabalaviridae
ShabalaV23, Shabalaviridae
ShabalaV24, Shabalaviridae
ShabalaV11, Shabalaviridae
ShabalaV15, Shabalaviridae
ShabalaV1, Shabalaviridae
ShabalaV4, Shabalaviridae
ShabalaV8, Shabalaviridae
ShabalaV7, Shabalaviridae
ShabalaV21, Shabalaviridae
ShabalaV13, Shabalaviridae
ShabalaV3, Shabalaviridae
ShabalaV6, Shabalaviridae
ShabalaV26, Shabalaviridae
ShabalaV10, Shabalaviridae
ShabalaV2, Shabalaviridae
ShabalaV19, Shabalaviridae
ShabalaV14, Shabalaviridae
ShabalaV18, Shabalaviridae
ShabalaV16, Shabalaviridae
ShabalaV20, Shabalaviridae
"Shabalaviridae"

Supplementary Fig. 5. A subset of the VipTree for icosahedral viruses generated from all ICTV icosahedral virus genomes, and all representative genomes from icosahedral morphotypes identified in our study (highlighted in blue) (note *"Dandaviridae"* are excluded from this analysis due to a lack of complete genomes). To be consistent with the taxonomy of previously published viruses, we selected a family-defining distance cutoff of ~0.03, thereby grouping our sequences into 6 new families of icosahedral viruses.

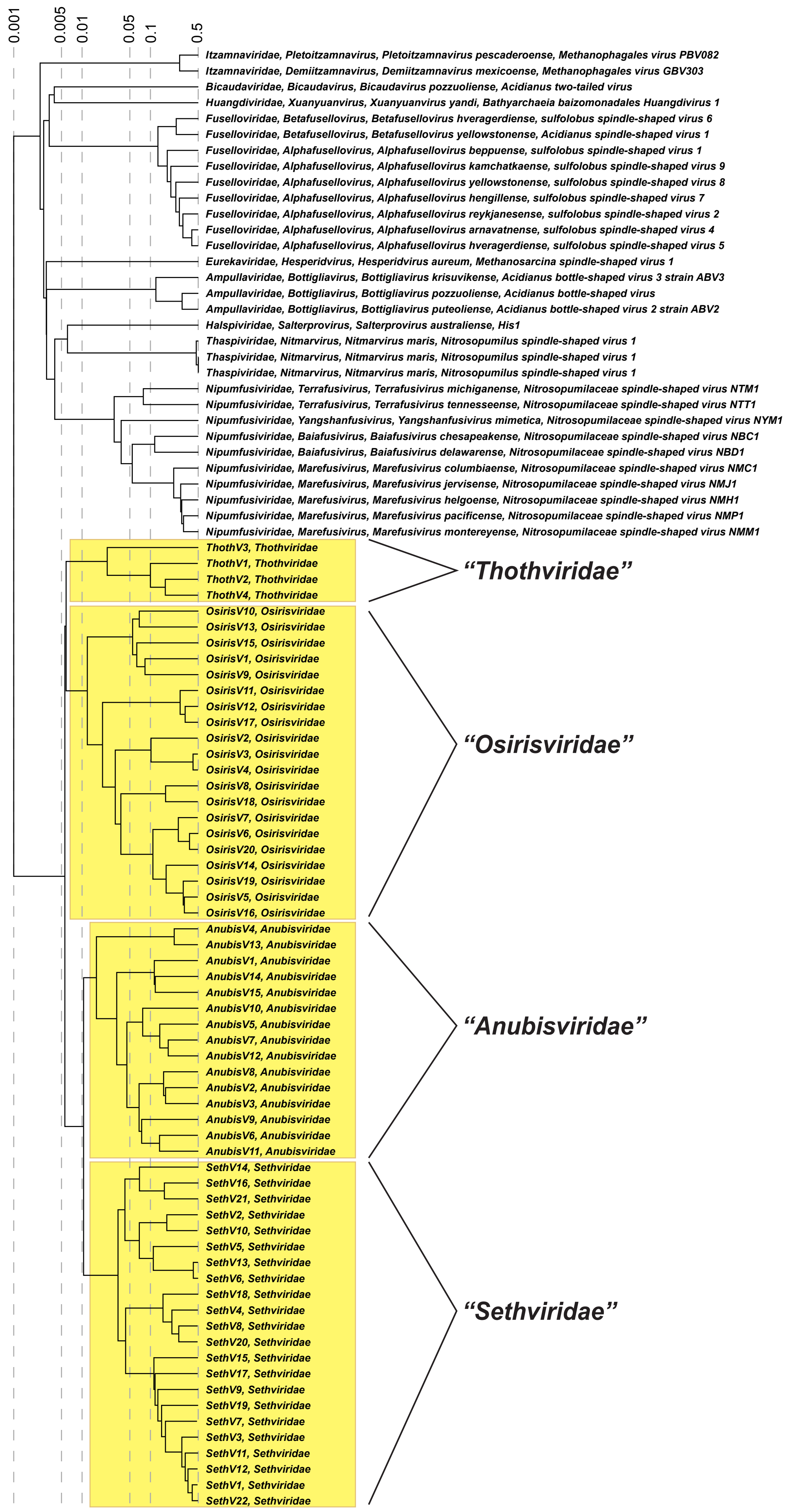
0.001
0.005
0.01
0.05
0.1
0.5
Itzamnaviridae, Pletoitzamnavirus, Pletoitzamnavirus pescaderoense, Methanophagales virus PBV082
Itzamnaviridae, Demiitzamnavirus, Demiitzamnavirus mexicoense, Methanophagales virus GBV303
Bicaudaviridae, Bicaudavirus, Bicaudavirus pozzuoliense, Acidianus two-tailed virus
Huangdiviridae, Xuanyuanvirus, Xuanyuanvirus yandi, Bathyarchaeia baizomonadales Huangdivirus 1
Fuselloviridae, Betafusellovirus, Betafusellovirus hveragerdiense, sulfolobus spindle-shaped virus 6
Fuselloviridae, Betafusellovirus, Betafusellovirus yellowstonense, Acidianus spindle-shaped virus 1
Fuselloviridae, Alphafusellovirus, Alphafusellovirus beppuense, sulfolobus spindle-shaped virus 1
Fuselloviridae, Alphafusellovirus, Alphafusellovirus kamchatkaense, sulfolobus spindle-shaped virus 9
Fuselloviridae, Alphafusellovirus, Alphafusellovirus yellowstonense, sulfolobus spindle-shaped virus 8
Fuselloviridae, Alphafusellovirus, Alphafusellovirus hengillense, sulfolobus spindle-shaped virus 7
Fuselloviridae, Alphafusellovirus, Alphafusellovirus reykjanesense, sulfolobus spindle-shaped virus 2
Fuselloviridae, Alphafusellovirus, Alphafusellovirus arnavatnense, sulfolobus spindle-shaped virus 4
Fuselloviridae, Alphafusellovirus, Alphafusellovirus hveragerdiense, sulfolobus spindle-shaped virus 5
Eurekaviridae, Hesperidvirus, Hesperidvirus aureum, Methanosarcina spindle-shaped virus 1
Ampullaviridae, Bottigliavirus, Bottigliavirus krisuvikense, Acidianus bottle-shaped virus 3 strain ABV3
Ampullaviridae, Bottigliavirus, Bottigliavirus pozzuoliense, Acidianus bottle-shaped virus
Ampullaviridae, Bottigliavirus, Bottigliavirus puteoliense, Acidianus bottle-shaped virus 2 strain ABV2
Halspiviridae, Salterprovirus, Salterprovirus australiense, His1
Thaspiviridae, Nitmarvirus, Nitmarvirus maris, Nitrosopumilus spindle-shaped virus 1
Thaspiviridae, Nitmarvirus, Nitmarvirus maris, Nitrosopumilus spindle-shaped virus 1
Thaspiviridae, Nitmarvirus, Nitmarvirus maris, Nitrosopumilus spindle-shaped virus 1
Nipumfusiviridae, Terrafusivirus, Terrafusivirus michiganense, Nitrosopumilaceae spindle-shaped virus NTM1
Nipumfusiviridae, Terrafusivirus, Terrafusivirus tennesseense, Nitrosopumilaceae spindle-shaped virus NTT1
Nipumfusiviridae, Yangshanfusivirus, Yangshanfusivirus mimetica, Nitrosopumilaceae spindle-shaped virus NYM1
Nipumfusiviridae, Baiafusivirus, Baiafusivirus chesapeakense, Nitrosopumilaceae spindle-shaped virus NBC1
Nipumfusiviridae, Baiafusivirus, Baiafusivirus delawarense, Nitrosopumilaceae spindle-shaped virus NBD1
Nipumfusiviridae, Marefusivirus, Marefusivirus columbiaense, Nitrosopumilaceae spindle-shaped virus NMC1
Nipumfusiviridae, Marefusivirus, Marefusivirus jervisense, Nitrosopumilaceae spindle-shaped virus NMJ1
Nipumfusiviridae, Marefusivirus, Marefusivirus helgoense, Nitrosopumilaceae spindle-shaped virus NMH1
Nipumfusiviridae, Marefusivirus, Marefusivirus pacificense, Nitrosopumilaceae spindle-shaped virus NMP1
Nipumfusiviridae, Marefusivirus, Marefusivirus montereyense, Nitrosopumilaceae spindle-shaped virus NMM1
ThothV3, Thothviridae
ThothV1, Thothviridae
ThothV2, Thothviridae
ThothV4, Thothviridae
“Thothviridae”
OsirisV10, Osirisviridae
OsirisV13, Osirisviridae
OsirisV15, Osirisviridae
OsirisV1, Osirisviridae
OsirisV9, Osirisviridae
OsirisV11, Osirisviridae
OsirisV12, Osirisviridae
OsirisV17, Osirisviridae
OsirisV2, Osirisviridae
OsirisV3, Osirisviridae
OsirisV4, Osirisviridae
OsirisV8, Osirisviridae
OsirisV18, Osirisviridae
OsirisV7, Osirisviridae
OsirisV6, Osirisviridae
OsirisV20, Osirisviridae
OsirisV14, Osirisviridae
OsirisV19, Osirisviridae
OsirisV5, Osirisviridae
OsirisV16, Osirisviridae
“Osirisviridae”
AnubisV4, Anubisviridae
AnubisV13, Anubisviridae
AnubisV1, Anubisviridae
AnubisV14, Anubisviridae
AnubisV15, Anubisviridae
AnubisV10, Anubisviridae
AnubisV5, Anubisviridae
AnubisV7, Anubisviridae
AnubisV12, Anubisviridae
AnubisV8, Anubisviridae
AnubisV2, Anubisviridae
AnubisV3, Anubisviridae
AnubisV9, Anubisviridae
AnubisV6, Anubisviridae
AnubisV11, Anubisviridae
“Anubisviridae”
SethV14, Sethviridae
SethV16, Sethviridae
SethV21, Sethviridae
SethV2, Sethviridae
SethV10, Sethviridae
SethV5, Sethviridae
SethV13, Sethviridae
SethV6, Sethviridae
SethV18, Sethviridae
SethV4, Sethviridae
SethV8, Sethviridae
SethV20, Sethviridae
SethV15, Sethviridae
SethV17, Sethviridae
SethV9, Sethviridae
SethV19, Sethviridae
SethV7, Sethviridae
SethV3, Sethviridae
SethV11, Sethviridae
SethV12, Sethviridae
SethV1, Sethviridae
SethV22, Sethviridae
“Sethviridae”

Supplementary Fig. 6. A VipTree of spindle-shaped viruses generated from all ICTV spindle-shaped virus genomes and all representative genomes from spindle-shaped morphotypes identified in our study (highlighted in yellow). Taxonomy of published genomes is somewhat inconsistent, but a distance cutoff of 0.01 maintains most family divisions. At this distance, we define 4 new families of Thermococcales-associated spindle-shaped viruses.

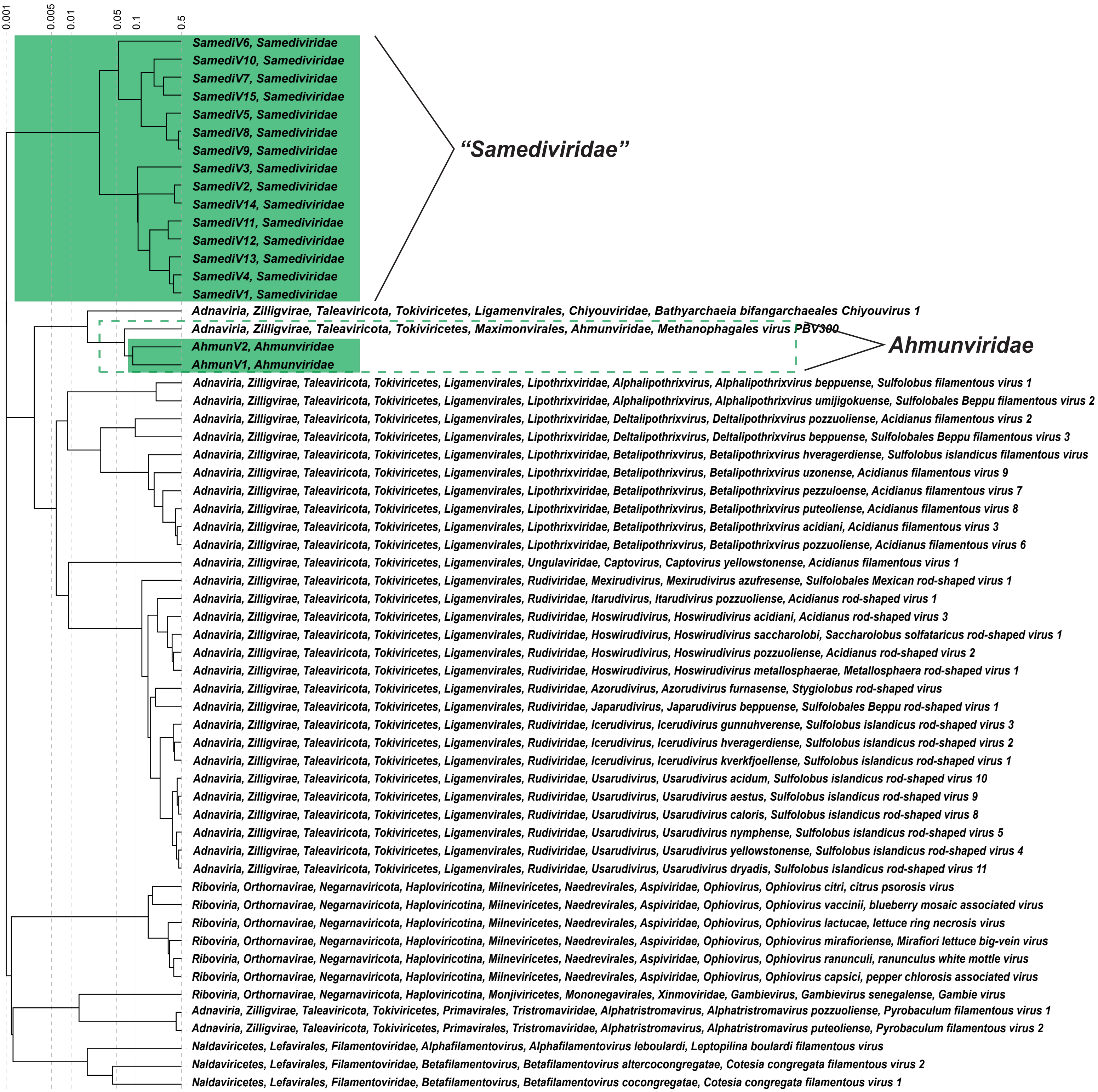
0.001
0.005
0.01
0.05
0.1
0.5
SamediV6, Samediviridae
SamediV10, Samediviridae
SamediV7, Samediviridae
SamediV15, Samediviridae
SamediV5, Samediviridae
SamediV8, Samediviridae
SamediV9, Samediviridae
SamediV3, Samediviridae
SamediV2, Samediviridae
SamediV14, Samediviridae
SamediV11, Samediviridae
SamediV12, Samediviridae
SamediV13, Samediviridae
SamediV4, Samediviridae
SamediV1, Samediviridae
“Samediviridae”
Adnaviria, Zilligvirae, Taleaviricota, Tokiviricetes, Ligamenvirales, Chiyouviridae, Bathyarchaeia bifangarchaeales Chiyouvirus 1
Adnaviria, Zilligvirae, Taleaviricota, Tokiviricetes, Maximonvirales, Ahmunviridae, Methanophagales virus PBV300
AhmunV2, Ahmunviridae
AhmunV1, Ahmunviridae
Ahmunviridae
Adnaviria, Zilligvirae, Taleaviricota, Tokiviricetes, Ligamenvirales, Lipothrixviridae, Alphalipothrixvirus, Alphalipothrixvirus beppuense, Sulfolobus filamentous virus 1
Adnaviria, Zilligvirae, Taleaviricota, Tokiviricetes, Ligamenvirales, Lipothrixviridae, Alphalipothrixvirus, Alphalipothrixvirus umijigokuense, Sulfolobales Beppu filamentous virus 2
Adnaviria, Zilligvirae, Taleaviricota, Tokiviricetes, Ligamenvirales, Lipothrixviridae, Deltalipothrixvirus, Deltalipothrixvirus pozzuoliense, Acidianus filamentous virus 2
Adnaviria, Zilligvirae, Taleaviricota, Tokiviricetes, Ligamenvirales, Lipothrixviridae, Deltalipothrixvirus, Deltalipothrixvirus beppuense, Sulfolobales Beppu filamentous virus 3
Adnaviria, Zilligvirae, Taleaviricota, Tokiviricetes, Ligamenvirales, Lipothrixviridae, Betalipothrixvirus, Betalipothrixvirus hveragerdiense, Sulfolobus islandicus filamentous virus
Adnaviria, Zilligvirae, Taleaviricota, Tokiviricetes, Ligamenvirales, Lipothrixviridae, Betalipothrixvirus, Betalipothrixvirus uzonense, Acidianus filamentous virus 9
Adnaviria, Zilligvirae, Taleaviricota, Tokiviricetes, Ligamenvirales, Lipothrixviridae, Betalipothrixvirus, Betalipothrixvirus pezzuloense, Acidianus filamentous virus 7
Adnaviria, Zilligvirae, Taleaviricota, Tokiviricetes, Ligamenvirales, Lipothrixviridae, Betalipothrixvirus, Betalipothrixvirus puteoliense, Acidianus filamentous virus 8
Adnaviria, Zilligvirae, Taleaviricota, Tokiviricetes, Ligamenvirales, Lipothrixviridae, Betalipothrixvirus, Betalipothrixvirus acidiani, Acidianus filamentous virus 3
Adnaviria, Zilligvirae, Taleaviricota, Tokiviricetes, Ligamenvirales, Lipothrixviridae, Betalipothrixvirus, Betalipothrixvirus pozzuoliense, Acidianus filamentous virus 6
Adnaviria, Zilligvirae, Taleaviricota, Tokiviricetes, Ligamenvirales, Ungulaviridae, Captovirus, Captovirus yellowstonense, Acidianus filamentous virus 1
Adnaviria, Zilligvirae, Taleaviricota, Tokiviricetes, Ligamenvirales, Rudiviridae, Mexirudivirus, Mexirudivirus azufresense, Sulfolobales Mexican rod-shaped virus 1
Adnaviria, Zilligvirae, Taleaviricota, Tokiviricetes, Ligamenvirales, Rudiviridae, Itarudivirus, Itarudivirus pozzuoliense, Acidianus rod-shaped virus 1
Adnaviria, Zilligvirae, Taleaviricota, Tokiviricetes, Ligamenvirales, Rudiviridae, Hoswirudivirus, Hoswirudivirus acidiani, Acidianus rod-shaped virus 3
Adnaviria, Zilligvirae, Taleaviricota, Tokiviricetes, Ligamenvirales, Rudiviridae, Hoswirudivirus, Hoswirudivirus saccharolobi, Saccharolobus solfataricus rod-shaped virus 1
Adnaviria, Zilligvirae, Taleaviricota, Tokiviricetes, Ligamenvirales, Rudiviridae, Hoswirudivirus, Hoswirudivirus pozzuoliense, Acidianus rod-shaped virus 2
Adnaviria, Zilligvirae, Taleaviricota, Tokiviricetes, Ligamenvirales, Rudiviridae, Hoswirudivirus, Hoswirudivirus metallosphaerae, Metallosphaera rod-shaped virus 1
Adnaviria, Zilligvirae, Taleaviricota, Tokiviricetes, Ligamenvirales, Rudiviridae, Azorudivirus, Azorudivirus furnasense, Stygiolobus rod-shaped virus
Adnaviria, Zilligvirae, Taleaviricota, Tokiviricetes, Ligamenvirales, Rudiviridae, Japarudivirus, Japarudivirus beppuense, Sulfolobales Beppu rod-shaped virus 1
Adnaviria, Zilligvirae, Taleaviricota, Tokiviricetes, Ligamenvirales, Rudiviridae, Icerudivirus, Icerudivirus gunnuhverense, Sulfolobus islandicus rod-shaped virus 3
Adnaviria, Zilligvirae, Taleaviricota, Tokiviricetes, Ligamenvirales, Rudiviridae, Icerudivirus, Icerudivirus hveragerdiense, Sulfolobus islandicus rod-shaped virus 2
Adnaviria, Zilligvirae, Taleaviricota, Tokiviricetes, Ligamenvirales, Rudiviridae, Icerudivirus, Icerudivirus kverkfjoellense, Sulfolobus islandicus rod-shaped virus 1
Adnaviria, Zilligvirae, Taleaviricota, Tokiviricetes, Ligamenvirales, Rudiviridae, Usarudivirus, Usarudivirus acidum, Sulfolobus islandicus rod-shaped virus 10
Adnaviria, Zilligvirae, Taleaviricota, Tokiviricetes, Ligamenvirales, Rudiviridae, Usarudivirus, Usarudivirus aestus, Sulfolobus islandicus rod-shaped virus 9
Adnaviria, Zilligvirae, Taleaviricota, Tokiviricetes, Ligamenvirales, Rudiviridae, Usarudivirus, Usarudivirus caloris, Sulfolobus islandicus rod-shaped virus 8
Adnaviria, Zilligvirae, Taleaviricota, Tokiviricetes, Ligamenvirales, Rudiviridae, Usarudivirus, Usarudivirus nymphense, Sulfolobus islandicus rod-shaped virus 5
Adnaviria, Zilligvirae, Taleaviricota, Tokiviricetes, Ligamenvirales, Rudiviridae, Usarudivirus, Usarudivirus yellowstonense, Sulfolobus islandicus rod-shaped virus 4
Adnaviria, Zilligvirae, Taleaviricota, Tokiviricetes, Ligamenvirales, Rudiviridae, Usarudivirus, Usarudivirus dryadis, Sulfolobus islandicus rod-shaped virus 11
Riboviria, Orthornavirae, Negarnaviricota, Haploviricotina, Milneviricetes, Naedrevirales, Aspiviridae, Ophiovirus, Ophiovirus citri, citrus psorosis virus
Riboviria, Orthornavirae, Negarnaviricota, Haploviricotina, Milneviricetes, Naedrevirales, Aspiviridae, Ophiovirus, Ophiovirus vaccinii, blueberry mosaic associated virus
Riboviria, Orthornavirae, Negarnaviricota, Haploviricotina, Milneviricetes, Naedrevirales, Aspiviridae, Ophiovirus, Ophiovirus lactucae, lettuce ring necrosis virus
Riboviria, Orthornavirae, Negarnaviricota, Haploviricotina, Milneviricetes, Naedrevirales, Aspiviridae, Ophiovirus, Ophiovirus mirafioriense, Mirafiori lettuce big-vein virus
Riboviria, Orthornavirae, Negarnaviricota, Haploviricotina, Milneviricetes, Naedrevirales, Aspiviridae, Ophiovirus, Ophiovirus ranunculi, ranunculus white mottle virus
Riboviria, Orthornavirae, Negarnaviricota, Haploviricotina, Milneviricetes, Naedrevirales, Aspiviridae, Ophiovirus, Ophiovirus capsici, pepper chlorosis associated virus
Riboviria, Orthornavirae, Negarnaviricota, Haploviricotina, Monjiviricetes, Mononegavirales, Xinmoviridae, Gambievirus, Gambievirus senegalense, Gambie virus
Adnaviria, Zilligvirae, Taleaviricota, Tokiviricetes, Primavirales, Tristromaviridae, Alphatristromavirus, Alphatristromavirus pozzuoliense, Pyrobaculum filamentous virus 1
Adnaviria, Zilligvirae, Taleaviricota, Tokiviricetes, Primavirales, Tristromaviridae, Alphatristromavirus, Alphatristromavirus puteoliense, Pyrobaculum filamentous virus 2
Naldaviricetes, Lefavirales, Filamentoviridae, Alphafilamentovirus, Alphafilamentovirus leboulardi, Leptopilina boulardi filamentous virus
Naldaviricetes, Lefavirales, Filamentoviridae, Betafilamentovirus, Betafilamentovirus altercocongregatae, Cotesia congregata filamentous virus 2
Naldaviricetes, Lefavirales, Filamentoviridae, Betafilamentovirus, Betafilamentovirus cocongregatae, Cotesia congregata filamentous virus 1

Supplementary Fig. 7. A subset of the VipTree for filamentous viruses generated from all ICTV filamentous virus genomes, and all representative genomes from filamentous morphotypes identified in our study (highlighted in green). *"Samediviridae"* are highly divergent relative to all other described genomes, and thus confidently describe a new family. Two other new genomes are closely related to previously published *Ahmunviridae*, and thus expand this viral family.

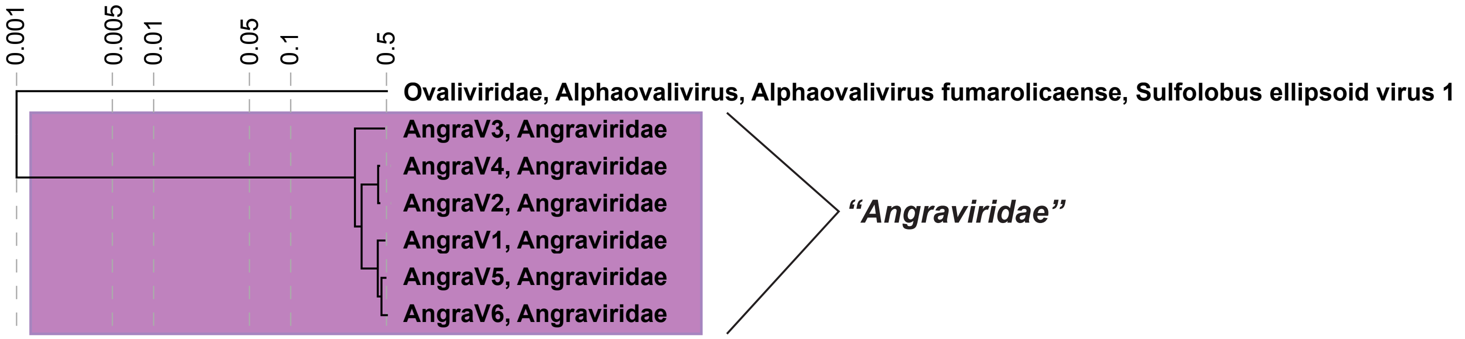
0.001
0.005
0.01
0.05
0.1
0.5
Ovaliviridae, Alphaovalivirus, Alphaovalivirus fumarolicaense, Sulfolobus ellipsoid virus 1
AngraV3, Angraviridae
AngraV4, Angraviridae
AngraV2, Angraviridae
AngraV1, Angraviridae
AngraV5, Angraviridae
AngraV6, Angraviridae
"Angraviridae"

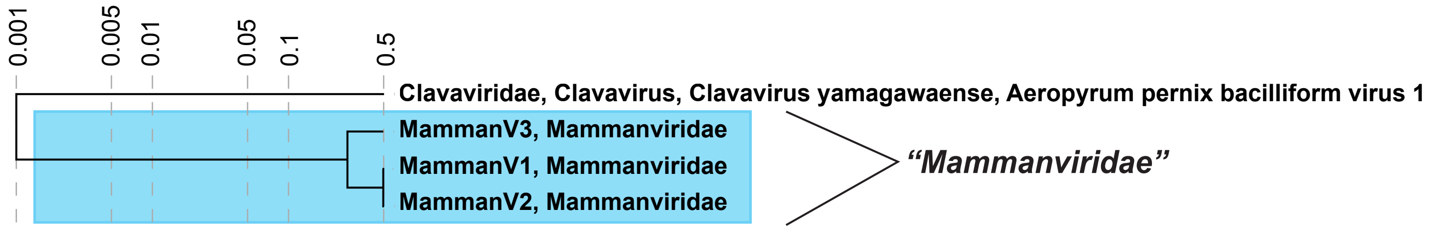
0.001
0.005
0.01
0.05
0.1
0.5
Clavaviridae, Clavavirus, Clavavirus yamagawaense, Aeropyrum pernix bacilliform virus 1
MammanV3, Mammanviridae
MammanV1, Mammanviridae
MammanV2, Mammanviridae
"Mammanviridae"

Supplementary Fig. 8. VipTrees for oval-shaped and bacilliform viruses identified in our study (highlighted in purple and cyan, respectively), including the single published representatives of each morphotype. In each case, the new genomes are divergent from published sequences and thus define new families.

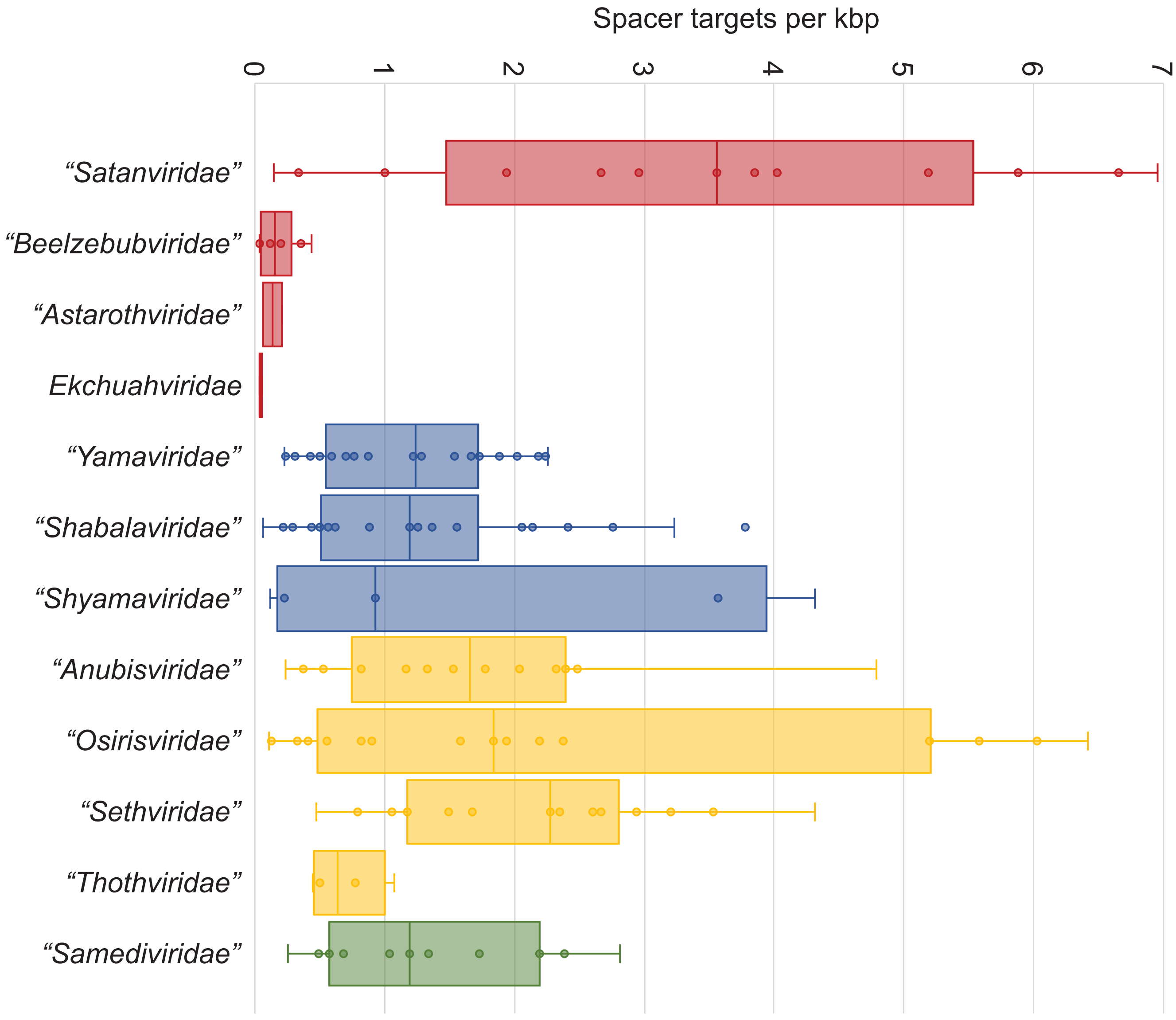

Spacer targets per kbp
0
1
2
3
4
5
6
7
"Satanviridae"
"Beelzebubviridae"
"Astarothviridae"
Ekchuahviridae
"Yamaviridae"
"Shabalaviridae"
"Shyamaviridae"
"Anubisviridae"
"Osirisviridae"
"Sethviridae"
"Thothviridae"
"Samediviridae"

Supplementary Fig. 9. Box and whisker plots showing density of spacer targets per kbp for our novel viral families. Each representative genome is indicated by a dot. Plots are colored based on predicted virion morphology (Fig. 2).

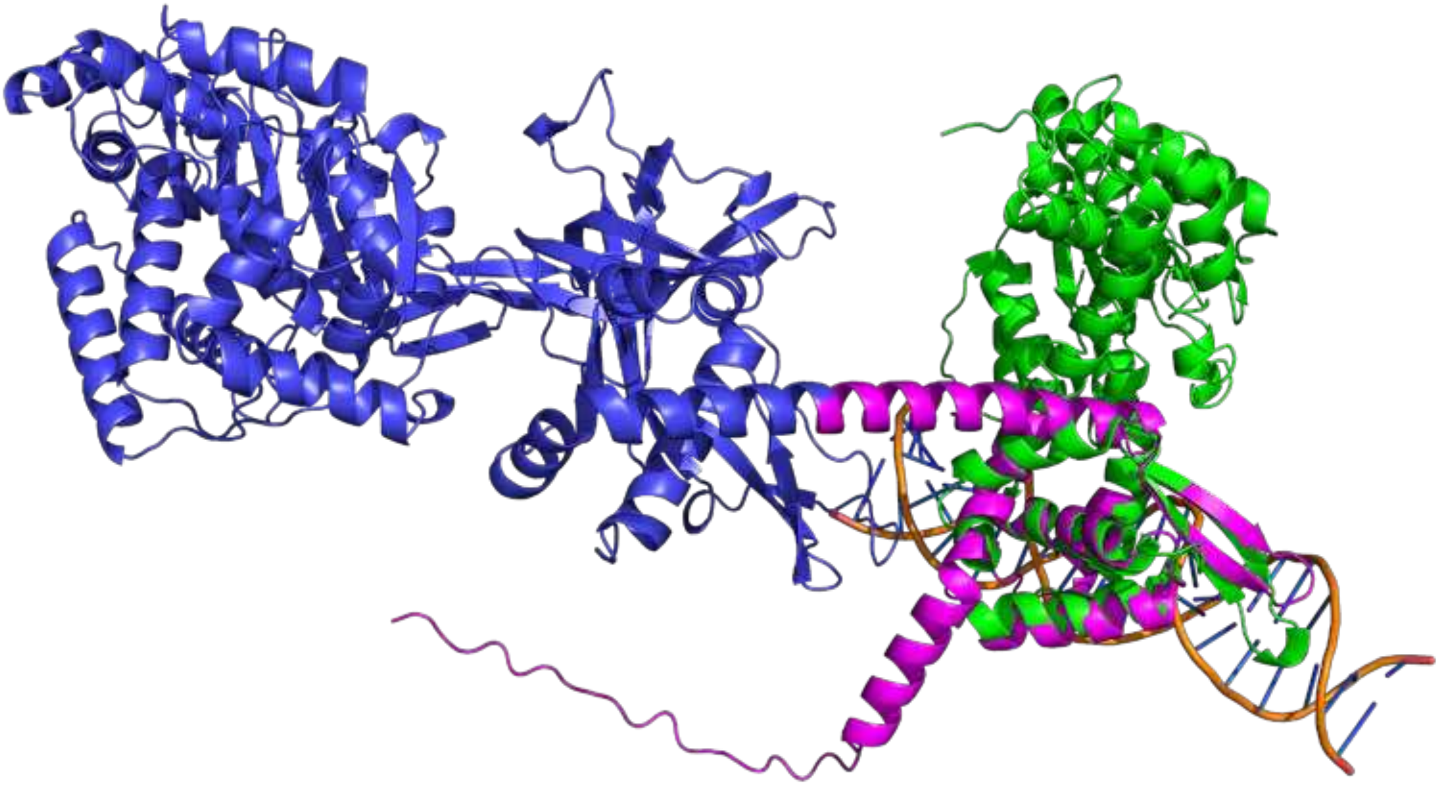

Supplementary Fig. 10. Structural alignment of a single subunit of "*Yamaviridae"* MCM Alphafold3 prediction (blue, with N-terminal extension in pink), with DNA-bound Orc1 (2V1U) from *Aeropyrum pernix* (green). The C-terminal domain of Orc1, responsible for binding the DNA origin of replication, structurally superimposes with the N-terminal domain of "*Yamaviridae"* MCM.

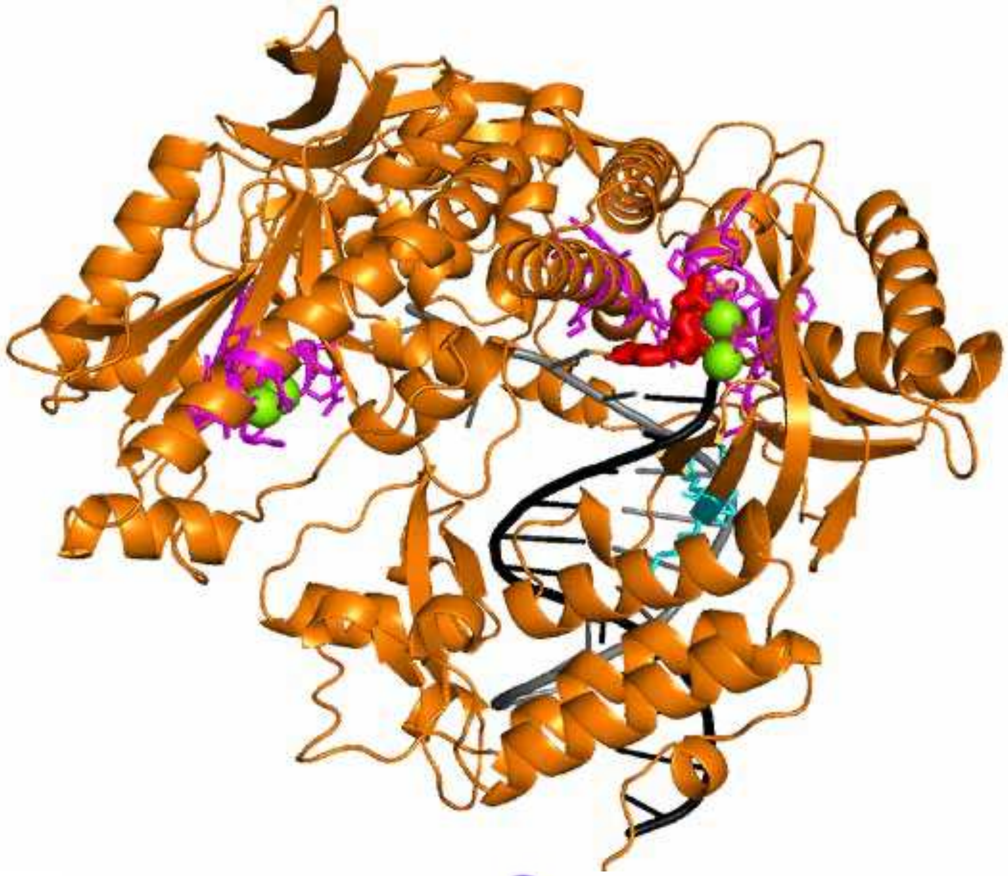

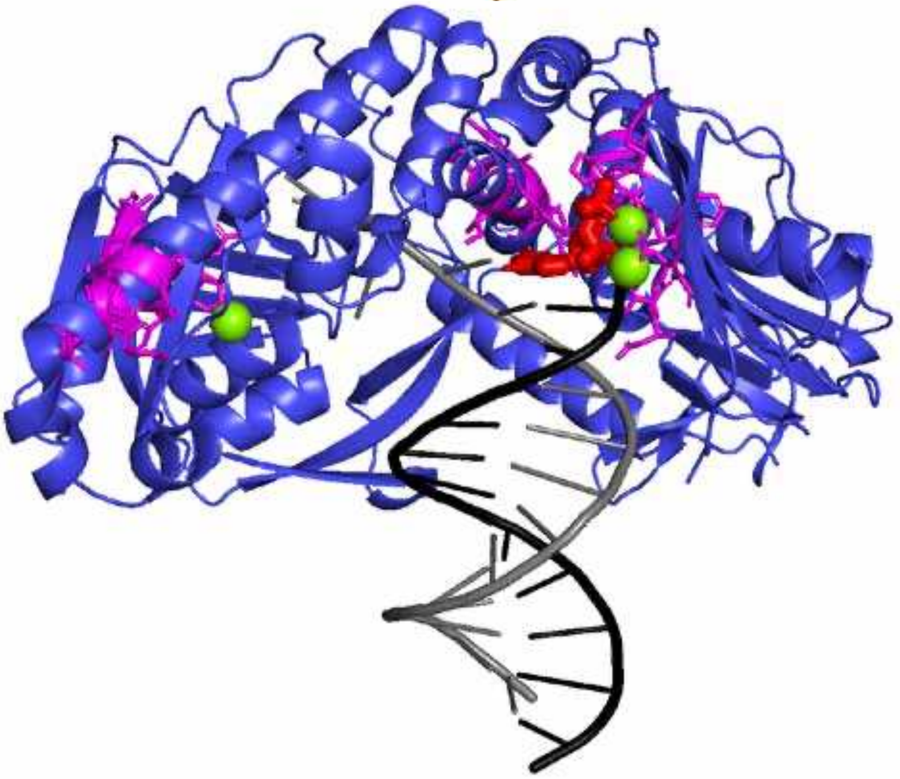

Supplementary Fig. 11. PolB from *T. kodakarensis* (orange, 774aa), and "*Kalaviridae*" (blue, 515aa). Protein structure modelled with template DNA strand (grey), extending DNA strand (black), GTP (red), Mg^{2+} (green). Conserved motifs indicated in Figure 4B are shown in magenta with atoms indicated by sticks. Exonuclease domain is on the left with magenta motifs and Mg^{2+} ions; the polymerase catalytic site is on the right. GTP can be seen base-pairing with cytosine of template DNA strand positioned for phosphodiester bond formation, with triphosphate stabilized by Mg^{2+} ions. For *T. kodakarensis* PolB, the C-terminal motif thought to stabilize DNA in the active site is shown in cyan (absent from "*Kalaviridae"* PolB).

SUPPLEMENTARY TEXT

"CRISPR spacers reveal diverse and abundant Thermococcales viruses in hydrothermal vents"

SUPPLEMENTARY TEXT

SECTION 1: Spindle-shaped viruses of Thermococcales

Protein clustering analysis and a genome-distance-based phylogenomic tree support four taxonomically distinct families of spindle-shaped viruses (Supplementary Fig. 6), all encoding homologs of major capsid proteins (MCPs) found in previously described exemplars. The only cultured Thermococcales viruses, PAV1 [1] and TPV1 [2], serve as the founding members of two of these families, while the remaining two are distinct from all known examples. We assigned these the names "*Osirisviridae*," "*Sethviridae*," "*Anubisviridae*," and "*Thothviridae*" after Osiris, Seth, Anubis, and Thoth, members of the Council of Osiris that oversee judgment of the dead in Egyptian mythology.

SECTION 1A: *"OSIRISVIRIDAE"*

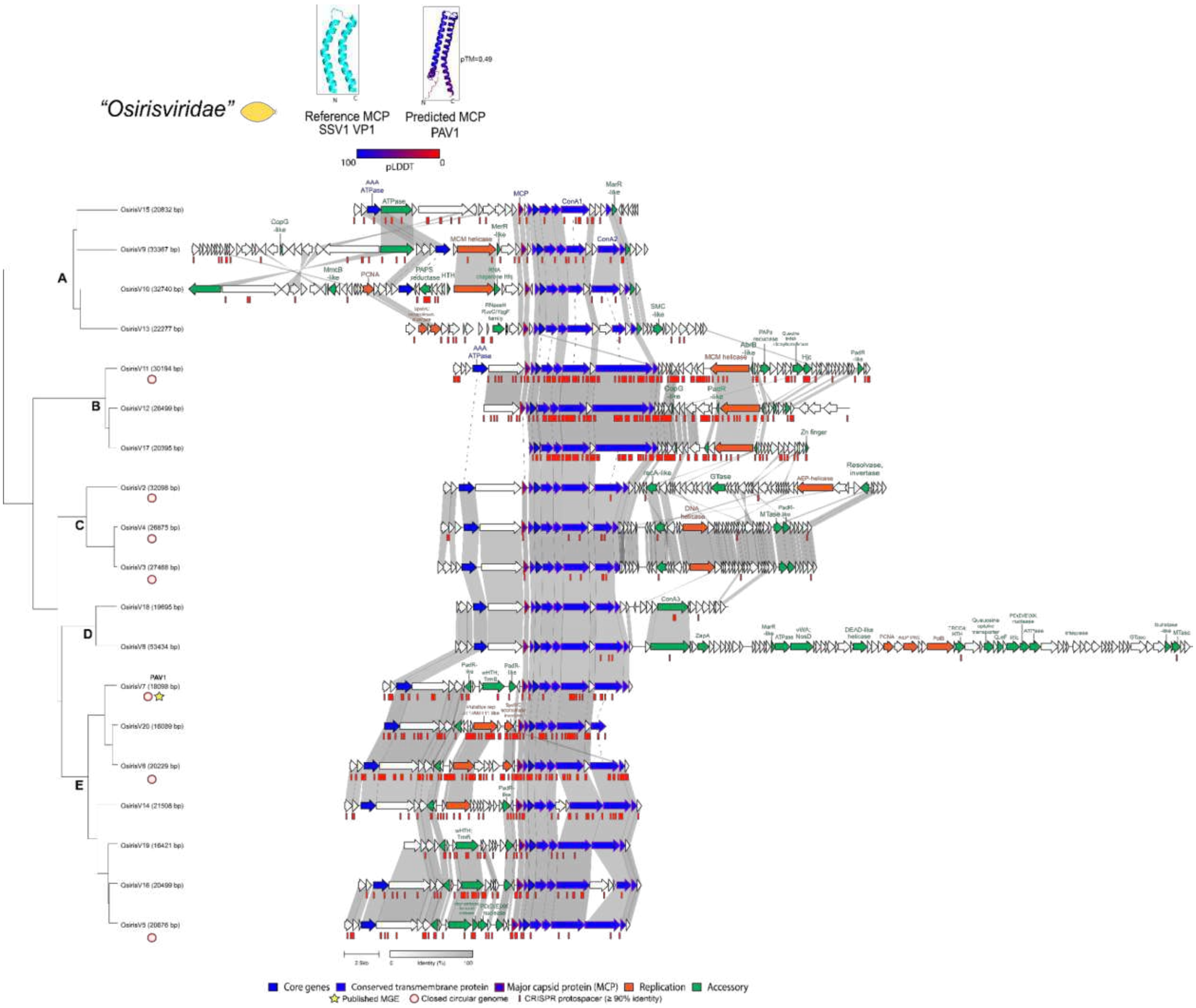

Supplementary Text Figure S1A. Genome synteny maps of representative osirisviruses. ViPTree proteomic phylogeny (left) supports five distinct clades (A-E). Published family members are marked by gold stars, and complete circular genomes are indicated by red circles. Predicted genes (coding sequences) are represented by arrows. Core genes (blue) are encoded by all complete or near-complete family members. Core genes predicted to encode major capsid proteins (MCPs) and transmembrane proteins are additionally distinguished by red and magenta borders, respectively. Replication-associated genes (orange) and accessory genes with high-

confidence functional predictions (green) are labelled, where applicable. Non-core genes of unknown function are coloured white. Homologous genes are connected by bars shaded in grayscale according to percent amino acid identity. Functionally conserved genes with sequence similarity below the similarity group threshold (Methods), or that are unconnected due to vertical ordering of the genomes, are instead connected by dashed lines. Protospacers matching published or predicted Thermococcales CRISPR spacers are indicated by vertical red rectangles. Top: AlphaFold3 structure of a representative osirisvirus MCP (with pTM score and coloured by PLDDT, blue to red) compared to the MCP VP1 of Sulfolobus spindle-shaped virus 1 (SSV1). N-termini and C-termini are denoted by N and C, respectively.

Osirisviruses include PAV1 and its previously undescribed relatives. The first described member of this clade is PAV1, identified in *Pyrococcus abyssi* GE23 and shown to produce spindle-shaped virions [1]. The genome of PAV1 appears to replicate as a stable episome, generating a lysogen-like or chronic infection state without integrating into the genome-consistent with the absence of a detectable integrase.

We expand the PAV1-associated group into a newly proposed viral family, “*Osirisviridae*”, comprising five distinct clades (Osiris A-E), represented by eight complete (circular) and eleven near-complete genomes, ranging from 18-35.6 kb in length. Core features of this family include: ORFs with significant similarity to PAV1’s uncharacterized integral membrane proteins, homologous predicted MCP, AAA ATPase, and two Concanavalin A lectin/glucanase domain-containing proteins (ConA1 and ConA2). AlphaFold3-predicted structures of these ConA proteins suggest a series of jellyroll (JR) domains connected by flexible linker regions and ending in a C-terminal bundle of transmembrane helices. ConA1 adopts a highly conserved topology of four JR domains, while ConA2 is structurally variable, suggesting modularity in glycan binding (see main text and Fig. 5 for further detail). The genome architecture of osirisviruses is otherwise highly variable across clades.

The canonical PAV1 clade (16-20.6 kb; 45.6-49.6% G+C) includes six highly similar genomes that share >80% genome-wide sequence similarity, diverging primarily within a hypervariable region bounded by PadR-like regulators of SpoIVC family recombinases. Variations include: OsirisV5 (20,676 bp) encodes a PD(D/E)XK nuclease and adjacent GlnB-like rhomboid intramembrane protease; OsirisV6 (20,229 bp) encodes a Rep protein (similar to that of plasmid pRT1) [3], and a predicted serine recombinase, indicating integration potential contrasting with PAV1; OsirisV10 (32,740 bp) is more divergent within this region, encoding an MCM helicase, phosphoadenosine phosphosulfate (PAPS) reductase, and multiple unknown ORFs.

Other viral clades lack this intervening region entirely, and the remainder of the genome downstream of the core transmembrane module shows remarkable diversity, encoding an array of predicted enzymes (methyltransferases, glycosyltransferases, additional ATPases, etc.). Other putative lifestyle markers are similarly variable outside of the clade containing the canonical PAV1, with several genomes encoding XerC/D-like integrases of the tyrosine recombinase superfamily and predicted replication machinery that can include either an MCM helicase,

archaeo-eukaryotic primase fused to a helicase domain (AEP-helicase), family B DNA polymerase (PolB), or PCNA, depending on the specific family member (see main text for further discussion of replication modules).

SECTION 1B: *"SETHVIRIDAE"*

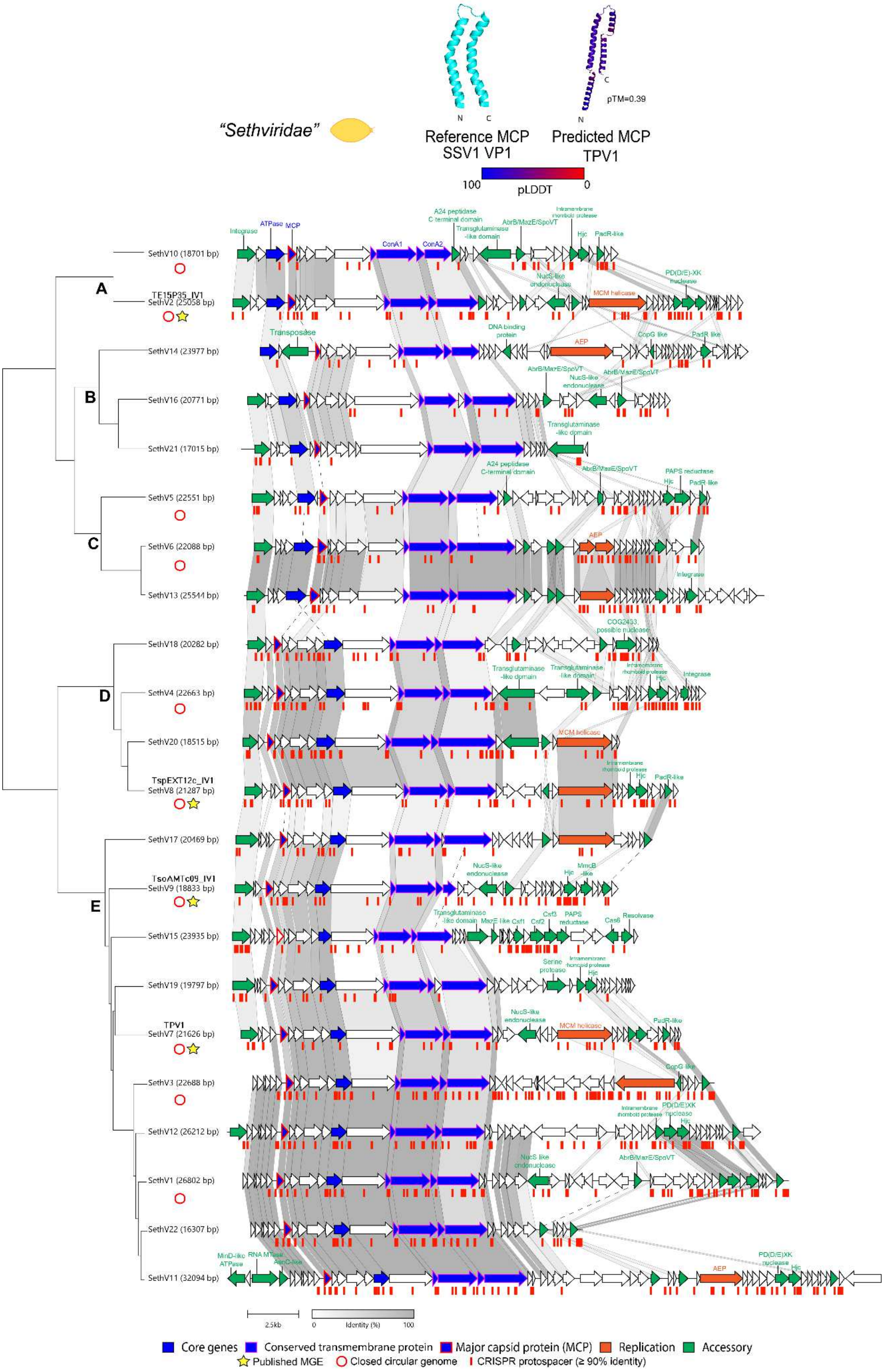

Supplementary Text Figure S1B. Genome synteny maps of representative sethviruses. ViPTree proteomic phylogeny (left) supports five distinct clades (A-E). Published family members are marked by gold stars, and complete circular genomes are indicated by red circles. Predicted genes (coding sequences) are represented by arrows. Core genes (blue) are encoded by all complete or near-complete family members. Core genes predicted to encode major capsid proteins (MCPs) and transmembrane proteins are additionally distinguished by red and magenta borders, respectively. Replication-associated genes (orange) and accessory genes with high-confidence functional predictions (green) are labelled, where applicable. Non-core genes of unknown function are coloured white. Homologous genes are connected by bars shaded in grayscale according to percent amino acid identity. Functionally conserved genes with sequence similarity below the similarity group threshold (Methods), or that are unconnected due to vertical ordering of the genomes, are instead connected by dashed lines. Protospacers matching published or predicted Thermococcales CRISPR spacers are indicated by vertical red rectangles. Top: AlphaFold3 structure of a representative sethvirus MCP (with pTM score and coloured by PLDDT, blue to red) compared to the MCP VP1 of Sulfolobus spindle-shaped virus 1 (SSV1). N-termini and C-termini are denoted by N and C, respectively.

Sethviruses, including TPV1 and its newly identified relatives, form a well-defined group of 22 members (10 complete and 12 near-complete genomes; 18.7-26.8 kb; 45-50% GC content). TPV1 was first isolated from *Thermococcus prieurii* and produces spindle-shaped virions [2]. Unlike PAV1, TPV1 encodes an integrase gene and is found in both integrated and episomal states. This dual lifestyle is reflected across the family, with 17/22 genomes containing tyrosine recombinase integrases, while two complete genomes lack them, suggesting obligate episomal replication.

All complete sethvirus genomes encode an AAA ATPase, a conserved cassette of predicted transmembrane proteins including the MCP, and two ConA-like proteins with similar JR+transmembrane architectures as *“Osirisviridae”.* ConA1 consistently encodes five JR domains; ConA2 is variable (2-6 JRs) (see Fig. 5 and main text for further detail).

Family members with complete genomes invariably encode a large ORF near ConA1 with weak or partial homology to SMC3 (Structural Maintenance of Chromosomes 3) [4]. Alphafold3 predicts largely disordered structures for these proteins so annotation is challenging.

In the hypervariable region, additional conserved genes include a Holliday junction resolvase (Hjc;15/22). A GlnB-like rhomboid protease is often directly adjacent to the Hjc or separated from the Hjc by a PD(D/E)XK nuclease. The hypervariable region encodes various predicted enzymes (e.g. peptidases, transglutaminases), probable transcriptional regulators (e.g., SpoVT-like, PadR-like), and many small ORFs of unknown function. Of particular interest, SethV15, encodes a type IV-B CRISPR-Cas system including a Cas6 gene, but no CRISPR array. This is the second reported case of this CRISPR-Cas subtype in an archaeal virus [5]. DNA replication modules are consistently positioned near the midpoint of the hypervariable region and appear to include MCM helicases, AEPs or AEP-helicase fusions. A subset of *Sethviridae* lacks identifiable replicase genes (see main text for further discussion).

SECTION 1C: *THOTHVIRIDAE*

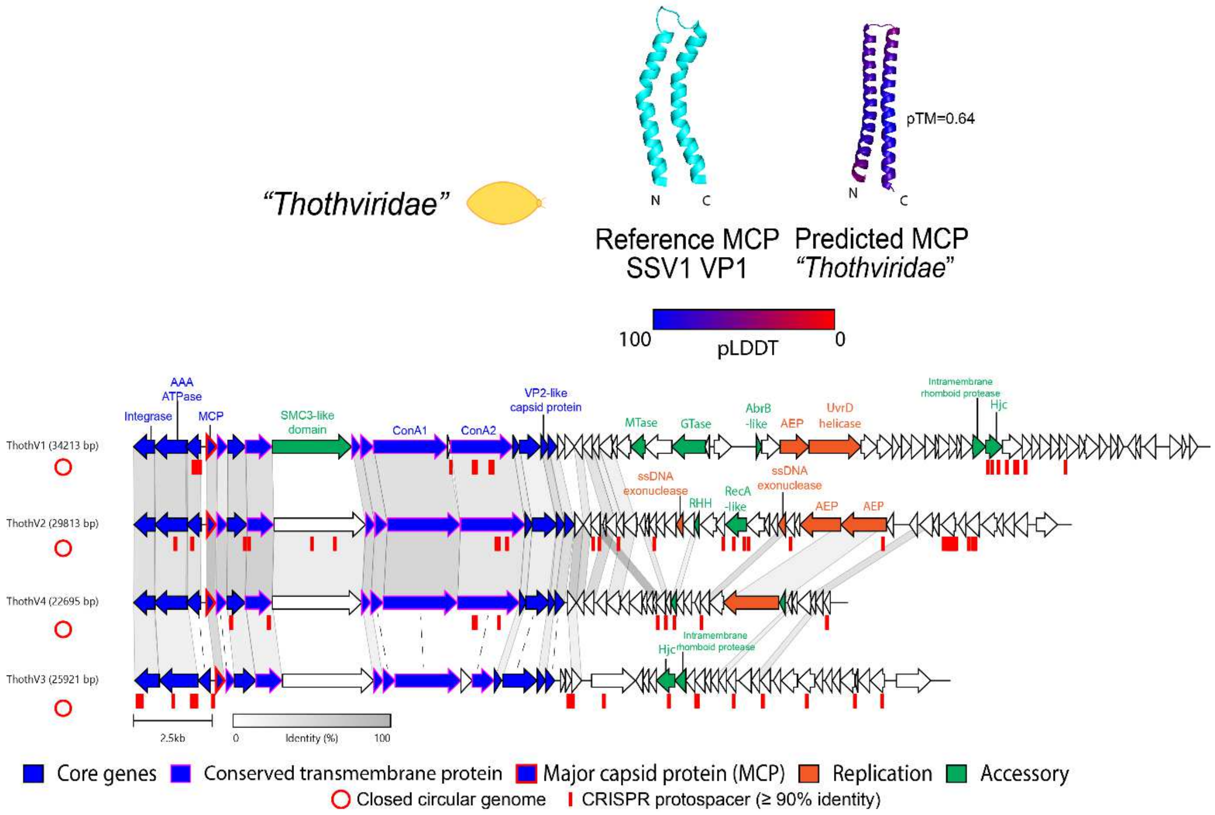

Supplementary Text Figure S1C. Genome synteny maps of representative thothviruses. Complete circular genomes are indicated by red circles. Predicted genes (coding sequences) are represented by arrows. Core genes (blue) are encoded by all complete or near-complete family members. Core genes predicted to encode major capsid proteins (MCPs) and transmembrane proteins are additionally distinguished by red and magenta borders, respectively. Replication-associated genes (orange) and accessory genes with high-confidence functional predictions (green) are labelled, where applicable. Non-core genes of unknown function are coloured white. Homologous genes are connected by bars shaded in grayscale according to percent amino acid identity. Functionally conserved genes with sequence similarity below the similarity group threshold (Methods), or that are unconnected due to vertical ordering of the genomes, are instead connected by dashed lines. Protospacers matching published or predicted Thermococcales CRISPR spacers are indicated by vertical red rectangles. Top: AlphaFold3 structure of a representative thothvirus MCP (with pTM score and coloured by PLDDT, blue to red) compared to the MCP VP1 of Sulfolobus spindle-shaped virus 1 (SSV1). N-termini and C-termini are denoted by N and C, respectively.

Thothviruses comprise four complete genomes (22.7-34.2 kb) and share many core features with "*Sethviridae*", including a tyrosine recombinase integrase, AAA ATPase, and transmembrane protein cassette with MCP, disordered SMC3-like protein, and two Concanavalin A-like lectin/glucanase proteins. Shared soft-core proteins include Hjcs and rhomboid proteases. However, "*Thothviridae"* display

substantial architectural divergence, justifying their designation as a distinct family (alongside VipTree analyses confirming sequence divergence – Supplementary Fig. 6). Key differences include inverted orientation of integrase and ATPase genes relative to the core module. Further, whereas the SMC3-like protein and two ConA-like ORFs are each interspaced with one small transmembrane protein in sethviruses, the two ConA-like ORFs lack intervening genes in two out of four thothvirus genomes, and two transmembrane proteins lie between these and the SMC3-like homolog. Viruses of this family also encode a core gene with significant similarity to the VP2, minor capsid protein encoded by SSV1 [6]. DNA replication modules vary and include an AEP, two AEPs (single or paired), UvrD family helicases, or ORFs of unknown function in the corresponding locus. The hypervariable regions of thothviruses remain poorly annotated, despite our extensive search efforts.

SECTION 1D: *ANUBISVIRIDAE*

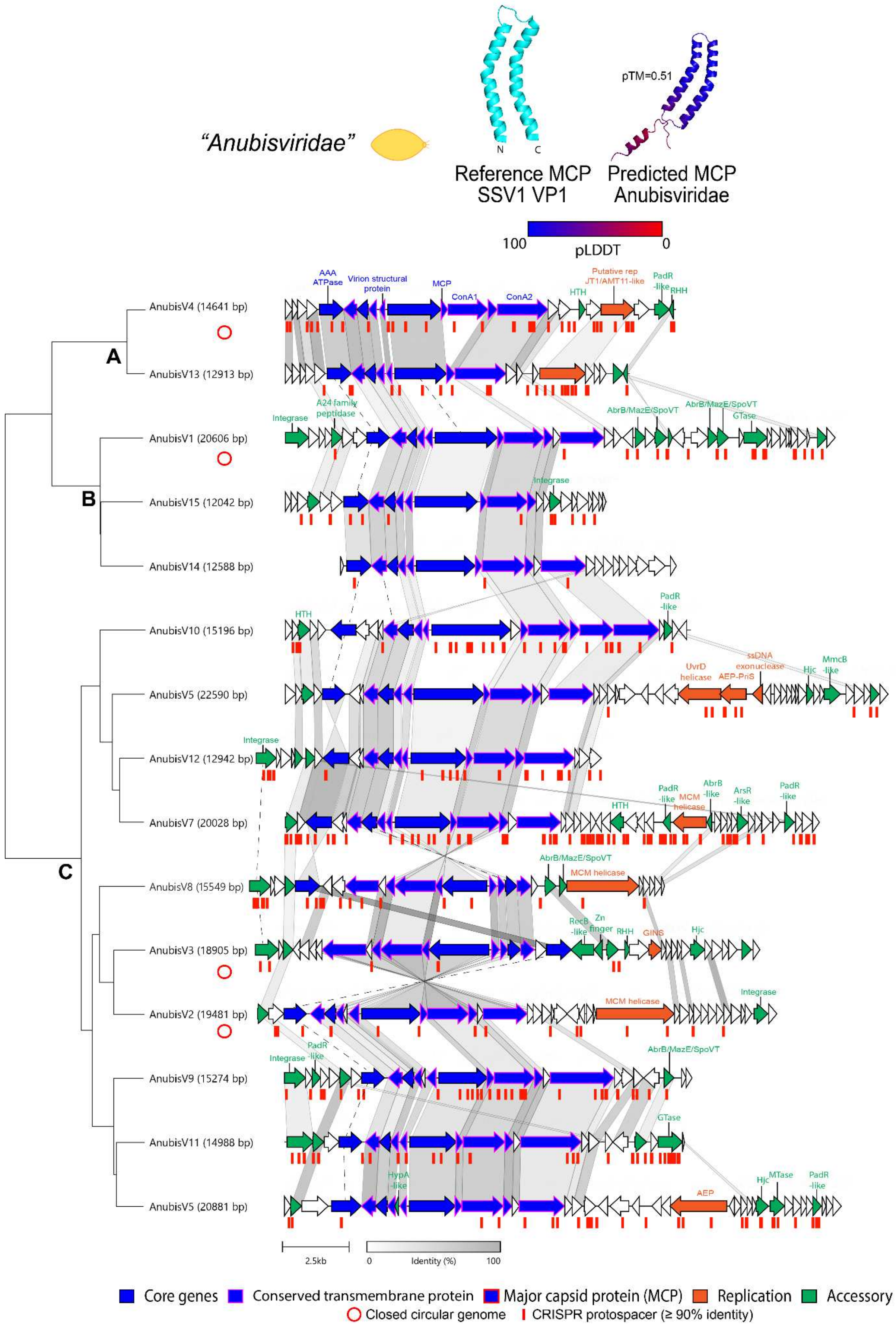

Supplementary Text Figure S1D. Genome synteny maps of representative anubisviruses. ViPTree proteomic phylogeny (left) supports three distinct clades (A-C). Complete circular genomes are indicated by red circles. Predicted genes (coding sequences) are represented by arrows. Core genes (blue) are encoded by all complete or near-complete family members. Core genes predicted to encode major capsid proteins (MCPs) and transmembrane proteins are additionally distinguished by red and magenta borders, respectively. Replication-associated genes (orange) and accessory genes with high-confidence functional predictions (green) are labelled, where applicable. Non-core genes of unknown function are coloured white. Homologous genes are connected by bars shaded in grayscale according to percent amino acid identity. Functionally conserved genes with sequence similarity below the similarity group threshold (Methods), or that are unconnected due to vertical ordering of the genomes, are instead connected by dashed lines. Protospacers matching published or predicted Thermococcales CRISPR spacers are indicated by vertical red rectangles. Top: AlphaFold3 structure of a representative anubisvirus MCP (with pTM score and coloured by PLDDT, blue to red) compared to the MCP VP1 of Sulfolobus spindle-shaped virus 1 (SSV1). N-termini and C-termini are denoted by N and C, respectively.

Anubisviruses include four complete and ten near-complete circular genomes (14.6-20.6 kb), exhibiting a core-variable genome structure similar to "*Sethviridae"* and "*Thothviridae*". Core regions include an AAA ATPase and a cassette containing the predicted MCP and a pair of ConA-like homologs, among several smaller ORFs with predicted transmembrane domains. Features unique to this family include a large core ORF lacking detectable similarity to known proteins and the MCP is located between ConA-like genes rather than adjacent to the ATPase. Transmembrane ORFs are encoded in an orientation opposite to that observed in the "*Sethviridae"* and "*Thothviridae*". One complete genome lacks a predicted integrase, suggesting episomal replication.

As with other spindle-shaped viruses, replication modules are diverse in anubisviruses. Some genomes encode rolling circle replication initiators found in cryptic plasmids (pRT1-like Rep proteins) [3], while others encode theta replication initiating MCM helicases, AEPs, or nuclease-primase-helicase fusion proteins. Several viral genomes lack identifiable replication-associated genes.

SECTION 2: Head-tail viruses of Thermococcales

Head-tail viruses infecting Thermococcales share hallmark components of virion morphogenesis of *Caudoviricetes* infecting both bacteria and archaea. These include an HK97-fold MCP, a large terminase subunit (TerL), portal protein, capsid maturation protease, and various tail-related proteins [7].

Based on genome-wide proteomic comparisons, we propose four distinct families of head-tail viruses, each forming a monophyletic clade under established demarcation criteria [8,9]. Three of these families are phylogenetically distinct from all previously recognized *Caudoviricetes*, representing novel viral taxa. We name them "*Satanviridae*," "*Astarothviridae*," and "*Beelzebubviridae*" after Satan, Astaroth, and Beelzebub (the "unholy trinity" that oversees the Judeo-Christian hell in John Milton's Paradise Lost). The fourth family group with the known *Ekchuahviridae*, previously inferred to infect methanotrophic Methanophagales (ANME-1) archaea from deep-sea sediments [10].

SECTION 2A: “*SATANVIRIDAE”*

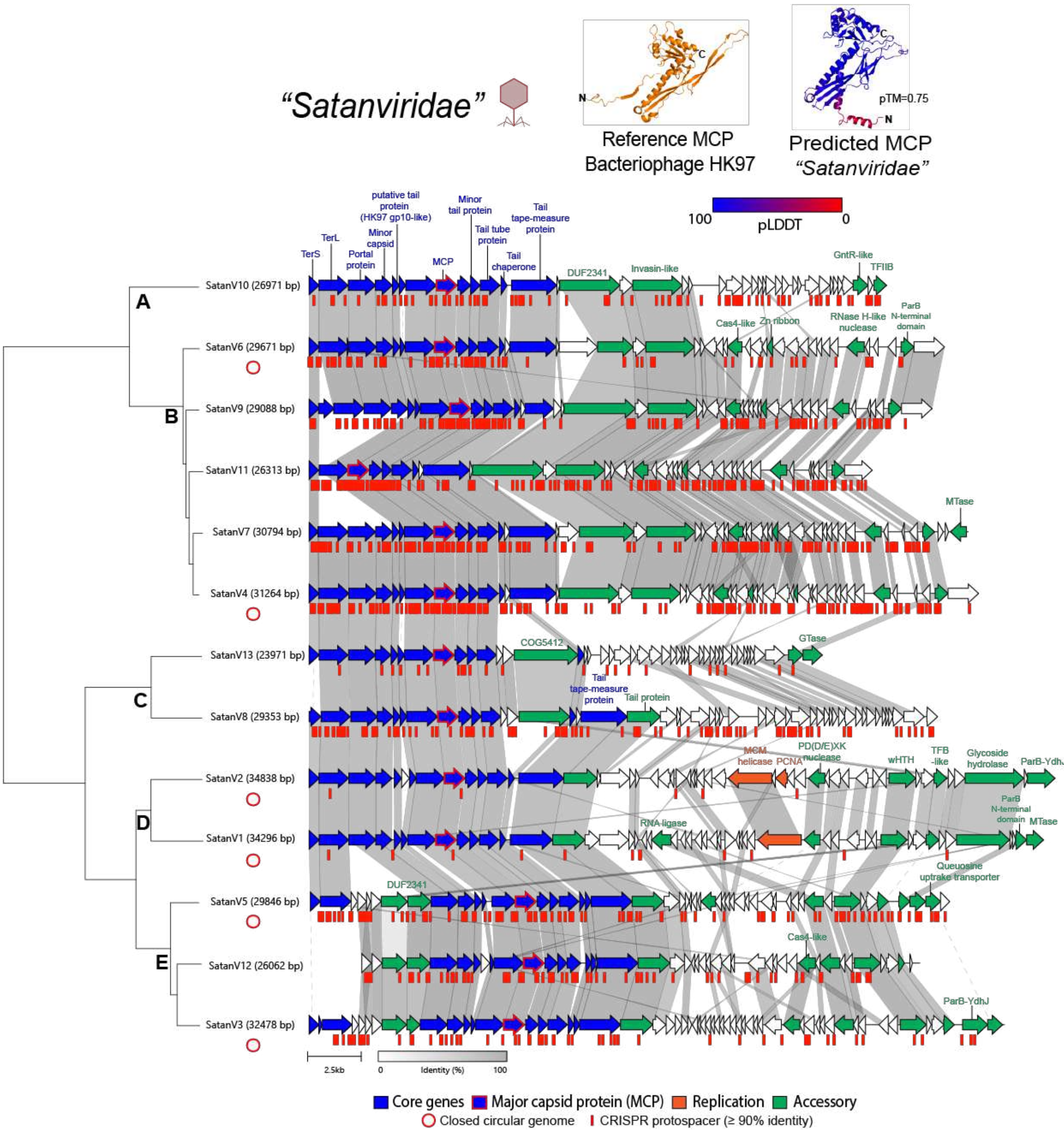

Supplementary Text Figure S2A. Genome synteny maps of representative satanviruses. ViPTree proteomic phylogeny (left) supports five distinct clades (A-E). Complete circular genomes are indicated by red circles. Predicted genes (coding sequences) are represented by arrows. Core genes (blue) are encoded by all complete or near-complete family members. Core genes predicted to encode major capsid proteins (MCPs) are additionally distinguished by red borders. Replication-associated genes (orange) and accessory genes with high-confidence functional predictions (green) are labelled, where applicable. Non-core genes of unknown function are coloured white. Homologous genes are connected by bars shaded in grayscale according to percent amino acid identity. Functionally conserved genes with sequence similarity below the similarity group threshold (Methods), or that are unconnected due to vertical ordering of the genomes, are instead connected by

dashed lines. Protospacers matching published or predicted Thermococcales CRISPR spacers are indicated by vertical red rectangles. Top: AlphaFold3 structure of a representative satanvirus MCP (with pTM score and coloured by PLDDT, blue to red) compared to the MCP of Bacteriophage HK97. N-termini and C-termini are denoted by N and C, respectively.

Satanviruses are the most frequently targeted mobile genetic elements (MGEs) by Thermococcales CRISPR-Cas systems, with 2,348 protospacer matches across 62 contigs (averaging 37.9 matches per contig). We identified thirteen representative sequences (those with complete or near-complete genomes) across five distinct clades (SatanA-E), six of which display direct terminal repeats, suggesting typical genome lengths of 29-36 kbp. These viruses are characterized by a conserved syntenic virion morphogenesis module encoding an HK97-fold MCP, a phage Mu gpF-like protein, small and large terminase subunits (TerS and TerL), portal protein, tail tube and tape measure proteins, and probable tail assembly chaperones. Notably, no integrases are predicted, indicating a likely obligate lytic lifecycle akin to other head-tail bacterial and archaeal viruses.

Members of SatanA and SatanB encode a DUF2341-like ConA (MJ1470 superfamily) and an invasin-like protein with immunoglobulin-like β-sandwich folds, potentially involved in glycan binding and host recognition. In other clades, this locus instead encodes smaller phage tail-like proteins, such as a tail-like protein homologous to a putative *Archaeoglobus* provirus gene (WP_290726695.1). Notably, members of SatanE also encode DUF2341 homologs as a pair of shorter ORFs in an unusual location, between the TerL and portal proteins.

Members of SatanB, SatanD, and SatanE encode Cas4-like nucleases and ParB/Sulfiredoxin domain-containing proteins, though these are not invariably present. All identified ParB-like proteins contain an N-terminal ParB domain, sometimes fused to a YhdJ-like DNA methylase domain (ParB-YhdJ). The absence of ParA homologs suggests that these are not involved in classical ParABS genome partitioning [11]. However, similar ParB fusion proteins may function in anti-viral defense [12,13].

Only SatanD members encode identifiable DNA replication proteins. SatanV1 encodes an MCM helicase and SatanV2 encodes an MCM helicase as well as a DNA polymerase sliding clamp (PCNA). Most other ORFs remain unannotated, though some encode predicted transcriptional regulators (Zn finger, TFB-like).

SECTION 2B: "*ASTAROTHVIRIDAE*"

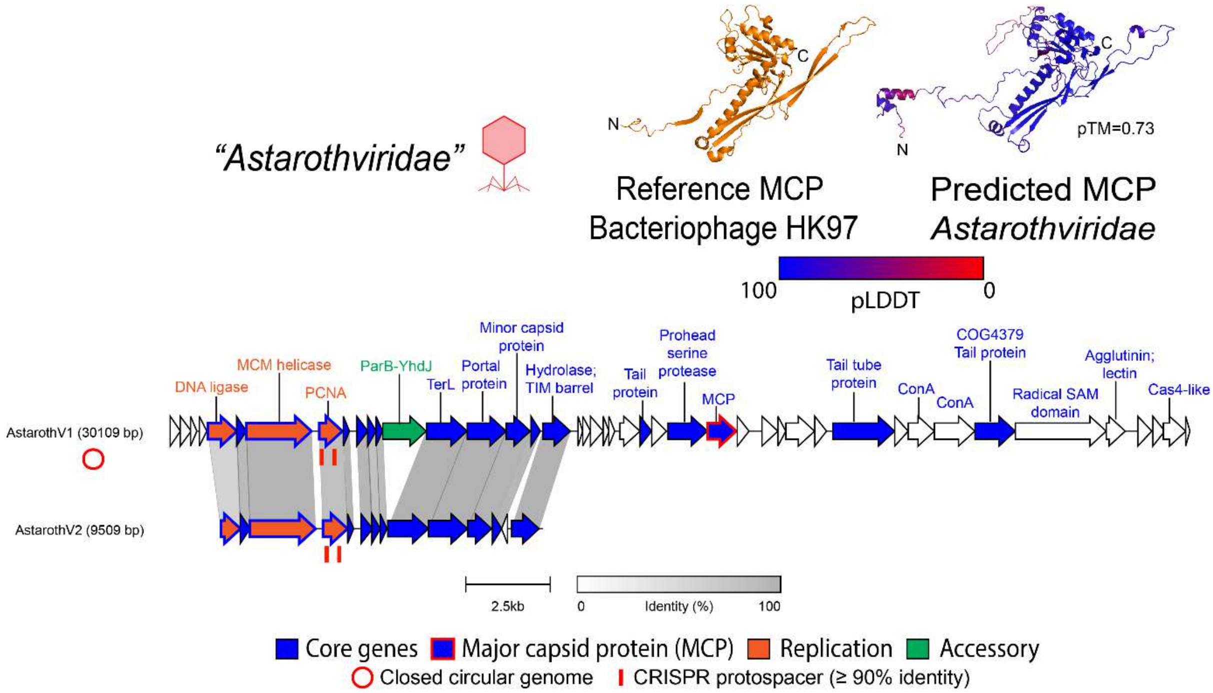

Supplementary Text Figure S2B. Genome synteny maps of representative astarothviruses. Complete circular genomes are indicated by red circles. Predicted genes (coding sequences) are represented by arrows. Core genes (blue) are encoded by both genomes and/or are expected to be encoded by yet unknown family members. Core genes predicted to encode major capsid proteins (MCPs) are additionally distinguished by red borders. Replication-associated genes (orange) are considered tentatively core to astarothviruses and have been additionally assigned blue borders. Accessory genes with high-confidence functional predictions (green) are labelled, where applicable. Non-core genes of unknown function are coloured white. Homologous genes are connected by bars shaded in grayscale according to percent amino acid identity. Functionally conserved genes with sequence similarity below the similarity group threshold (Methods), or that are unconnected due to vertical ordering of the genomes, are instead connected by dashed lines. Protospacers matching published or predicted Thermococcales CRISPR spacers are indicated by vertical red rectangles. Top: AlphaFold3 structure of a representative astarothvirus MCP (with pTM score and coloured by PLDDT, blue to red) compared to the MCP of Bacteriophage HK97. N-termini and C-termini are denoted by N and C, respectively.

Astarothviruses are represented by a single complete circular genome (30.1 kb) and one partial genome (9.5 kb). Both encode a partial virion morphogenesis module including TerL, portal protein, and minor capsid protein, whereas the HK97-fold MCP was identified only in the complete genome of AstarothV1. Nearby genes include a predicted TIM barrel amidohydrolase, and a replication module consisting of a DNA ligase, MCM helicase and PCNA, mirroring the architecture of SatanD members. AstarothV1 also encodes a ParB-YhdJ gene (observed also in "*Satanviridae"* members) adjacent to TerL, which is absent from the partial genome, supporting its likely accessory gene status. In contrast to "*Satanviridae*", astarothvirus MCP and tail

genes are dispersed throughout the genome rather than clustered into a single operon but are likely to be components from the family defining core when more representatives are identified and characterized.

SECTION 2C: “*BEELZEBUBVIRIDAE”*

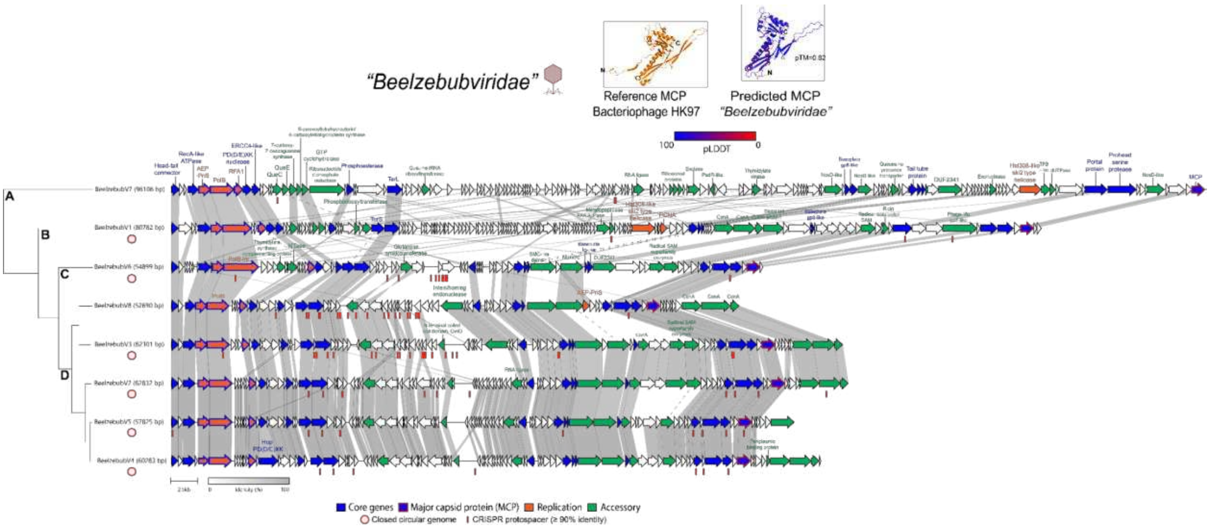

Supplementary Text Figure S2C. Genome synteny maps of representative beelzebubviruses. ViPTree proteomic phylogeny (left) supports four distinct clades (A-D). Complete circular genomes are indicated by red circles. Predicted genes (coding sequences) are represented by arrows. Core genes (blue) are encoded by all complete or near-complete family members. Core genes predicted to encode major capsid proteins (MCPs) are additionally distinguished by red borders. Replication-associated genes (orange) and accessory genes with high-confidence functional predictions (green) are labelled, where applicable. Non-core genes of unknown function are coloured white. Homologous genes are connected by bars shaded in grayscale according to percent amino acid identity. Functionally conserved genes with sequence similarity below the similarity group threshold (Methods), or that are unconnected due to vertical ordering of the genomes, are instead connected by dashed lines. Protospacers matching published or predicted Thermococcales CRISPR spacers are indicated by vertical red rectangles. Top: AlphaFold3 structure of a representative beelzebubvirus MCP (with pTM score and coloured by PLDDT, blue to red) compared to the MCP of Bacteriophage HK97. N-termini and C-termini are denoted by N and C, respectively.

Beelzebubviruses are the largest putative head-tail viruses recovered, with complete circular genomes ranging from 54.9-80.7 kb. They exhibit high diversity in genome architecture and are assigned to four probable genus-level clades (BeelzebubA-D), of which three have complete representatives. In addition to the virion morphogenesis components (including the MCP, portal protein, terminase subunits and tail proteins), the core of this family includes a RecA-like ATPase, a PD(D/E)XK nuclease (intein-containing in BeelzebubV4 (60,283 bp)), an Agglutinin C-like lectin, and homologs of DUF2341 of the ConA-like lectin/glucanase superfamily, typically proximal to predicted tail genes.

Synteny is variable due to frequent insertion of auxiliary modules. For example, a helix-hairpin-helix (HhH) protein is typically adjacent to the TerS, but in BeelzebubV1 and BeelzebubV7, this region is expanded by auxiliary metabolic genes (AMGs), including those for 7-deazaguanine modification (e.g., QueA, QueE) [14], which can protect viral DNA from cleavage by host restriction endonucleases [15]. Other AMGs

with probable roles in nucleotide or amino acid metabolism include glutamine amidotransferase and glutamine synthase encoded by Beelzebub6 and Beelzebub8.

Replication machinery is well defined and universally encoded between a RecA ATPase and PD(D/E)XK nuclease, including: AEP-PriS, family B DNA polymerase (PolB), replication factor A1 (RFA1) [16]. Notably, two forms of PolB are found; a long, intein-containing PolB variant (877-1,076 amino acids) or a shorter PolB (678-733 amino acids) lacking the intein but preserving essential functional motifs (see main text and Fig. 4). The region between RFA1 and TerS appears to display frequent gene turnover. Additional replication genes, distal to the conserved module, were observed in some genomes. Both BeelzebubV7 (96,106 bp) and BeelzebubV1 (80,782 bp) encode homologs of Hel308 replicative helicases encoded by Thermococcales and other archaea [17], and the latter genome additionally encodes a PCNA. BeelzebubV8 (52,690) encodes a second, truncated AEP-PriS.

SECTION 2D: *EKCHUAHVIRIDAE*

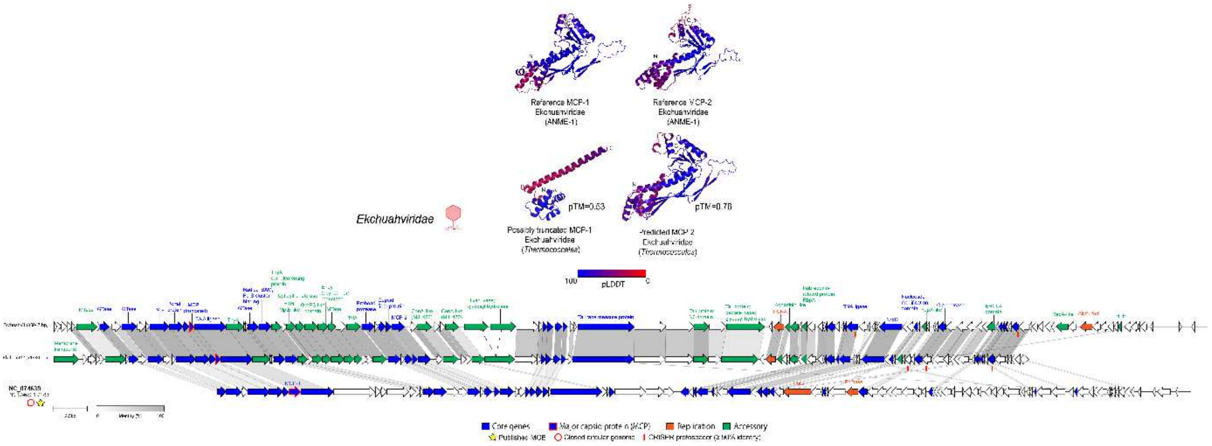

Supplementary Text Figure S2D. Genome synteny maps of representative ekchuahviruses. Published family members are marked by gold stars (here a reference genome, see below for details), and complete circular genomes are indicated by red circles. Predicted genes (coding sequences) are represented by arrows. Core genes (blue) are encoded by all complete or near-complete family members. Core genes predicted to encode major capsid proteins (MCPs) are additionally distinguished by red borders. Replication-associated genes (orange) and accessory genes with high-confidence functional predictions (green) are labelled, where applicable. Non-core genes of unknown function are coloured white. Homologous genes are connected by bars shaded in grayscale according to percent amino acid identity. Functionally conserved genes with sequence similarity below the similarity group threshold (Methods), or that are unconnected due to vertical ordering of the genomes, are instead connected by dashed lines. Protospacers matching published or predicted Thermococcales CRISPR spacers are indicated by vertical red rectangles. Top: AlphaFold3 structure of a representative ekchuahvirus MCP-2 and possible truncated MCP-1 (with pTM score and coloured by PLDDT, blue to red) compared to the MCP-1 and MCP-2 of a published ekchuahvirus (NC_074639). N-termini and C-termini are denoted by N and C, respectively.

In addition to the three, novel head-tail families described above, we identified viruses that group with *Ekchuahviridae* (Supplementary Fig. 4), a family predicted to infect methanotrophic ANME-1 archaea [10]. Two representative genomes were identified that are comparable in length to complete genomes of ekchuahviruses (71,930 bp and 85,047 bp) but do not show evidence of terminal redundancy. In the synteny diagram above, we compare the two candidate Thermococcales *Ekchuahviridae* with that of a published ANME-1 infecting candidate (NC_074639).

Recognized members of this family encode two HK97-like MCPs, one of which (MCP-2) is conserved in those revealed by this study. The canonical MCP-1 in both representative genomes is replaced by a shorter, divergent protein. While sequence similarity is low, AlphaFold3 predicts a structure resembling HK97 MCP α-helical bundles, suggesting functional conservation. Otherwise, predicted and existing family members share many syntenic relationships across the length of their genomes, including two largely contiguous morphogenetic modules and other gene

clusters with consistent orientation. However, synteny is broken by long segments encoding distinct gene sets (e.g. an enzymatic cluster upstream of the MCP-2).

Replication machinery is also distinct between existing ANME-1-targeting viruses and predicted Thermococcales-targeting viruses; whereas the former encode an RNA-primed PolB (rPolB) and AEP, the latter instead encode a PCNA alongside an AEP. Additionally, these predicted ekchuahviruses show considerable variation in the tail morphogenesis module compared to known family members, likely a requirement to extend their host range to Thermococcales. Such differences suggest that the new family members reported here are likely to constitute a new genus within *Ekchuahviridae*.

It should be noted that these viral contigs have few spacer hits from Thermococcales (although those hits are >90% sequence identity) and are targeted by spacers from the ANME-1 dataset with similar identity. It is possible that these viruses have an expanded host range, capable of infecting a variety of marine archaea, and/or a host-switch has occurred during the evolution of *Ekchuahviridae*. Also possible is that *Ekchuahviridae* are ANME-1 infecting viruses that share genes in common with as yet unidentified Thermococcales infecting MGEs, and so share spacer hits. Thus, we cautiously refrain from strictly classifying these as Thermococcales viruses.

SECTION 3: Filamentous viruses of Thermococcales

Our analysis predicts two distinct families of filamentous viruses infecting Thermococcales. Both encode MCPs with the canonical SIRV2-like fold, characteristic of viruses within the realm *Adnaviria* [18]. One family is entirely novel, which we name "*Samediviridae*" after Baron Samedi, a spirit that governs death and the underworld in Haitian Vodou tradition. The other represents an extension of the family, *Ahmunviridae*, originally associated with Methanophagales archaea [10].

SECTION 3A: “*SAMEDIVIRIDAE”*

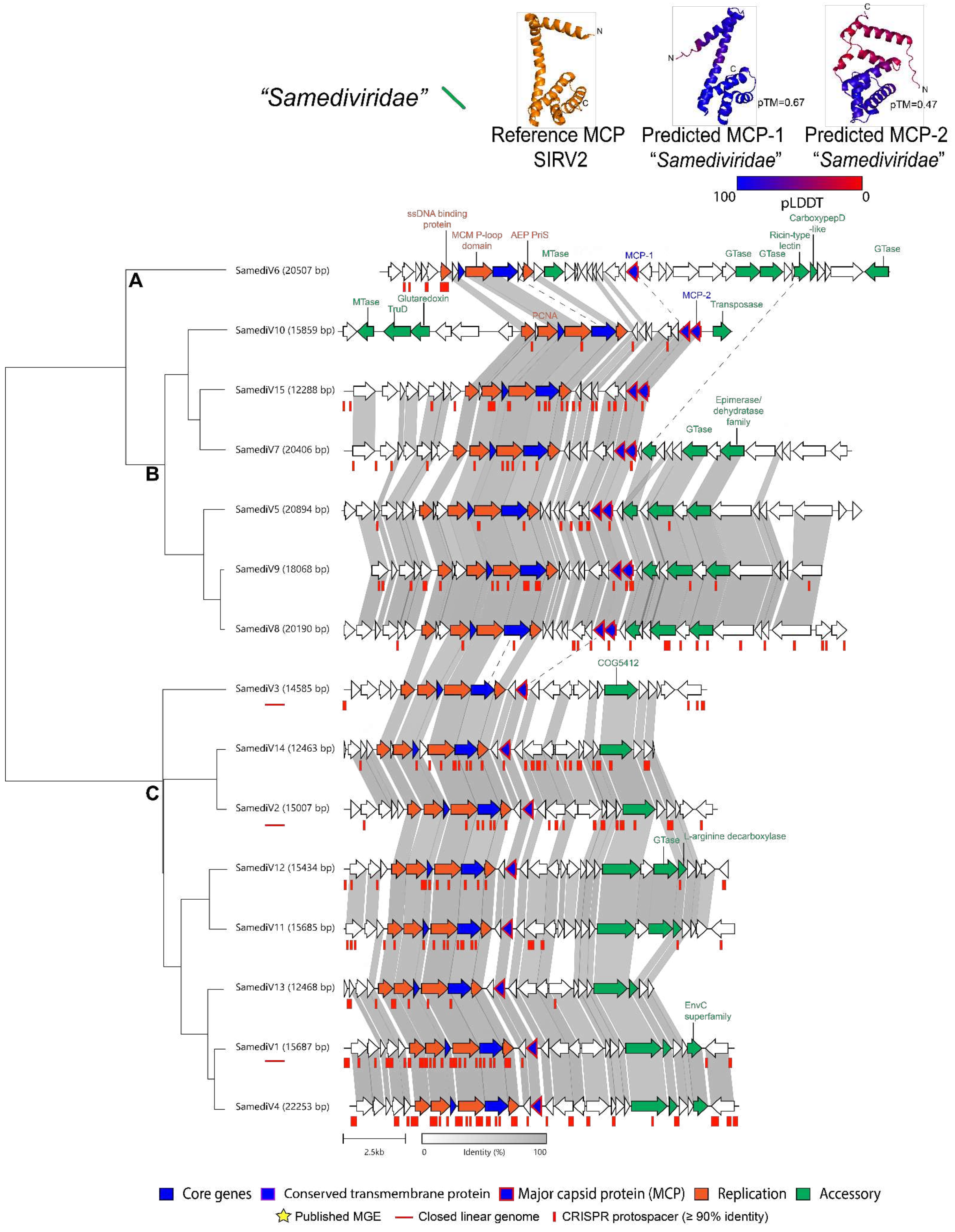

Supplementary Text Figure S3A. Genome synteny maps of representative samediviruses. ViPTree proteomic phylogeny (left) supports three distinct clades (A-C). Complete linear genomes are indicated by horizontal red lines. Predicted genes (coding sequences) are represented by arrows. Core genes (blue) are encoded by

all complete or near-complete family members. Core genes predicted to encode major capsid proteins (MCPs) are additionally distinguished by red borders. Replication-associated genes (orange) and accessory genes with high-confidence functional predictions (green) are labelled, where applicable. Non-core genes of unknown function are coloured white. Homologous genes are connected by bars shaded in grayscale according to percent amino acid identity. Functionally conserved genes with sequence similarity below the similarity group threshold (Methods), or that are unconnected due to vertical ordering of the genomes, are instead connected by dashed lines. Protospacers matching published or predicted Thermococcales CRISPR spacers are indicated by vertical red rectangles. Top: AlphaFold3 structure of a representative samedivirus MCP-1 and MCP-2 (with pTM score and coloured by PLDDT, blue to red) compared to the MCP of Sulfolobus islandicus rudivirus 2 (SIRV2). N-termini and C-termini are denoted by N and C, respectively.

Samediviruses are represented by 15 members, three of which have complete linear genomes with inverted terminal repeats. Phylogenomic and comparative analysis of genomic architectures support our delineation of three distinct clades (SamediA-C). SamediA currently contains a single genome (20.5 kb; 44.7% GC), while SamediB includes multiple genomes (12.3-20.8 kb; 34-54.5% GC) and SamediC (12.4-22.2 kb; 42.9-48.1% GC). All four predicted complete viral genomes belong to the SamediC. Unlike most other *Thermococcales* MGE families, samediviruses have a relatively compact core genome, largely composed of genes of unknown function. However, two universally conserved, functionally annotated genes include MCP (MCP-1) (with a canonical predicted SIRV2-type fold typical of members of the realm *Adnaviria* [19] and what appears to be a an AAA+ ATPase domain fused to a DNA-binding wHTH domain (COG3355) that is frequently adjacent to a PCNA homolog.

Clade-specific genes include those encoding a probable carbohydrate manipulation module, comprising a nucleoside-diphosphate sugar epimerase, two glycosyltransferases (GTases), and an agglutinin-like lectin in SamediA. A similar module is also present in SamediB, though the components are encoded in the opposite orientation and only include one GTase. In SamediC, this region displays a distinct architecture and contains ORFs lacking clear homologs, with one exception; all members of SamediC encode a COG5412 domain-containing probable minor structural protein. All samediviruses encode a core replication machinery comprising a single-stranded DNA binding protein, PCNA (absent only in SamediV6), MCM-like protein, and an AEP PriS.

SECTION 3B: *AHMUNVIRIDAE*

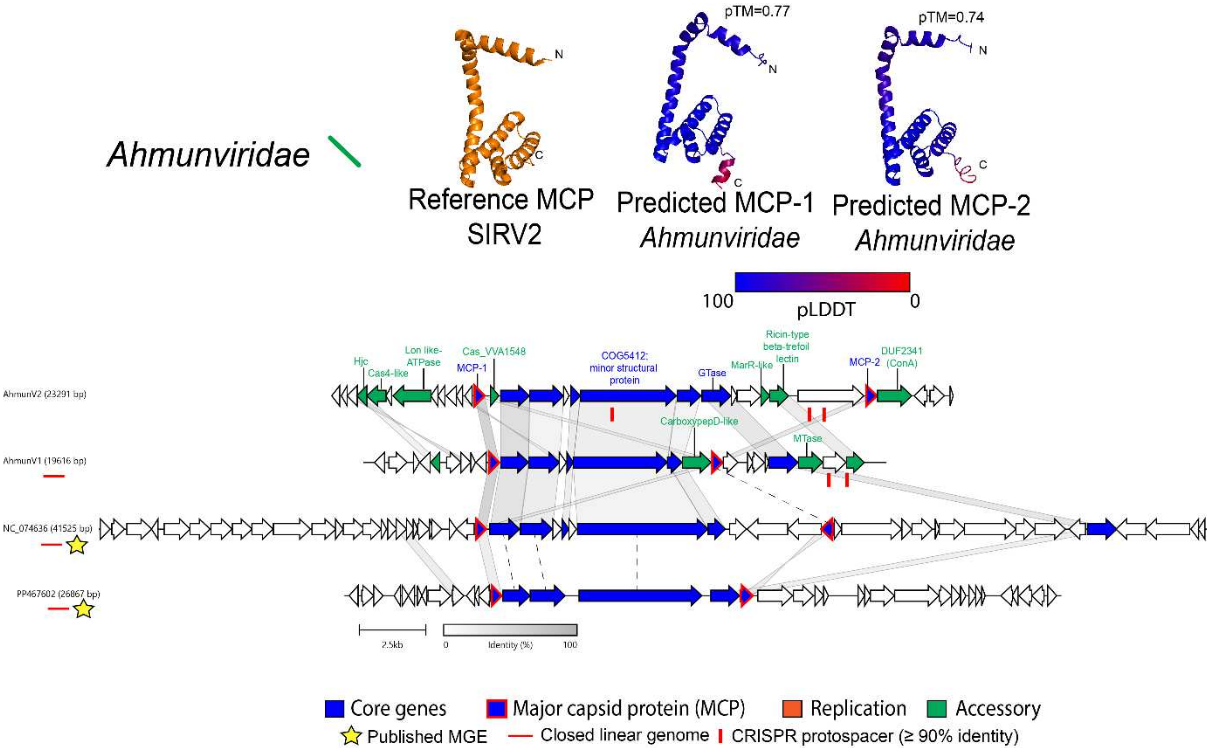

Supplementary Text Figure S3B. Genome synteny maps of representative ahmunviruses. Published family members are marked by gold stars (here, reference genomes from NCBI), and complete linear genomes are indicated by horizontal red lines. Predicted genes (coding sequences) are represented by arrows. Core genes (blue) are encoded by all complete or near-complete family members. Core genes predicted to encode major capsid proteins (MCPs) are additionally distinguished by red borders. Replication-associated genes (orange) and accessory genes with high-confidence functional predictions (green) are labelled, where applicable. Non-core genes of unknown function are coloured white. Homologous genes are connected by bars shaded in grayscale according to percent amino acid identity. Functionally conserved genes with sequence similarity below the similarity group threshold (Methods), or that are unconnected due to vertical ordering of the genomes, are instead connected by dashed lines. Protospacers matching published or predicted Thermococcales CRISPR spacers are indicated by vertical red rectangles. Top: AlphaFold3 structure of a representative ahmunvirus MCP-1 and MCP-2 (with pTM score and coloured by PLDDT, blue to red) compared to the MCP of Sulfolobus islandicus rudivirus 2 (SIRV2). N-termini and C-termini are denoted by N and C, respectively.

Our study expands the *Ahmunviridae* family (previously only known to be associated with ANME-1 archaea [10]), with two new viral genomes. One new genome is predicted to be complete and linear (19,616 bp), while the other lacks evidence of terminal redundancy despite being noticeably longer (23,291 bp). To explain relationships revealed by VipTree analysis (Supplementary Fig. 7), the synteny diagram above compares these new sequences to a published *Ahmunviridae*

genome (NC_074636), and a more distantly related *Chiyouviridae* genome (PP467602), indicated by stars.

Despite high variability, both new genomes, share a largely contiguous set of core genes that includes two putative MCPs (MCP-1 and MCP-2), a common feature in members of *Adnaviria* [18]. The pair of MCPs adopt near-identical folds and are close structural homologs of samedivirus and rudivirus MCPs. Despite this and their similar lengths (122 and 131 aa), the MCPs are divergent at the sequence level (~24% amino acid identity). MCP-1 is part of the most positionally conserved block of the core genes, which also includes one of two encoded GTases, a COG5412 domain-containing probable minor structural protein, and other smaller proteins of unknown function. Other core genes, including the MCP-2, the second GTase, a Ricin-type β-trefoil lectin, and Hjc, are located within a more variable region, where the core genes are interspersed with virus-specific genes. The variable gene set includes genes for an auxiliary carboxypeptidase-like regulatory domain-containing protein, DUF2341 domain-containing protein of the ConA superfamily and two Cas-like proteins, a member of VVA1548 putative Cas family and a Cas4-like exonuclease.

As with *Ekchuahviridae* mentioned above, we are cautious to denote these as exclusively Thermococcales viruses. However, unlike *Ekchuahviridae,* these candidate Thermococcales *Ahmunviridae* genomes are not targeted by ANME-1 spacers. This may suggest switching of archaeal hosts has occurred during the evolution of *Ahmunviridae.*

SECTION 4: Bacilliform viruses of Thermococcales

We identified a novel group of bacilliform viruses distantly related to the family *Clavaviridae*, currently represented by a single isolate, Aeropyrum pernix bacilliform virus 1 (APBV-1) [20]. APBV-1 produces bacilliform virions using an MCP consisting of two α-helices joined by a β-hairpin [21]. The fold of this protein and the overall virion organization of APBV-1 resembles that of spindle-shaped viruses, leading to the hypothesis that archaeal spindle-shaped viruses may have evolved from a clavavirus-like ancestors [22]. We propose a new family, "*Mammanviridae"*, to accommodate these newly discovered Thermococcales-associated viruses, naming it after Mamman Brigitte, a Hatian Vodou spirit associated with the underworld.

SECTION 4A: "*MAMMANVIRIDAE"*

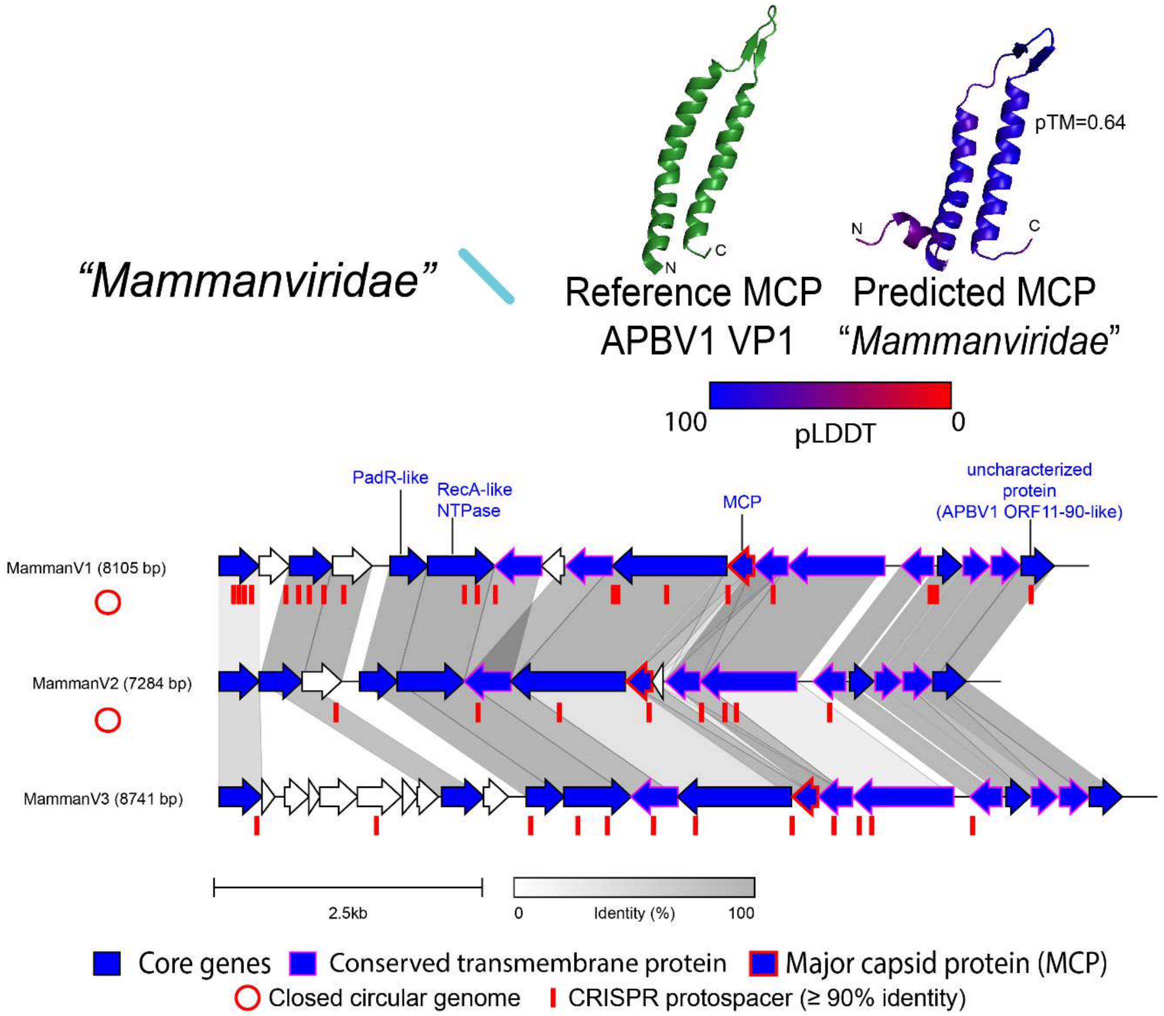

Supplementary Text Figure S4A. Genome synteny maps of representative mammanviruses. Complete circular genomes are indicated by red circles. Predicted genes (coding sequences) are represented by arrows. Core genes (blue) are encoded by all complete or near-complete family members. Core genes predicted to encode major capsid proteins (MCPs) are additionally distinguished by red borders. Replication-associated genes (orange) and accessory genes with high-confidence functional predictions (green) are labelled, where applicable. Non-core genes of unknown function are coloured white. Homologous genes are connected by bars

shaded in grayscale according to percent amino acid identity. Functionally conserved genes with sequence similarity below the similarity group threshold (Methods), or that are unconnected due to vertical ordering of the genomes, are instead connected by dashed lines. Protospacers matching published or predicted Thermococcales CRISPR spacers are indicated by vertical red rectangles. Top: AlphaFold3 structure of a representative mammanvirus MCP (with pTM score and coloured by PLDDT, blue to red) compared to the MCP VP1 of Aeropyrum pernix bacilliform virus 1 (APBV1). N-termini and C-termini are denoted by N and C, respectively.

Mammanviruses are represented by three genomes, two of which are predicted to be circular and hence complete. With lengths of 7.2 and 8.1 kbp, members of this family have the shortest genomes uncovered in our study, yet the genomes are slightly larger than that of APBV-1 (5.3 kb). Mammanviruses have a highly uniform genome organization, with most (>70%) of the genes being syntenic and shared by each member virus. All encode a predicted APBV1 VP1-like MCP and a homolog of the APBV1 uncharacterized protein ORF 11-90. Other universally conserved proteins include a RecA-like NTPase, a PadR-like probable transcriptional regulator, and several transmembrane proteins of unknown function. We were unable to predict replication associated genes in these genomes.

SECTION 5: Ovoid viruses of Thermococcales

We identified a group of putative ovoid viruses that likely represent a novel lineage. We propose the family name "*Angraviridae*", derived from Angra Mainyu, the Zoroastrian god of death and destruction.

SECTION 5A: *"ANGRAVIRIDAE"*

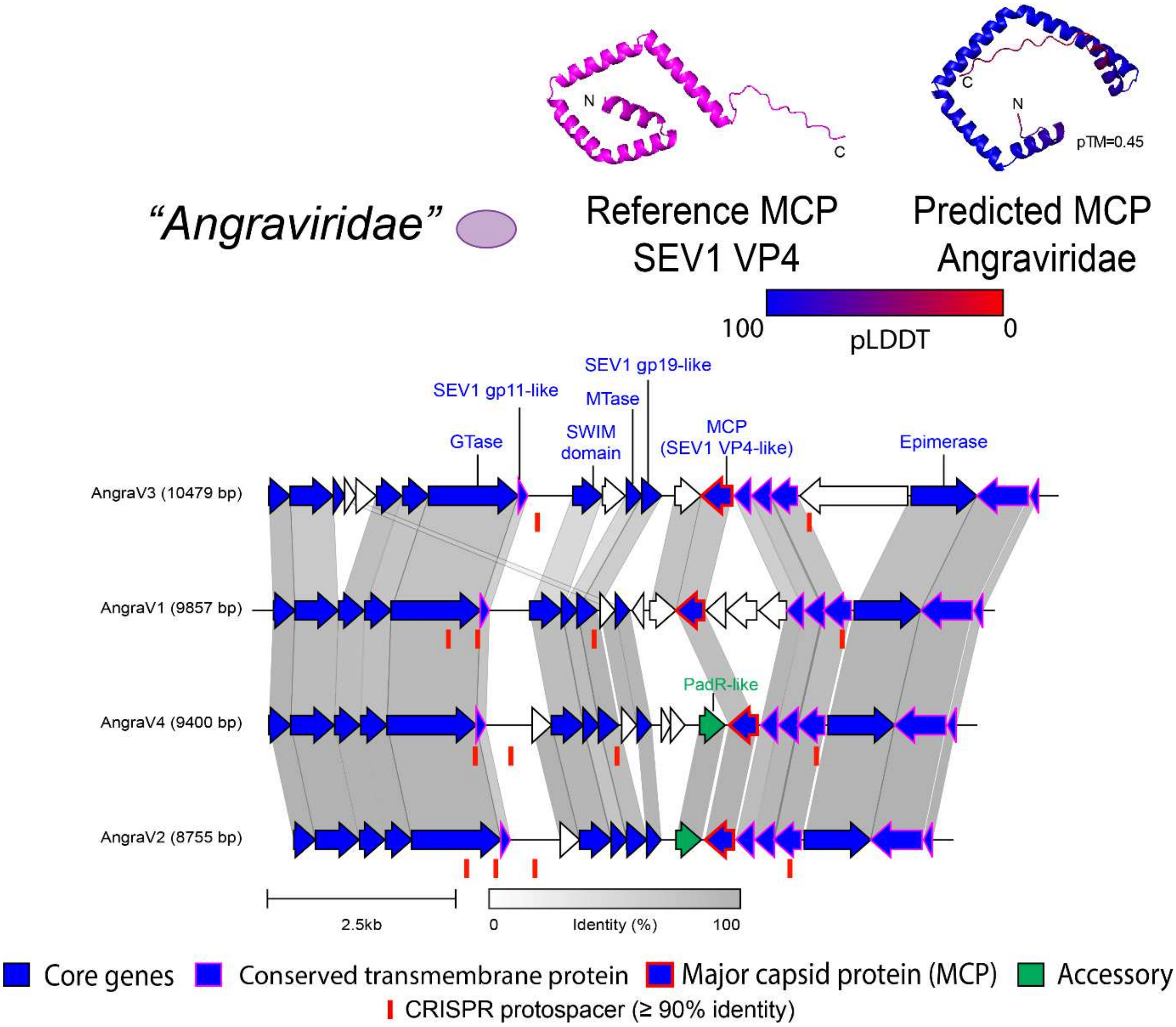

Supplementary Text Figure S5A. Genome synteny maps of representative angraviruses. Predicted genes (coding sequences) are represented by arrows. Core genes (blue) are encoded by all complete or near-complete family members. Core genes predicted to encode major capsid proteins (MCPs) are additionally distinguished by red borders. Replication-associated genes (orange) and accessory genes with high-confidence functional predictions (green) are labelled, where applicable. Non-core genes of unknown function are coloured white. Homologous genes are connected by bars shaded in grayscale according to percent amino acid identity. Functionally conserved genes with sequence similarity below the similarity group threshold (Methods), or that are unconnected due to vertical ordering of the genomes, are instead connected by dashed lines. Protospacers matching published or predicted Thermococcales CRISPR spacers are indicated by vertical red rectangles. Top: AlphaFold3 structure of a representative angravirus MCP (with pTM

score and coloured by PLDDT, blue to red) compared to the MCP VP4 of Sulfolobus ellipsoid virus 1 (SEV1). N-termini and C-termini are denoted by N and C, respectively.

Angraviruses are represented by four near-complete genomes, ranging from 8.7-10.5 kbp. These genomes are highly conserved, with fewer than one-third of ORFs showing variation across representatives. All members encode a putative major structural protein with an α-helical fold that closely resembles the VP4 MCP of Sulfolobus ellipsoid virus 1 (SEV1), the only known member of the family *Ovaliviridae* [23], as well as the unclassified Sulfolobales Beppu virus 1 [24]. Additionally, Angravirus genomes encode two homologs of uncharacterized SEV1 proteins: ORF62/gp11 and ORF52/gp19. Other functionally predicted core genes include a glycotransferase (GTase), a putative epimerase, a methyltransferase, and a SWIM-type domain protein. Beyond these, several additional ORFs are present but most lack confident functional annotation. One notable feature is the presence of PadR-like transcriptional regulators, a common protein in other Thermococcales-associated MGEs.

SECTION 6: Icosahedral viruses of Thermococcales

Some of the most remarkable genomic and structural diversity observed in the Thermococcales virome is observed among predicted icosahedral, tail-less viruses. These viruses possess conserved single jelly-roll (SJR) or double jelly-roll (DJR) major capsid proteins (MCPs), consistent with established icosahedral virus lineages. We propose six distinct families to classify these viruses, each represented by at least one complete genome. Additionally, we identify a seventh, provisional family composed of fragmented genomes. The proposed viral family names are: "*Yamaviridae*", "*Shyamaviridae*", "*Kalaviridae*", "*Shabalaviridae*", "*Karaliviridae*", and "*Chitraguptaviridae*". These are derived from Yama, the king of the Hindu underworld, and several of his assistants, underlings, or aspects: Shyama and Shabala (two watchdog guardians of the underworld), Kala (an epithet of Yama), Karali (an alias of the goddess Kali), and Chitragupta (recordkeeper of the underworld). The seventh putative family, which we provisionally named "*Dandaviridae*" after the Yama Danda, the staff used to inflict punishment on the sinful, is composed of only fragmented genomes.

SECTION 6A: "*YAMAVIRIDAE*"

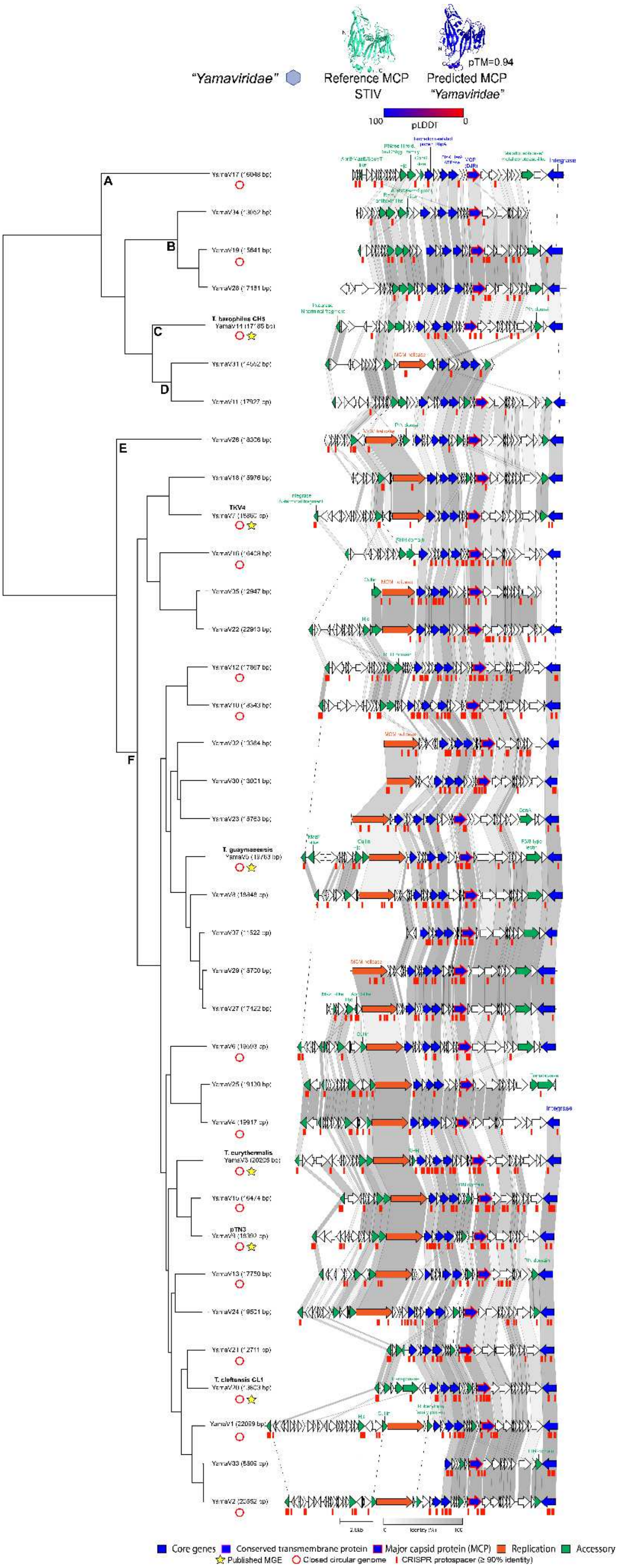

Supplementary Text Figure S6A. Genome synteny maps of representative yamaviruses. ViPTree proteomic phylogeny (left) supports at least six distinct clades (A-F). Published family members are marked by gold stars, and complete circular genomes are indicated by red circles. Predicted genes (coding sequences) are represented by arrows. Core genes (blue) are encoded by all complete or near-complete family members. Core genes predicted to encode major capsid proteins (MCPs) are additionally distinguished by red borders. Replication-associated genes (orange) and accessory genes with high-confidence functional predictions (green) are labelled, where applicable. Non-core genes of unknown function are coloured white. Homologous genes are connected by bars shaded in grayscale according to percent amino acid identity. Functionally conserved genes with sequence similarity below the similarity group threshold (Methods), or that are unconnected due to vertical ordering of the genomes, are instead connected by dashed lines. Protospacers matching published or predicted Thermococcales CRISPR spacers are indicated by vertical red rectangles. Top: AlphaFold3 structure of a representative yamavirus MCP (with pTM score and coloured by PLDDT, blue to red) compared to the MCP of Sulfolobus turreted icosahedral virus (STIV). N-termini and C-termini are denoted by N and C, respectively.

Yamaviruses are the most widely represented icosahedral viruses in our dataset. The founding members include the plasmid pTN3, considered an integrating and episomal provirus, and five related MGEs integrated into *Thermococcales* genomes [25,26]. Our analysis expands this family to 33 complete or near-complete genomes, 27 of which are newly identified. Proteomic analysis suggests multiple layers of intra-clade and inter-clade diversity, with many potential genus-level groups. However, all members maintain a conserved genome framework, with five universally conserved core genes: the predicted DJR MCP, an FtsK-HerA superfamily genome packaging ATPase, an integrase of the tyrosine recombinase superfamily, a rubredoxin-like protein RbpA, and a protein of unknown function. The five genes maintain the overall gene order, though insertions occur between core loci e.g. transcriptional regulators, VapC-like PIN domain proteins. Two major genome regions exhibit high plasticity. The virus-specific accessory gene set encodes metallopeptidase-like proteins, SAM-pointed domain-containing proteins, VapC/PIN domain homologs, transcriptional regulators (AbrB/MazE/SpoVT-like), Hjc homologs, and varying number of uncharacterized proteins. Like other Thermococcales viruses, the replicases also belong to the variable gene set. ~60% of genomes (20/33) encode MCM family helicases, while others encode RuvC/YqgF family nucleases or ribbon-helix-helix (RHH) domain-containing proteins. This variability suggests functional replacement or evolutionary streamlining.

SECTION 6B: “*SHYAMAVIRIDAE*”

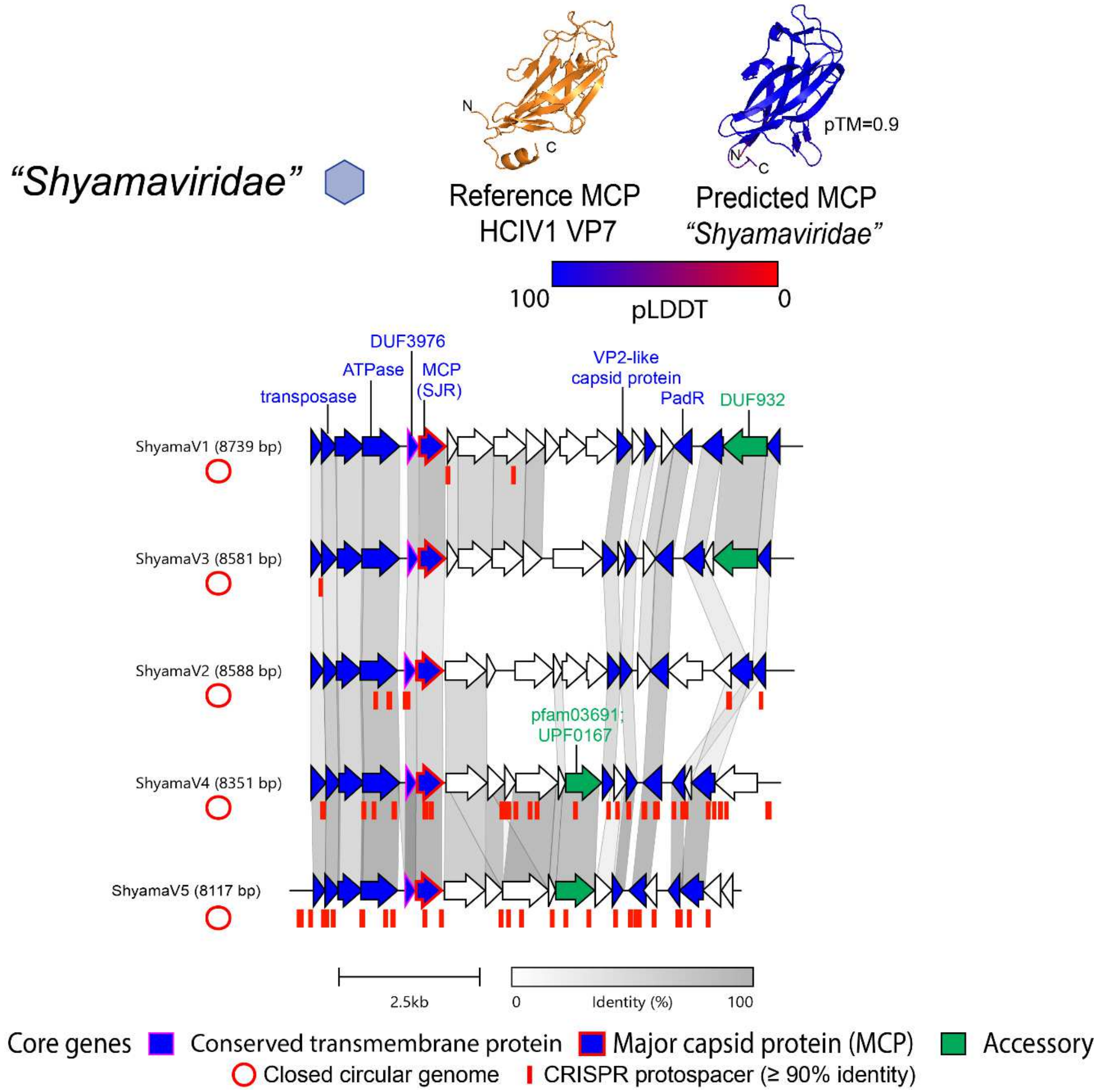

Supplementary Text Figure S6B. Genome synteny maps of representative shyamaviruses. Complete circular genomes are indicated by red circles. Predicted genes (coding sequences) are represented by arrows. Core genes (blue) are encoded by all complete or near-complete family members. Core genes predicted to encode major capsid proteins (MCPs) are additionally distinguished by red borders. Replication-associated genes (orange) and accessory genes with high-confidence functional predictions (green) are labelled, where applicable. Non-core genes of unknown function are coloured white. Homologous genes are connected by bars shaded in grayscale according to percent amino acid identity. Functionally conserved genes with sequence similarity below the similarity group threshold (Methods), or that are unconnected due to vertical ordering of the genomes, are instead connected by dashed lines. Protospacers matching published or predicted Thermococcales CRISPR spacers are indicated by vertical red rectangles. Top: AlphaFold3 structure of a representative shyamavirus MCP (with pTM score and coloured by PLDDT, blue to red) compared to the MCP of Haloarcula californiae icosahedral virus 1 (HCIV1). N-termini and C-termini are denoted by N and C, respectively.

Shyamaviruses are characterized by small circular genomes (8.3-8.7 kb) and currently include five representatives. All members of the family share a set of core genes, including the predicted MCP with a SJR topology, which is most similar to one of the two MCPs, VP7, of Haloarcula californiae icosahedral virus 1 (HCIV1; family *Sphaerolipoviridae*) [27,28], the FtsK-like genome packaging ATPase and a protein homologous to the putative minor structural DNA-binding protein VP2 of fuselloviruses [6]. A provirus with a similar conserved core gene set has been reported in *Archaeoglobus veneficus* [29], a hyperthermophilic archaeon that, like Thermococcales, inhabits deep-sea hydrothermal vents. A conserved transposase-like protein is present in all genomes suggesting a possible integrative lifestyle for shyamaviruses.

SECTION 6C: *"KALAVIRIDAE"*

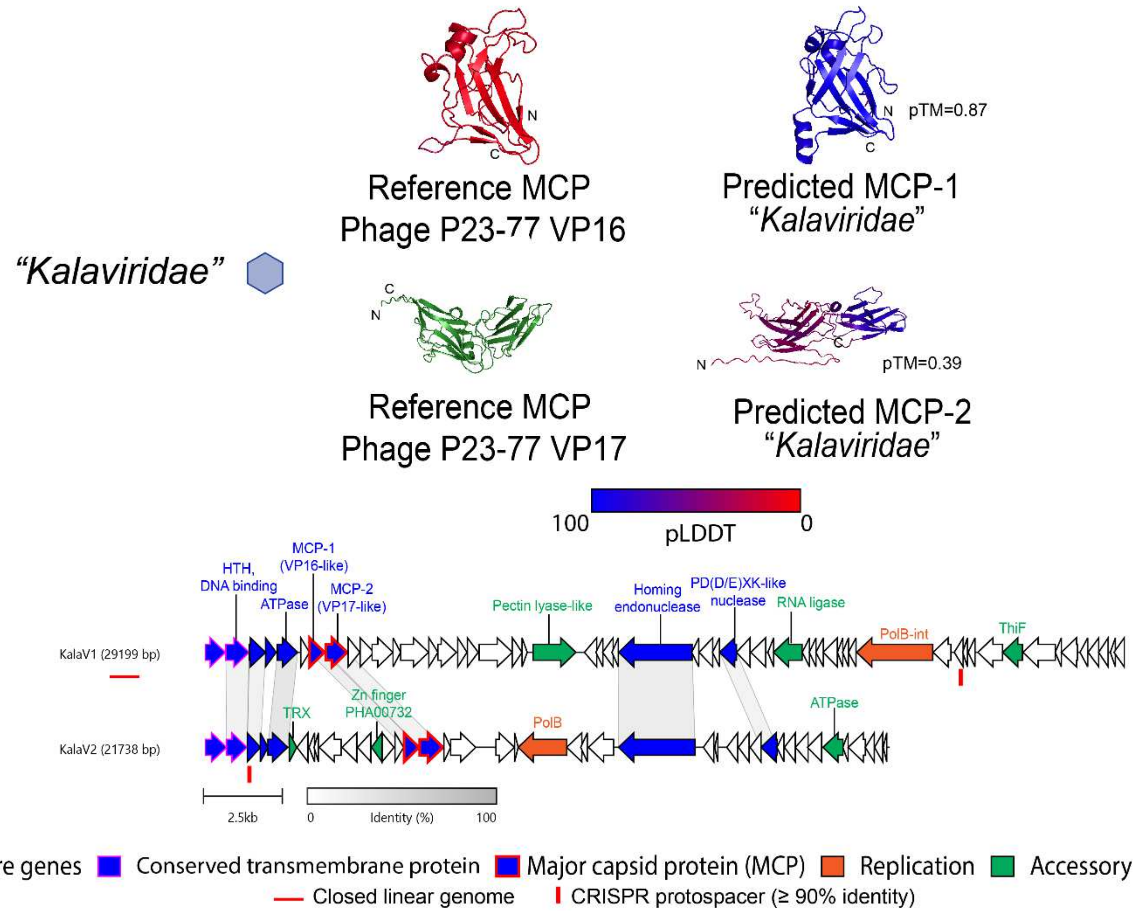

Supplementary Text Figure S6C. Genome synteny maps of representative kalaviruses. Complete linear genomes are indicated by horizontal red lines. Predicted genes (coding sequences) are represented by arrows. Core genes (blue) are encoded by all complete or near-complete family members. Core genes predicted to encode major capsid proteins (MCPs) are additionally distinguished by red borders. Replication-associated genes (orange) and accessory genes with high-confidence functional predictions (green) are labelled, where applicable. Non-core genes of unknown function are coloured white. Homologous genes are connected by bars shaded in grayscale according to percent amino acid identity. Functionally conserved genes with sequence similarity below the similarity group threshold (Methods), or that are unconnected due to vertical ordering of the genomes, are instead connected by dashed lines. Protospacers matching published or predicted Thermococcales CRISPR spacers are indicated by vertical red rectangles. Top: AlphaFold3 structure of a representative kalavirus MCPs (with pTM score and coloured by PLDDT, blue to red) compared to the VP16 and VP17 MCPs of Bacteriophage P23-77. N-termini and C-termini are denoted by N and C, respectively.

Kalaviruses currently include two representatives, one predicted to be complete (29,199 bp) and the other near-complete (21,738 bp). Both family members encode two adjacent SJR MCPs, MCP-1 and MCP-2, structural homologs of the MCPs VP16 and VP17, respectively, from *Thermus thermophilus* phage P23-77 (family

Matsushitaviridae, order *Halopanivirales*) [30,31]. Similar to other members of the order *Halopanivirales*, kalaviruses encode a putative genome packaging ATPase of the FtsK-HerA superfamily. Other conserved genes are distributed throughout the genomes and encode largely proteins of unknown function, some with transmembrane domains. Several proteins with predicted helix-turn-helix (HTH) DNA binding domain, Zn finger and thioredoxin (TRX) domains, PD(D/E)XK nuclease, a probable Hop superfamily intein/homing endonuclease, RNA ligase, and a pectin lyase-like protein with right-handed β-helix topology that is similar to tailspike proteins observed in other viruses [32-34]. The two kalaviruses encode two variants of phage phi29-like PolB and hence are likely to replicate their linear genomes using a protein-primed mechanism [35]. The PolB of KalaV1 contains an intein, not uncommon in Thermococcales *polB* sequences; the PolB encoded by KalaV2 is shorter than most family B polymerases but maintains conserved active site motifs required for both proofreading and replication (Fig 4).

SECTION 6D: "*SHABALAVIRIDAE*"

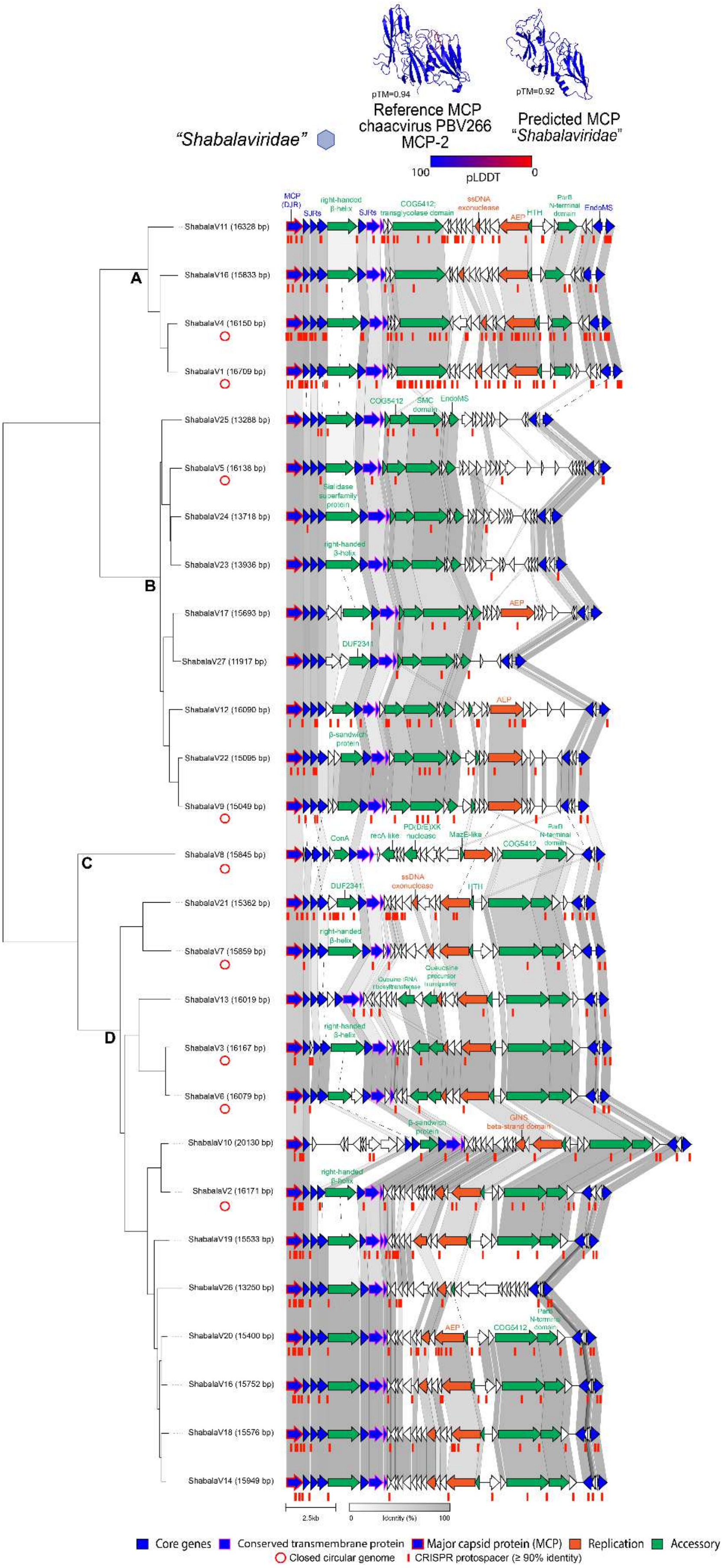

Supplementary Text Figure S6D. Genome synteny maps of representative shabalaviruses. Complete circular genomes are indicated by red circles. Predicted genes (coding sequences) are represented by arrows. Core genes (blue) are encoded by all complete or near-complete family members. Core genes predicted to encode major capsid proteins (MCPs) are additionally distinguished by red borders. Replication-associated genes (orange) and accessory genes with high-confidence functional predictions (green) are labelled, where applicable. Non-core genes of unknown function are coloured white. Homologous genes are connected by bars shaded in grayscale according to percent amino acid identity. Functionally conserved genes with sequence similarity below the similarity group threshold (Methods), or that are unconnected due to vertical ordering of the genomes, are instead connected by dashed lines. Protospacers matching published or predicted Thermococcales CRISPR spacers are indicated by vertical red rectangles. Top: AlphaFold3 structure of a representative shabalavirus MCP (with pTM score and coloured by PLDDT, blue to red) compared to the MCP-2 of chaacvirus PBV266. N-termini and C-termini are denoted by N and C, respectively.

Shabalaviruses are represented by eight complete circular genomes (15-16.7 kb) and 18 near-complete genomes, forming four clades: ShabalaA-D. Interestingly, of the nine genes that comprise the family-defining core of these viruses, seven display SJR or DJR topology, of which three produce high-ranking hits to viral structural proteins in our searches. The predicted DJR MCP is structurally similar to that of chaacviruses that target ANME-1 archaea [10] but contains a shorter N-terminal JR subdomain (chaacvirus PBV266 predicted MCP-2 is shown as a reference). The MCP gene is nested within the core module including eight other genes, six of which encode conserved SJR proteins that have no confident viral hits, except for one with a close but partial match to the triple jellyroll (TJR) turret protein C381 of Sulfolobus turreted icosahedral virus [36]. This conserved gene block is interrupted by 1 to 3 virus-specific genes. Most representatives (19/26; 73%) in this locus encode a right-handed β-helix-containing protein with structural similarity to mannuronan C5-epimerase domains within viral tail-spike proteins [32,33], suggesting that this protein is a structural component of the virion, potentially involved in host recognition. The remaining 7 contigs instead encode a DUF2341 ConA superfamily protein or a β-sandwich-containing protein that otherwise lacks high confidence homologs. Genes encoding proteins with COG5412-like domains are common across clades, both as small single domain proteins and as larger proteins with additional putative transglycosylase-like domains. Conversely, positionally conserved proteins with ParB-like N-terminal domains are widespread in all clades except ShabalaB, though like satanviruses and astarothviruses described above, no cognate ParA proteins were detected. As observed in other Thermococcales viruses, especially spindle-shaped viruses, shabalaviruses are variable in their replication modules, which can consist of MCM helicases or AEPs.

SECTION 6E: "*KARALIVIRIDAE*"

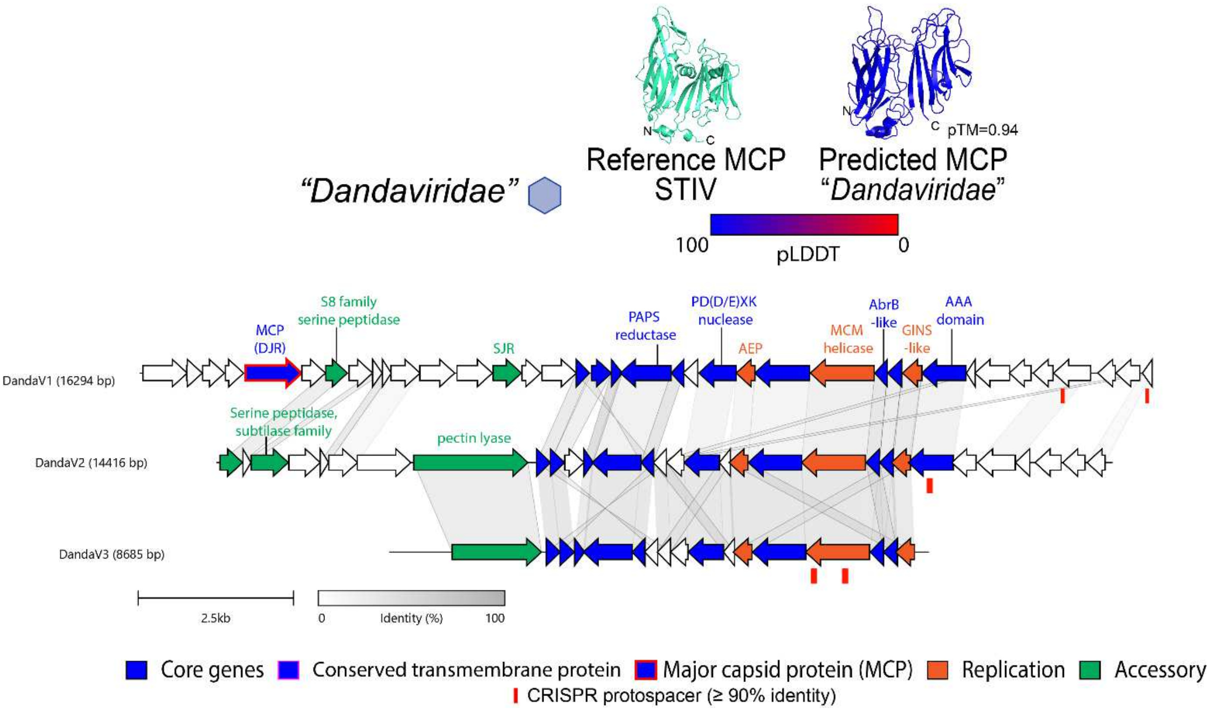

Supplementary Text Figure S6E. Genome synteny maps of representative karaliviruses. Complete circular genomes are indicated by red circles. Predicted genes (coding sequences) are represented by arrows. Core genes (blue) are encoded by all complete or near-complete family members. Core genes predicted to encode major capsid proteins (MCPs) are additionally distinguished by red borders. Replication-associated genes (orange) and accessory genes with high-confidence functional predictions (green) are labelled, where applicable. Non-core genes of unknown function are coloured white. Homologous genes are connected by bars shaded in grayscale according to percent amino acid identity. Functionally conserved genes with sequence similarity below the similarity group threshold (Methods), or that are unconnected due to vertical ordering of the genomes, are instead connected by dashed lines. Protospacers matching published or predicted Thermococcales CRISPR spacers are indicated by vertical red rectangles. Top: AlphaFold3 structure of a representative karalivirus MCP (with pTM score and coloured by PLDDT, blue to red) compared to the MCP of Haloarcula californiae icosahedral virus 1 (HCIV1). N-termini and C-termini are denoted by N and C, respectively.

Karaliviruses are represented by two highly similar complete circular genomes of 9,246 bp and 9,051 bp, respectively. Each genome encodes a highly divergent putative DJR MCPs. An adjacent conserved SJR protein displays high structural similarity to the stem domain of the P1 spike of bacteriophage PM2 [37]. Thus, we hypothesize that the SJR protein encoded next to the putative DJR MCP represents a structural protein of karaliviruses. Other functionally annotated proteins conserved in karaliviruses include a COG5412 domain-containing protein, a protein with best hits to PHROG7334, a PD(D/E)XK nuclease, and a PolB homolog, likely involved in genome replication (see main text and Fig. 4 for further discussion).

SECTION 6F: “*DANDAVIRIDAE”*

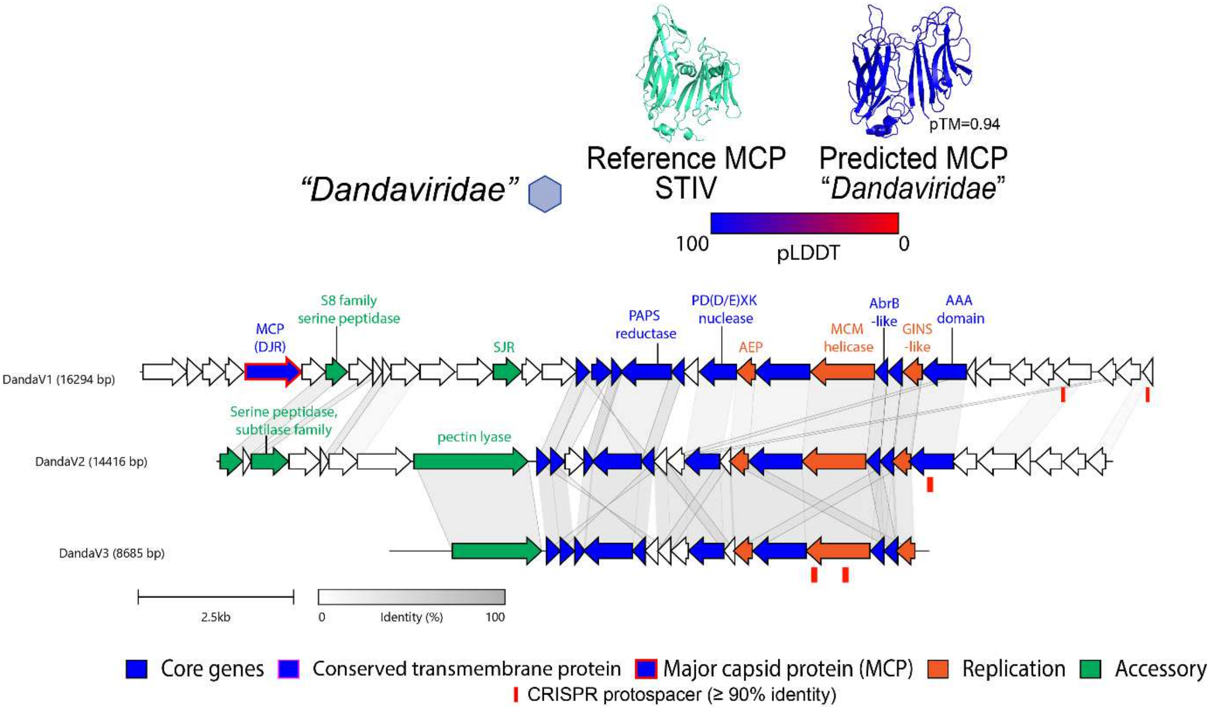

Supplementary Text Figure S6F. Genome synteny maps of representative dandaviruses. Predicted genes (coding sequences) are represented by arrows. Core genes (blue) are encoded by all family members. Core genes predicted to encode major capsid proteins (MCPs) are additionally distinguished by red borders. Replication-associated genes (orange) and accessory genes with high-confidence functional predictions (green) are labelled, where applicable. Non-core genes of unknown function are coloured white. Homologous genes are connected by bars shaded in grayscale according to percent amino acid identity. Functionally conserved genes with sequence similarity below the similarity group threshold (Methods), or that are unconnected due to vertical ordering of the genomes, are instead connected by dashed lines. Protospacers matching published or predicted Thermococcales CRISPR spacers are indicated by vertical red rectangles. Top: AlphaFold3 structure of a representative dandavirus MCP (with pTM score and coloured by PLDDT, blue to red) compared to the MCP of Sulfolobus turreted icosahedral virus (STIV).

Dandaviruses include three fragmented genomes ranging from 8.6-16.3 kb. While incomplete, these genomes share several conserved features that support their provisional grouping as a distinct viral family. A well-predicted DJR MCP is found in the longest genome but absent from the two shorter contigs. All three genomes share a long colinear and largely contiguous gene block encoding a PAPS reductase, PD(D/E)XK nuclease, AbrB-like transcriptional regulator, and three probable replication proteins (an AEP, MCM helicase, and a GINS-like protein). In two genomes, this region is flanked by an S8 family serine peptidase located near the MCP (where present) forming a putative structural module. Between the conserved modules, gene content is highly variable. One genome encodes several small uncharacterized ORFs; two others encode a large pectin lyase-like β-helix

protein, often associated with host surface degradation or receptor binding. Despite incomplete MCP representation, the conserved replication-morphogenesis block supports the hypothesis of a shared evolutionary lineage.

SECTION 6G: "*CHITRAGUPTAVIRIDAE*"

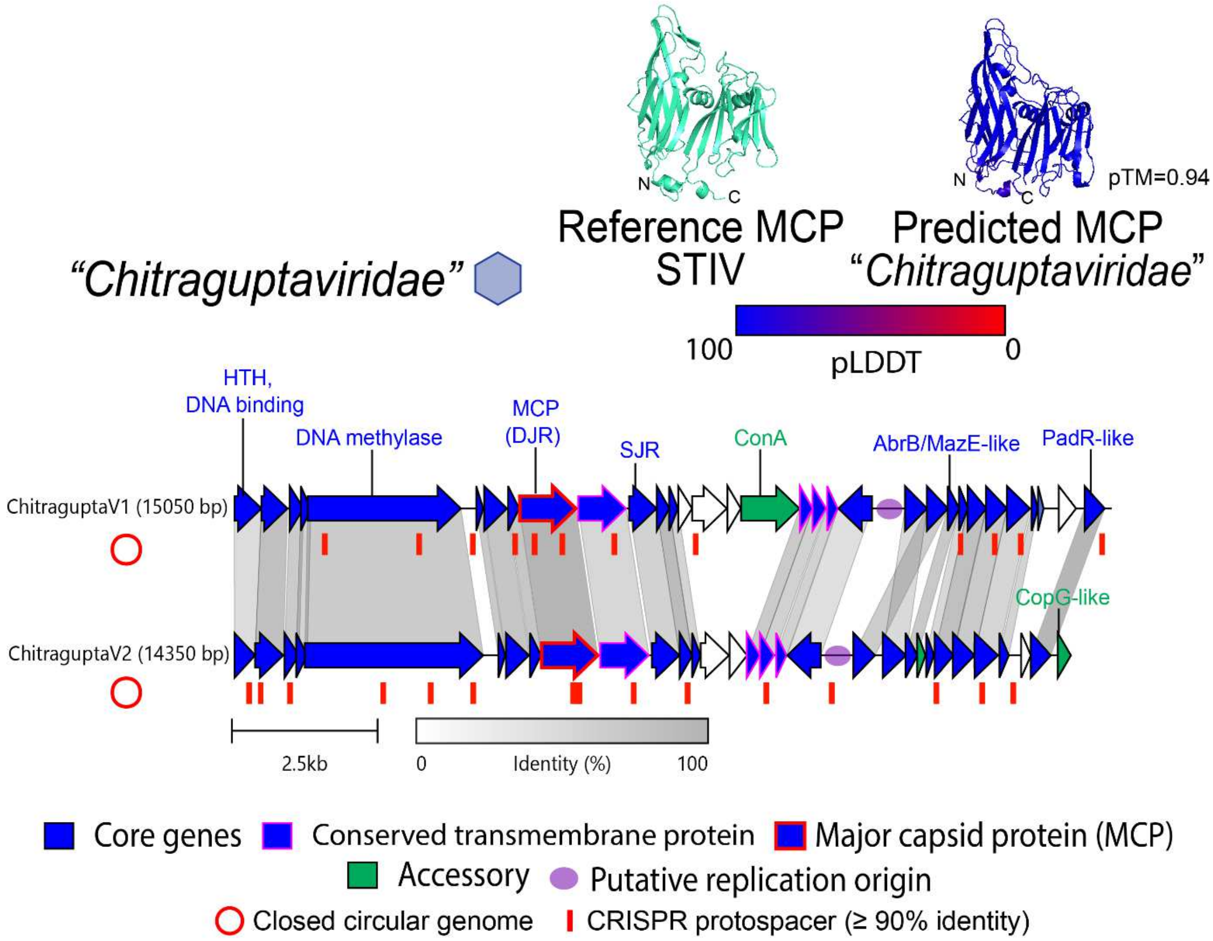

Supplementary Text Figure S6G. Genome synteny maps of representative chitraguptaviruses. Complete circular genomes are indicated by red circles. Predicted genes (coding sequences) are represented by arrows. Core genes (blue) are encoded by all complete or near-complete family members. Core genes predicted to encode major capsid proteins (MCPs) are additionally distinguished by red borders. Replication-associated genes (orange) and accessory genes with high-confidence functional predictions (green) are labelled, where applicable. Non-core genes of unknown function are coloured white. Homologous genes are connected by bars shaded in grayscale according to percent amino acid identity. Functionally conserved genes with sequence similarity below the similarity group threshold (Methods), or that are unconnected due to vertical ordering of the genomes, are instead connected by dashed lines. Protospacers matching published or predicted Thermococcales CRISPR spacers are indicated by vertical red rectangles. Putative replication origins are indicated by violet circles. Top: AlphaFold3 structure of a representative chitraguptavirus MCP (with pTM score and coloured by PLDDT, blue to red) compared to the MCP of Sulfolobus turreted icosahedral virus (STIV).

Chitraguptaviruses are represented by two complete circular genomes of 15,050 bp and 14,350 bp. Both viruses encode a DJR MCP structurally similar to that of STIV-like viruses, as well as an SJR fold protein likely functioning as the penton protein at the icosahedral five-fold vertices of icosahedral capsids [7]. Additional conserved genes include a DNA adenine methylase, several probable transcriptional regulators

(PadR-like, AbrB-like, HTH), and a triplet of predicted transmembrane proteins, possibly involved in virion morphogenesis or genome release. The two viruses differ in the presence of a ConA homolog and several genes of unknown function. Both genomes contain large (559 bp and 567 bp) intergenic regions flanked by genes in divergent orientation and may represent the origin of replication (purple ovals). OriV-Finder analysis only suggests this to be the case for ChitraguptaV2 (14350 bp) but given their similar lengths and near-identical genomic contexts, it is likely that the same is true for the other virus genome as well.

References

1 Geslin, C. *et al.* PAV1, the first virus-like particle isolated from a hyperthermophilic euryarchaeote, "*Pyrococcus abyssi*". *J Bacteriol* **185**, 3888–3894 (2003). https://doi.org/10.1128/JB.185.13.3888-3894.2003

2 Gorlas, A., Koonin, E. V., Bienvenu, N., Prieur, D. & Geslin, C. TPV1, the first virus isolated from the hyperthermophilic genus *Thermococcus*. *Environ Microbiol* **14**, 503–516 (2012). https://doi.org/10.1111/j.1462-2920.2011.02662.x

3 Ward, D. E. *et al.* Characterization of plasmid pRT1 from *Pyrococcus sp*. strain JT1. *J Bacteriol* **184**, 2561–2566 (2002). https://doi.org/10.1128/JB.184.9.2561-2566.2002

4 Strunnikov, A. V. & Jessberger, R. Structural maintenance of chromosomes (SMC) proteins. *European Journal of Biochemistry* **263**, 6–13 (1999). https://doi.org/https://doi.org/10.1046/j.1432-1327.1999.00509.x

5 Duan, C. *et al.* Diversity of Bathyarchaeia viruses in metagenomes and virus-encoded CRISPR system components. *ISME Commun* **4**, ycad011 (2024). https://doi.org/10.1093/ismeco/ycad011

6 Reiter, W. D. *et al.* Identification and Characterization of the genes encoding 3 structural proteins of the *Sulfolobus* virus-like particle SSV1. *Mol Gen Genet* **206**, 144–153 (1987). https://doi.org/Doi 10.1007/Bf00326550

7 Baquero, D. P. *et al.* Structure and assembly of archaeal viruses. *Adv Virus Res* **108**, 127–164 (2020). https://doi.org/10.1016/bs.aivir.2020.09.004

8 Liu, Y. *et al.* Diversity, taxonomy, and evolution of archaeal viruses of the class *Caudoviricetes*. *PLoS Biol* **19**, e3001442 (2021). https://doi.org/10.1371/journal.pbio.3001442

9 Zhou, Y. *et al.* Viruses and virus satellites of haloarchaea and their nanosized DPANN symbionts reveal intricate nested interactions. *Nat Microbiol* **10**, 2673–2685 (2025). https://doi.org/10.1038/s41564-025-02149-7

10 Laso-Perez, R. *et al.* Evolutionary diversification of methanotrophic ANME-1 archaea and their expansive virome. *Nat Microbiol* **8**, 231–245 (2023). https://doi.org/10.1038/s41564-022-01297-4

11 Martin, K. A., Friedman, S. A. & Austin, S. J. Partition site of the P1 plasmid. *Proceedings of the National Academy of Sciences* **84**, 8544–8547 (1987). https://doi.org/doi:10.1073/pnas.84.23.8544

12 Gu, Y. *et al.* Bacterial Shedu immune nucleases share a common enzymatic core regulated by diverse sensor domains. *Mol Cell* **85**, 523–536.e526 (2025). https://doi.org/10.1016/j.molcel.2024.12.004

13 Xu, S. Y. *et al.* Characterization of type II and III restriction-modification systems from *Bacillus cereus* strains ATCC 10987 and ATCC 14579. *J Bacteriol* **194**, 49–60 (2012). https://doi.org/10.1128/JB.06248-11

14 Reader, J. S., Metzgar, D., Schimmel, P. & de Crécy-Lagard, V. Identification of four genes necessary for biosynthesis of the modified nucleoside queuosine. *J Biol Chem* **279**, 6280–6285 (2004). https://doi.org/10.1074/jbc.M310858200

15 Hutinet, G. *et al.* 7-Deazaguanine modifications protect phage DNA from host restriction systems. *Nat Commun* **10**, 5442 (2019). https://doi.org/10.1038/s41467-019-13384-y

16 Heyer, W. D., Rao, M. R., Erdile, L. F., Kelly, T. J. & Kolodner, R. D. An essential *Saccharomyces cerevisiae* single-stranded DNA binding protein is

homologous to the large subunit of human RP-A. *EMBO J* **9**, 2321–2329 (1990). https://doi.org/10.1002/j.1460-2075.1990.tb07404.x
17 Woodman, I. L. & Bolt, E. L. Molecular biology of Hel308 helicase in Archaea. *Biochem Soc Trans* **37**, 74–78 (2009). https://doi.org/10.1042/BST0370074
18 Krupovic, M. *et al. Adnaviria*: a new realm for archaeal filamentous viruses with linear A-form double-stranded DNA genomes. *J Virol* **95**, e0067321 (2021). https://doi.org/10.1128/JVI.00673-21
19 Krupovic, M. *et al.* ICTV Virus Taxonomy Profile: *Adnaviria* 2025. *J Gen Virol* **106** (2025). https://doi.org/10.1099/jgv.0.002091
20 Mochizuki, T. *et al.* Diversity of viruses of the hyperthermophilic archaeal genus Aeropyrum, and isolation of the *Aeropyrum pernix* bacilliform virus 1, APBV1, the first representative of the family *Clavaviridae*. *Virology* **402**, 347–354 (2010). https://doi.org/10.1016/j.virol.2010.03.046
21 Ptchelkine, D. *et al.* Unique architecture of thermophilic archaeal virus APBV1 and its genome packaging. *Nat Commun* **8**, 1436 (2017). https://doi.org/10.1038/s41467-017-01668-0
22 Wang, F. *et al.* Spindle-shaped archaeal viruses evolved from rod-shaped ancestors to package a larger genome. *Cell* **185**, 1297–1307 e1211 (2022). https://doi.org/10.1016/j.cell.2022.02.019
23 Huang, L., Wang, H. & Ictv Report, C. ICTV Virus Taxonomy Profile: *Ovaliviridae*. *J Gen Virol* **102** (2021). https://doi.org/10.1099/jgv.0.001546
24 Liu, Y. *et al.* New archaeal viruses discovered by metagenomic analysis of viral communities in enrichment cultures. *Environ Microbiol* **21**, 2002–2014 (2019). https://doi.org/10.1111/1462-2920.14479
25 Cossu, M. *et al.* Flipping chromosomes in deep-sea archaea. *PLoS Genet* **13**, e1006847 (2017). https://doi.org/10.1371/journal.pgen.1006847
26 Gaudin, M. *et al.* Extracellular membrane vesicles harbouring viral genomes. *Environ Microbiol* **16**, 1167–1175 (2014). https://doi.org/10.1111/1462-2920.12235
27 Demina, T. A. *et al.* HCIV-1 and other tailless icosahedral internal membrane-containing viruses of the family *Sphaerolipoviridae*. *Viruses* **9** (2017). https://doi.org/10.3390/v9020032
28 Demina, T. A. *et al.* Archaeal *Haloarcula californiae* icosahedral virus 1 highlights conserved elements in icosahedral membrane-containing DNA viruses from extreme environments. *mBio* **7** (2016). https://doi.org/10.1128/mBio.00699-16
29 Makarova, K. S. *et al.* Dark matter in archaeal genomes: a rich source of novel mobile elements, defense systems and secretory complexes. *Extremophiles* **18**, 877–893 (2014). https://doi.org/10.1007/s00792-014-0672-7
30 Jalasvuori, M. *et al.* The closest relatives of icosahedral viruses of thermophilic bacteria are among viruses and plasmids of the halophilic archaea. *J Virol* **83**, 9388–9397 (2009). https://doi.org/10.1128/JVI.00869-09
31 Jaatinen, S. T., Happonen, L. J., Laurinmäki, P., Butcher, S. J. & Bamford, D. H. Biochemical and structural characterisation of membrane-containing icosahedral dsDNA bacteriophages infecting thermophilic *Thermus thermophilus*. *Virology* **379**, 10–19 (2008). https://doi.org/10.1016/j.virol.2008.06.023

32 Dunstan, R. A. *et al.* Mechanistic insights into the capsule-targeting depolymerase from a Klebsiella pneumoniae bacteriophage. *Microbiol Spectr* **9**, e0102321 (2021). https://doi.org/10.1128/Spectrum.01023-21
33 Liu, S. *et al.* Discovery, structural characteristics and evolutionary analyses of functional domains in Acinetobacter baumannii phage tail fiber/spike proteins. *BMC Microbiol* **25**, 73 (2025). https://doi.org/10.1186/s12866-025-03790-2
34 Menéndez-Conejero, R. *et al.* Structure of a reptilian adenovirus reveals a phage tailspike fold stabilizing a vertebrate virus capsid. *Structure* **25**, 1562–1573.e1565 (2017). https://doi.org/10.1016/j.str.2017.08.007
35 Blanco, L. & Salas, M. Characterization and purification of a phage phi 29-encoded DNA polymerase required for the initiation of replication. *Proc Natl Acad Sci U S A* **81**, 5325–5329 (1984). https://doi.org/10.1073/pnas.81.17.5325
36 Maaty, W. S. *et al.* Characterization of the archaeal thermophile Sulfolobus turreted icosahedral virus validates an evolutionary link among double-stranded DNA viruses from all domains of life. *J Virol* **80**, 7625–7635 (2006). https://doi.org/10.1128/JVI.00522-06
37 Abrescia, N. G. *et al.* Insights into virus evolution and membrane biogenesis from the structure of the marine lipid-containing bacteriophage PM2. *Mol Cell* **31**, 749–761 (2008). https://doi.org/10.1016/j.molcel.2008.06.026
